# Pro-Arrhythmic Effects of Discontinuous Conduction at the Purkinje Fiber-Ventricle Junction Arising From Heart Failure-Induced Ionic Remodeling – Insights From Computational Modelling

**DOI:** 10.3389/fphys.2022.877428

**Published:** 2022-04-25

**Authors:** Kun Jian, Chen Li, Jules C. Hancox, Henggui Zhang

**Affiliations:** ^1^ Biological Physics Group, Department of Physics and Astronomy, The University of Manchester, Manchester, United Kingdom; ^2^ School of Physiology, Pharmacology and Neuroscience, Medical Sciences Building, University Walk, Bristol, United Kingdom; ^3^ Key Laboratory of Medical Electrophysiology of Ministry of Education and Medical Electrophysiological Key Laboratory of Sichuan Province, Institute of Cardiovascular Research, Southwest Medical University, Luzhou, China

**Keywords:** heart failure, ventricle, Purkinje fiber, electrical remodeling, conduction block, computational modelling, conduction safety

## Abstract

Heart failure is associated with electrical remodeling of the electrical properties and kinetics of the ion channels and transporters that are responsible for cardiac action potentials. However, it is still unclear whether heart failure-induced ionic remodeling can affect the conduction of excitation waves at the Purkinje fiber-ventricle junction contributing to pro-arrhythmic effects of heart failure, as the complexity of the heart impedes a detailed experimental analysis. The aim of this study was to employ computational models to investigate the pro-arrhythmic effects of heart failure-induced ionic remodeling on the cardiac action potentials and excitation wave conduction at the Purkinje fiber-ventricle junction. Single cell models of canine Purkinje fiber and ventricular myocytes were developed for control and heart failure. These single cell models were then incorporated into one-dimensional strand and three-dimensional wedge models to investigate the effects of heart failure-induced remodeling on propagation of action potentials in Purkinje fiber and ventricular tissue and at the Purkinje fiber-ventricle junction. This revealed that heart failure-induced ionic remodeling of Purkinje fiber and ventricular tissue reduced conduction safety and increased tissue vulnerability to the genesis of the unidirectional conduction block. This was marked at the Purkinje fiber-ventricle junction, forming a potential substrate for the genesis of conduction failure that led to re-entry. This study provides new insights into proarrhythmic consequences of heart failure-induced ionic remodeling.

## Introduction

Heart Failure is a common long-term progressive and serious medical condition with high mortality. Although HF is highly pro-arrhythmic (Masarone et al., 2017; Dean and Lab, 1989; Aistrup et al., 2011), the underlying mechanisms of pro-arrhythmia in HF are incompletely understood. Abundant data suggest that HF is associated with remodelling of the expression and kinetics of some ion channels and transporters responsible for the action potential of cardiac cells (for details of summary *see* Tables 1–4). However, the extent to which such ionic remodelling can account for the pro-arrhythmic effects of HF is unclear, as the nonlinear complexity of the heart impedes a detailed experimental analysis of cardiac excitations at the subcellular, cellular to tissue levels. Over the last two decades, significant progress has been made in the development of biophysically detailed computational models of the heart (Trayanova, 2011; Hill et al., 2016; Krogh-Madsen et al., 2016; Trayanova et al., 2017; Clerx et al., 2019; Zhang et al., 2020). This provides a quantitative framework to evaluate the pro-arrhythmic effects of HF-induced electrical remodelling.

**TABLE 1:** List of updates of the Benson *et al.* (2008) model under CTL condition.

Ion channel and transporter	Modifications	File for details and experimental data for justification of the model updates
Inward rectifier K^+^ current: I_K1_	The maximum channel conductance of *I*_*K1*_ was scaled by 0.7 for Endo, 1.1 for M and 1.0 for Epi cell models respectively	Supplementary Text S1.1.1
Slow delayed rectifier K^+^ current: I_Ks_	The maximum channel conductance of *I*_*Ks*_ was scaled by 0.59 for Endo, 0.68 for M and 1.0 for Epi cell models respectively	Supplementary Text S1.1.2
Intracellular Ca^2+^ transient: (Myerburg et al., 1978)	The maximum uptake from the myoplasmic to the network sarcoplasmic reticulum (NSR) and the myoplasmic volume were reduced by 20 and 50% respectively	Supplementary Text S1.1.3

**TABLE 2:** List of updates of the Benson *et al.* (2008) model under HF condition.

Ion channel and transporter	Modifications	File for details and experimental data for justification of the model updates
Fast Na^+^ current: I_Na_	The maximum conductance of *I*_*Na*_ was reduced by 32%	Supplementary Text S1.2.1
Late Na^+^ current: I_NaL_	The maximum conductance and the inactivation time constant were increased by 30 and 34% respectively	Supplementary Text S1.2.2
Transient outward K^+^ current: I_to1_	The maximum conductance of *I*_*to1*_ was reduced by 43% in the Endo and Epi cell models and 45% in the M cell model	Supplementary Text S1.2.3
Inward rectifier K^+^ current: I_K1_	The maximum conductance was reduced by 41.1, 40.7 and 40.9% in the Endo, M and Epi cell models respectively	Supplementary Text S1.2.4
Slow delayed rectifier K^+^ current: I_Ks_	The *I*_*Ks*_ current was reduced by 30% and the transmural heterogeneity of *I*_*Ks*_ among the Endo, M and Epi cell models was removed	Supplementary Text S1.2.5
L-type Ca^2+^ current: I_CaL_	The V_0.5_ of the steady state activation curve of *I*_*CaL*_ was shifted by 7.64 mV to the left	Supplementary Text S1.2.6
Intracellular Ca^2+^ transient: [Ca^2+^] (Myerburg et al., 1970)_i_	The maximal sarcoplasmic reticulum Ca^2+^-ATPase (SERCA) uptake was reduced by 87.5%. SR leak was reduced by 35%. JSR release was reduced by 40%. The NCX density was increased by 20%	Supplementary Text S1.2.7
Na^+^/K^+^-ATPase: I_NaK_	The maximum *I*_*NaK*_ was reduced by 40%	Supplementary Text S1.2.8

**TABLE 3:** List of updates of Aslanidi *et al.* (2009) model under CTL condition.

Ion channel and transporter	Modifications	File for details and experimental data for justification of the model updates
Fast Na^+^ current: I_Na_	The V_0.5_ of the steady state curve of the activation was shifted by 10 mV to the right and that of the inactivation by 10 mV to the left. The maximum conductance of *I*_Na_ was increased by 3%	Supplementary Text S1.3.1
Transient outward K^+^ current: I_to1_	The V_0.5_ of the steady states activation and inactivation curves were both shifted by 2 mV to the right. The fast inactivation time constant (τ_fast_) was refitted	Supplementary Text S1.3.2
Inward rectifier K^+^ current: I_K1_	The model was re-built by a simple voltage-dependent formulation as shown in Eqs S1.1–2 in Supplementary Text S1	Supplementary Text S1.3.3
Slow delayed rectifier K^+^ current: I_Ks_	The V_0.5_ of the activation steady-state variable of *I*_*Ks*_ was shifted by 4.50 mV to the left and the maximum conductance was reduced by 15%	Supplementary Text S1.3.4
L-type Ca^2+^ current: I_CaL_	The V_0.5_ of the steady-state curve for activation and inactivation was shifted by 2.26 and 6.10 mV to the left respectively. The voltage-dependent fast (τ_f_) and slow (τ_f2_) time constant curves of the inactivation were shifted by 3.59 mV to the left and 0.20 mV to the right respectively. The maximum conductance of *I*_*CaL*_ was increased by 12%	Supplementary Text S1.3.5

**TABLE 4:** List of updates of Aslanidi *et al.* (2009) model under HF condition.

Ion channel and transporter	Modifications	File for details and experimental data for justification of the model updates
Fast Na^+^ current: I_Na_	The maximum conductance of *I*_*Na*_ was reduced by 40%	Supplementary Text S1.4.1
Transient outward K^+^ current: I_to1_	The fast inactivation voltage-dependent time constant curve (τ_fast_) was shifted by 1 mV rightward and the peak value of the slow inactivation time constant (τ_slow_) was reduced by 2.50 ms. The maximal channel conductance was reduced by 30%	Supplementary Text S1.4.2
Inward rectifier K^+^ current: I_K1_	The voltage-dependent channel conductance ( $G_{K1}$ ) was shifted by 17.50 mV to the right. The maximum conductance was increased by 5%	Supplementary Text S1.4.3
Fast and slow delayed rectifier K^+^ current: I_Kr_ and I_Ks_	The maximum channel conductance of I_Kr_ was reduced by 10%. The stead state of I_Ks_ was shifted by 6 mV to the right and its maximum conductance was reduced by 5.7%	Supplementary Text S1.4.4
L-type Ca^2+^ current: I_CaL_	The V_0.5_ of the steady states of the activation and inactivation curves were shifted by 2.30 and 2.40 mV to the left respectively. The voltage-dependent fast and slow time constant curves of the inactivation were shifted by 22.90 and 13.60 mV upward	Supplementary Text S1.4.5
T-type Ca^2+^ current: I_CaT_	The maximum conductance of *I*_*CaT*_ was reduced by 10%. The V_0.5_ of the activation and inactivation steady-state curves were shifted by 1.20 and 3.00 mV to the left respectively	Supplementary Text S1.4.6

Fast and stable conduction of electrical excitation waves in the conduction pathways of cardiac tissue is essential to maintain the normal function of the heart. However, altered cellular electrophysiology properties of Purkinje fibers and ventricular cells arising from pathological conditions may impair such conduction; altered conduction delay at the Purkinje fiber-ventricle (PVJ) junction may facilitate unsafe discontinuous conduction, conduction failure or the formation of re-entrant excitation (Gilmour and Watanabe, 1994; Li et al., 1994; Wiedmann et al., 1996; Morley et al., 2005; Scheinman, 2009; Walton et al., 2014). It has previously been shown that changes in the density and electrical resistance of PVJ helps to sustain re-entrant excitation waves (Behradfar et al., 2014). Additionally, HF-induced ionic remodelling in Purkinje fibers (PFs) might lead to frequent occurrence of early afterdepolarizations which could be attributable to prolonged action potential duration (Logantha et al., 2021). Dysfunction in the intracellular Ca^2+^ handling can facilitate PF arrythmias (Boyden et al., 2010), whilst slow conduction of excitation waves in HF facilitate re-entrant waves according to the “gate hypothesis” (Lazzara et al., 1976). However, pro-arrhythmic effects arising from the discontinuous conduction at the PVJ are still incompletely understood, especially in the HF condition in which conduction failure at the PVJ may occur (Overholt et al., 1984; Wiedmann et al., 1996; Aslanidi et al., 2009).

The aim of this study was to investigate the pro-arrhythmic effects of HF on cardiac APs and propagation in PFs, ventricular tissue and at the PVJ. Single canine PF and ventricular cell models were developed for control and HF conditions. The developed cellular models were then incorporated into one-dimensional strand and three-dimensional wedge models of the intact PF-ventricle to investigate the effects of HF-induced remodelling on AP propagation in the canine PF, ventricular tissue and at the PVJ to explore the proarrhythmic effects of HF.

## Model Development

### Abbreviations

AP: Action potential.

APA: Action potential amplitude.

APD: Action potential duration.

BCL: Basic cycle length.

CV: Conduction velocity.

CTL: Control condition.

DT-MRI: Diffusion tensor magnetic resonance imaging.

EADs: Early afterdepolarizations.

ERP: Effective refractory period.

HF: Heart failure.

MUV: Maximum upstroke velocity.

1D: One-dimensional (1D).

OS: Overshoot (OS) of action potential.

PP: Plateau potential.

PF: Purkinje fiber.

PVJ: Purkinje fiber-ventricle junction.

RMP: Resting membrane potential.

SF: Safety factor (SF).

3D: Three-dimensional.

VW: Vulnerable window.

### Single Cell Models

Two families of canine ventricular and PF cell models were developed under both CTL and HF conditions. One is based on updating and modification of the cellular models for canine ventricular cells developed by Benson *et al.* (2008) (*see* Supplementary Text S1.1 for CTL and Supplementary Text S1.2 for HF ventricular cell model development), and the other is based on modification of the canine PF cell model developed by Aslanidi *et al.* (2009) (*see* Supplementary Text S1.3 for CTL and Supplementary Text S1.4 for HF PF cell model development). The modifications to the basal models of ventricular and PF cells are summarised in Tables 1–4, which summarizes all changes made in simulating the electrophysiological properties of the ventricular and PF cells based on comprehensive reviews of extant experimental data. Further details about the HF-induced changes in the electrophysiological properties of the cell models can be found in the Supplementary Tables S1–S8. Justification and comparison of the simulation results for HF-induced change in ionic properties to experimental data are shown in Supplementary Figures S1–S9. Detailed equations, parameters and initial conditions for the ventricular and PF cell models are presented in Supplementary Text S1 respectively.

Action potentials in each cell model were evoked by an external stimulus with a magnitude of -45.0 pA/pF and duration of 1 ms. All simulations were performed at a BCL of 1000 ms and for a duration of 100 s. For each of the simulations, the duration of the AP was measured after numerical solution of the model reached its steady state. All APDs mentioned in this study refer to APD_90_.

### Tissue Models

#### One-Dimensional Model of the Ventricular Wall

We have developed two sets of 1D models: a ventricle-only strand and an intact PF-ventricle strand. The PF strand had a length of 18 mm and the ventricular strand had a length of 15 mm (Vassalle and Lee, 1984) with equal-proportion segments of Endo, M and Epi cells (Yan et al., 1998). Both models had a spatial resolution of 0.15 mm. The cardiac excitation propagation in the tissue model was described using the mono-domain equation (Benson et al., 2008; Clayton et al., 2011). In this study, the diffusion coefficient (*D*) in the tissue model was set to 0.500 mm^2^/ms for the PF and 0.110 mm^2^/ms for the ventricle in the CTL condition, which resulted in a transmural CV of 1.50 m/s in the PF and 0.44 m/s in the ventricle that matched experimental observations (Clerc, 1976; Myerburg et al., 1978; Rosen et al., 1981; Joyner and Overholt, 1985; Cragun et al., 1997; Yan et al., 1998; Akar et al., 2007). At the PVJ, a linear interpolation of the diffusion parameter of the ventricular strand and the PF strand was implemented to avoid a stepwise change of the intercellular electrical coupling at the junction. At the border of the M-EPI, a five-fold of decrease in the diffusion parameter was implemented following the previous studies of Gima and Rudy (Gima and Rudy, 2002) and Zhang et al. (2008).

In the HF condition, there are experimental data showing that the CV is decreased by 30% in the PF and 41% in the ventricle (Akar et al., 2007; Maguy et al., 2009). Such reduced CV may also be associated with changes in the expression of connexins (Akar et al., 2004; Poelzing and Rosenbaum, 2004; Severs et al., 2004; Wiegerinck et al., 2006) responsible for intercellular electrical coupling. To reproduce these experimental observations in CV in simulations of HF, *D* was reduced to 0.310 mm^2^/ms for the PF and to 0.073 mm^2^/ms for the ventricle, which gave a transmural CV of 1.05 m/s in the PF (30% reduction compared to CTL condition) and 0.26 m/s in the ventricle (40% reduction compared to CTL condition).

In simulations using the 1D strand model, a series of external pacing stimuli were applied (at time interval of 1000 ms and with a duration of 2 ms) at the Endo end or the free-running end of the PF.

#### Three-Dimensional Wedge Model of the Purkinje-Ventricular Junction

We developed two sets of 3D wedge geometrical models of Purkinje-ventricular coupling: one with a single idealised PF strand coupled to the ventricle wedge and one with two idealised PF strands coupled to the wedge. The first model consisted of a ventricular wedge with a dimension of 15 mm × 20 mm × 20 mm, and a single PF strand with a length of 18 mm and width of 2.1 mm connecting perpendicularly to the Endo surface. The ventricle wedge geometry had a spatial resolution of 0.35 mm, which was based on the reconstruction from DT-MRI and has been successfully used for simulations of the transmural propagation in canine ventricles by Benson et al. (2008). The geometry of the PF strand was idealised as the DT-MRI data does not contain the PF network. The use of idealised PF strand allowed us to investigate the effects of varying the width of PF on the conduction of excitation waves at the PVJ. The second model used the same 3D wedge geometry data of the ventricle (i.e., the same 3D geometry with the same sheet structure and fiber orientation), but with a dimension of 15 mm × 20 mm × 40 mm, and dual idealised PF strands, with one having a relative wider thickness (2.8 mm) than the other having a thickness of 1.4 mm.

In the 3D models of the CTL condition, to take into considerations of anisotropic conduction of excitation waves in cardiac tissue, diffusion tensor ***D*** were set as *D*_**||**_ = 0.600 mm^2^/ms and *D*_⊥_ = 0.150 mm^2^/ms to produce the CV in approximately 1.20 m/s along fibers and 0.44 m/s orthogonally to fibers in ventricle to match experimental observations (Durrer et al., 1955; Swain and Weidner, 1957; Joyner and Overholt, 1985; Yan et al., 1998; Bauer et al., 2001; Akar et al., 2007). In the HF condition, ***D*** were set as *D*_**||**_ = 0.292 mm^2^/ms and *D*_⊥_ = 0.073 mm^2^/ms to produce the CV approximately 0.71 m/s along fibers and 0.26 m/s orthogonally to fibers, resulting a 41% reduction in both directions (Akar et al., 2007; Maguy et al., 2009). The diffusion coefficient in PF was set to the same value as in 1D strand model, which gave 1.50 m/s CV in CTL condition and 1.05 m/s in HF condition.

Electrical excitation waves were evoked by applying an external stimulus (2 ms in duration) to the free running end of the PF.

#### Safety Factor

The formula for calculating SF was proposed by Shaw & Rudy (Shaw and Rudy, 1997). This quantifies the ratio between the charges produced by a cell excitation and the charges that the cell requires to be excited. By this definition, SF > 1 suggests a successful conduction; (SF ∼ 1) implicates a marginal conduction safety; SF < 1 suggests that a conduction failure is very likely to occur. At any given point in the cardiac tissue, SF is computed by equation below:

$$SF=\frac{\int_{A}I_{c}\cdot dt+\int_{A}I_{out}\cdot dt}{\int_{A}I_{in}\cdot dt}\;\;A|t\in \left[t_{1\text{\%}},\;t_{V_{max}}\right]$$

Where $I_{c}$ is the capacitive current, $I_{out}$ the intercellular current from the cell to its downstream neighbour, $I_{in}$ the intercellular current from its upstream neighbour to the cell, dt the time step and A the time course, over which net membrane charges are accumulated. A was defined from the time at which the membrane potential derivative $\left(\frac{\partial V_{m}}{\partial t}\right)$ reached 1% of its maximum value, to the time at which $V_{m}$ reached its maximum $\left(V_{max}\right)$ (Romero et al., 2005).

#### Vulnerable Window

The vulnerability of cardiac tissue to unidirectional block was defined as the duration of a specific time period - the vulnerable window, during which a premature S2 stimulus applied to the tail of the refractory wave evoked by a S1 stimulus resulted in unidirectional conduction, due to asymetrically incomplete recovery of the tissue from the previous excitation of S1 in the antegrade and retrograde directions (Starmer, 2007). Such unidirectional conduction facilitates the formation of re-entrant excitation wave in 2D and 3D dimensional tissues; as such the width of the measured specific time period was measured to characterise the vulnerability of the tissue to the genesis of arrhythmias (Starmer, 2007). In simulations, the S1 stimulus was applied at the end of the PF strand to evoke the conditional wave, and the S2 was applied at a local site of the 1D ventricular strand for measuring the VW at that point. The S2 stimulus site varied from one end of the ventricular strand to the other end for measuring the VW across the transmural ventricular strand. In cardiac tissue, unidirectional conduction block predisposes to the initiation of re-entry (Frame et al., 1987; Qu et al., 2006).

#### Numerical Implementation

At single cell level, all gating and state variables were solved by forward Euler method with a time step of 0.005 ms. At tissue level, Crank-Nicolson scheme (Crank and Nicolson, 1947) was implemented to solve the PDE models of cardiac tissue with a space step of 0.15 mm for 1D strands, and of 0.35 mm for the 3D ventricle wedge, and a time step of 0.005 ms. At tissue boundaries, Neumann boundary conditions with zero-flux was implemented. All simulations were carried out on a system with two Intel Xeon E5 2680v2 10 core processors (40 logical cores) and 128 GB RAM memory, and OpenMP (http://www.openmp.org/resources/openmp-compilers/) was implemented for parallelisation.

## Results

### Single Cell Simulations

#### Simulated Action Potentials in the CTL Condition

**FIGURE 1:**
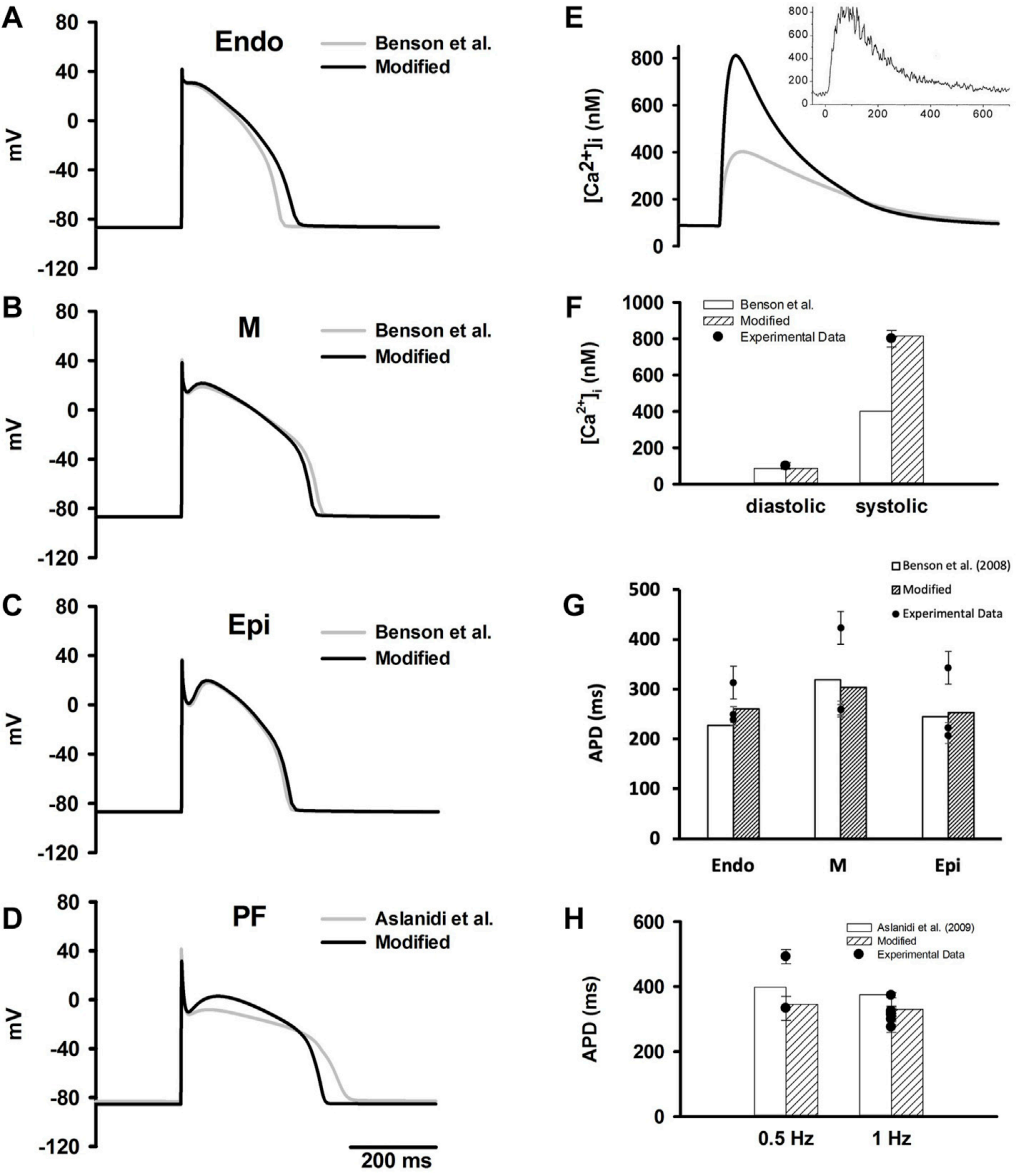
**(A**–**D)** Simulated time courses of APs in ENDO, M, EPI and PF cell models which were compared to the original Benson et al. model (2008) and Aslanidi et al. model (2009). **(E)** Simulated time courses of [Ca^2+^]_i_ during an evoked AP in the M cell model in comparison to experimental data from O’Rourke et al. (O'Rourke et al., 1999). **(F)** Computed diastolic and systolic [Ca^2+^]_i_ from the modified models in comparison to the original Benson et al. (2008) models and experimental data (O'Rourke et al., 1999). **(G)** Computed APDs at 1 Hz in ventricular cell models in comparison to the original Benson et al. (2008) models and experimental data (experimental data are listed in Supplementary Tables S10–S12). **(H)** Computed APDs at different simulation rates PF cell model in comparison to the original Aslanidi et al. model (2009) model and experimental data (experimental data are listed in Supplementary Table S13).

Figures 1A–D shows the computed time courses of APs from our models in comparison to those computed from the original models (the Benson *et al.* (2008) models and the Aslanidi et al. model (2009)). Our modifications resulted in some change to the APDs as shown in Table 5, with values within the experimental data range.

**TABLE 5:** Computed APDs in the Endo, M, Epi and PF cell models compared to the Benson et al. model (2008) and Aslanidi et al. model (2009).

	Endo	M	Epi	PF
Experimental range	238.6–313 ms	258.7–423 ms	207–343 ms	275–373 ms
Original models	227.3 ms	319.0 ms	244.9 ms	375.0 ms
Updated models	260.3 ms	303.0 ms	252.8 ms	330.2 ms
File for experimental data	Supplementary Table S9	Supplementary Table S10	Supplementary Table S11	Supplementary Table S12

For ventricular cells, the modifications resulted in an increased peak [Ca^2+^]_i_ with a value about 813 nM, which was consistent with reported experimental data (O'Rourke et al., 1999) (Figures 1E,F). Additionally, simulated APDs of the modified models more closely matched experimental data at different stimulation rates (*see* Figure 1G).

As compared with the Aslanidi *et al.* (2009) model, the AP of our PF cell model exhibited a shorter APD which well matched the experimental data (Figure 1H). The AP characteristics of our PF model were also validated against experimental data, including membrane resting potential, APA, OS, MUV and PP. The results were plotted in Supplementary Figures S10C–G together with the experimental data shown as dots.

### Simulated Action Potentials in the HF Condition

#### Ventricular Cell Models

**FIGURE 2:**
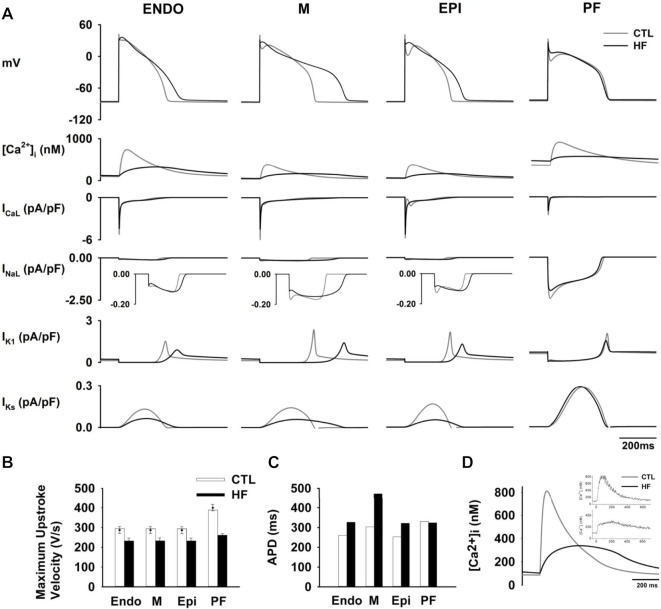
**(A)** Computed time courses of APs and HF-remodelled ionic currents in the CTL and HF conditions for ventricular and PF cell models. Five major HF-remodelled ionic currents were shown (*see* Supplementary Text S1). **(B)** Maximum upstroke velocity. Experimental data (Balati et al., 2000; Maguy et al., 2009; Hegyi et al., 2019) were shown in solid dots with error bars. **(C)** Computed APD in the CTL and HF conditions for ventricular and PF models. **(D)** Simulated [Ca^2+^]_i_ in the CTL and HF conditions. Experimental data (O'Rourke et al., 1999) was also shown in the inset.

Figure 2A shows simulated APs in CTL and HF conditions. For ventricles the duration of APs was significantly prolonged in HF with a negligible elevation in the RMP of all three cell types. Figure 2B shows the maximum AP upstroke velocity of ventricular cells were reduced, which matched the experimental records (Hegyi et al., 2019).

Our simulation data show that HF APDs were increased by 25.6% in the Endo, 55.9% in the M and 27.1% in the Epi cell models respectively (Figure 2C), all of which well-matched experimental observations (*see* Supplementary Figure S10A). The regional APD differences as measured in paired M-Epi and M-Endo cells were significantly increased in the HF condition compared to the CTL condition as shown in the Supplementary Figure S10.

The modifications made to intracellular Ca^2+^ regulation in the HF condition resulted in a 60% reduction in the peak [Ca^2+^]_i_ and an increase in the [Ca^2+^]_i_ resting concentration as well as a longer time to Ca^2+^_i_ transient peak and a slowed [Ca^2+^]_i_ decay process as compared to those in the CTL condition (*see* Figure 2D), all of which correlate well with experimental observations (O'Rourke et al., 1999).

We also simulated APD restitution curves with BCL varying from 100 to 2000 ms in the CTL and HF conditions (*see* Supplementary Figure S11). APDs of all three types of ventricular cells were prolonged across the range of examined BCLs in the HF condition. The M cell presented the greatest APD increase in the HF condition among all three cell types.

Supplementary Figure S11 right panel also shows computed ERP in the CTL and HF conditions. In the HF condition, ERPs were dramatically increased in all three cell types. At BCL = 1000 ms, the measured ERP of the M cell model was increased by 174.3 ms, more than twice of that in the Endo (by 77.0 ms) and Epi (by 73.5 ms) cell models, thus indicating increased heterogeneity of ERP in HF.

#### PF Cell Models

HF-induced changes of ionic currents were also incorporated into the PF model. As compared with the CTL condition, the resultant AP presented a slightly lower RMP, a smaller APA and OS, a slower maximum upstroke velocity and a higher PP, all of which matched prior experimental data well, as shown in Supplementary Figure S10 and Supplementary Table S9. Simulation data show that the resultant APD lay within the experimental range in the HF condition (Table 5).

The APD restitution curves (*see* Supplementary Figure S11) show that in the HF condition, there were negligible differences in the APDs between the two conditions. As for ERP, at BCL <800 ms, it was slightly increased in the HF condition and the difference became negligible when BCL reached 800 ms.

### Consideration of Consequences of Individual HF-Remodelled Ionic Currents for Ventricular Action Potentials

Simulations have been done in Endo, M and Epi cells for characterising the functional impact of each individual HF-remodelled ionic current in contribution to APD prolongation. Results obtained from the three cell type models are qualitatively similar. As for illustration, results from the M cell model are shown here for the relative role of each individual HF-remodelled ionic current in contribution to APD prolongation in the ventricular action potentials.

**FIGURE 3:**
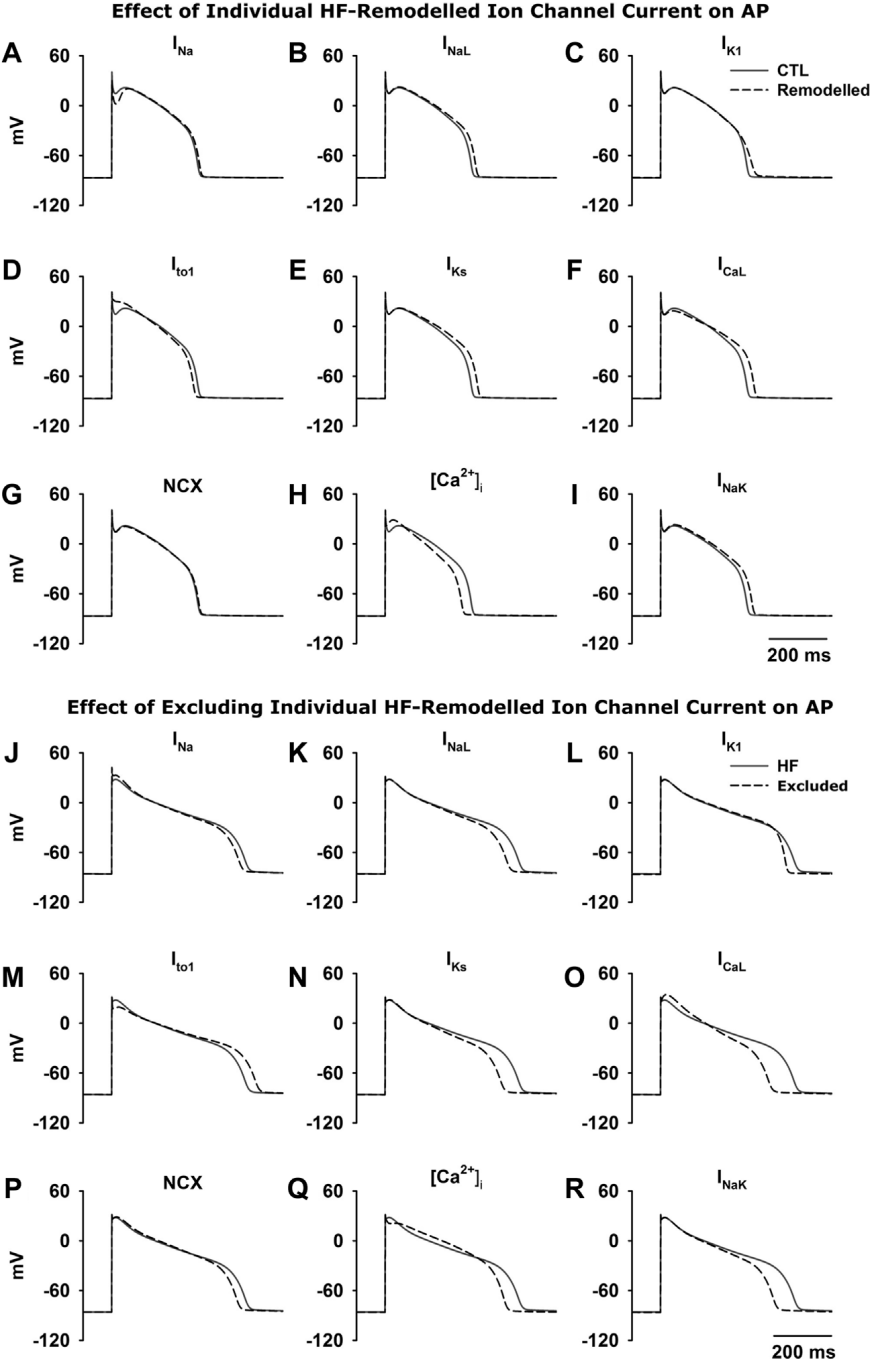
Top **(A**–**I)** Effect of each HF-induced change in ionic current on AP. The APs from the CTL condition were superimposed on those when HF-remodelling on each of the remodelled ionic currents alone was considered. Bottom **(J**–**R)** Effect of excluding each of the HF-induced changes in ionic currents from HF-remodelling on the ventricular APs. The APs in the HF condition (black solid line; all HF-induced changes were considered) was superimposed on those when HF-induced changes in an individual ionic current (black dash line) was excluded.

Figures 3A–I show results when each of the HF-remodelled ionic currents alone was considered. Computed APs were superimposed on those in the CTL condition. The changes in APD and other AP morphological characteristics were summarized in Table 6. A bar chart plot of the APD comparison was also provided in Supplementary Figure S12. Simulation data suggested that each of the HF-remodelled *I*_*NaL*_, *I*_*K1*_, *I*_*Ks*_, *I*_*CaL*_ and *I*_*NaK*_ contributed individually to increased APD (the increase was larger than 10 ms), but the HF-remodelled *I*_*Na*_ or the NCX produced a modest APD prolongation (i.e., <10 ms). In contrast, HF-induced remodelling of *I*_*to1*_ or [Ca^2+^]_i_, abbreviated the APD. The HF-remodelled *I*_*Na*_, *I*_*to1*_ and [Ca^2+^]_i_ also altered other AP characteristics, such as the upstroke velocity and RMP.

**TABLE 6:** Effects of individual HF-remodelled ionic current on the APD of the M cell model.

HF-Induced ionic remodelling	Measured APD when the HF-remodelled ionic current alone was considered (ms)	Relative APD change compared to the CTL condition	AP morphology change compared to the CTL condition
*I*_*Na*_	309.7	+6.7 ms	Decreased phase 0 depolarisation
*I*_*NaL*_	318.2	+15.2 ms	Prolonged APD in phase 3 of AP.
*I*_*K1*_	323.1	+20.1 ms	Prolonged APD in phase 3 of AP.
*I*_*to1*_	287.8	−15.2 ms	The phase 1 repolarisation process was slowed down, causing an elevation in the phase 1 membrane potential and the disappearance of the “spike and dome” feature
*I*_*Ks*_	325.2	+22.2 ms	Prolonged APD in phase 3 of AP.
*I*_*CaL*_	328.2	+25.2 ms	Slightly increased plateau repolarisation
NCX	306.5	+3.5 ms	No significant change
[Ca^2+^]_i_ handling	269.1	−33.9 ms	The phase 1 and phase 2 repolarisation processes were slowed down, causing an elevation in the phase 1 and phase 3 membrane potential. This remodelling boosted the phase 4 repolarisation of the AP significantly
*I*_*NaK*_	321.7	+18.7 ms	Elevated phase 3 membrane potential

### Effects of Excluding the HF-Induced Change in Individual Ionic Currents on Ventricular Action Potential

Simulations were also performed with the HF-induced change in each individual ionic current was in turn excluded (i.e., current properties were adjusted back to the CTL condition) to investigate consequent effects on AP. Results are shown in Figures 3J–R. Computed APs were superimposed on those in the HF condition when all HF-induced changes were considered. Table 7 lists the effect of excluding remodelling of each current on the APD. Corresponding bar charts are shown in Supplementary Figure S12. Simulation data showed that the adjustment of *I*_*Na*_, *I*_*NaL*_, *I*_*K1*_, *I*_*Ks*_, *I*_*CaL*,_ the NCX, [Ca^2+^]_i_ and *I*_*NaK*_ back to the CTL condition individually resulted in a dramatic reduction of the APD by 20–89 ms. However, the adjustment of *I*_*to1*_ back to the CTL condition resulted in a 36 ms increase of the APD.

**TABLE 7:** Effects of excluding each of the individual HF-remodelled ionic currents on the ventricular APD of the M cell.

Excluded currents	Measured APD when each of the HF-Induced changes in ionic currents alone was excluded (ms)	APD change compared to the HF condition	AP morphology change compared to the HF condition
*I*_*Na*_	449.3	−23.2 ms	The OS and APA increased
*I*_*NaL*_	431.0	−41.5 ms	Accelerated phase 4 repolarisation
*I*_*K1*_	439.9	−32.5 ms	Accelerated phase 4 repolarisation
*I*_*to1*_	508.6	+36.2 ms	The phase 1 repolarisation was boosted, causing a reduction in the phase 1 membrane potential
*I*_*Ks*_	410.2	−62.3 ms	Accelerated phase 4 repolarisation
*I*_*CaL*_	383.6	−88.9 ms	The phase 1 and phase 3 repolarisation was slowed, causing a higher phase 1 and phase 3 membrane potentials. And the phase 4 repolarisation was boosted
NCX	441.9	−30.5 ms	Phase 4 repolarisation was speeded up
[Ca^2+^]_i_	420.4	−52.0 ms	The phase 1 membrane potential was reduced while the plateau membrane potential was increased. And the phase 4 repolarisation was boosted
*I*_*NaK*_	400.3	−72.1 ms	Accelerated phase 4 repolarisation

## Tissue Models

### Pro-arrhythmic Effects in the 1D Model

**FIGURE 4:**
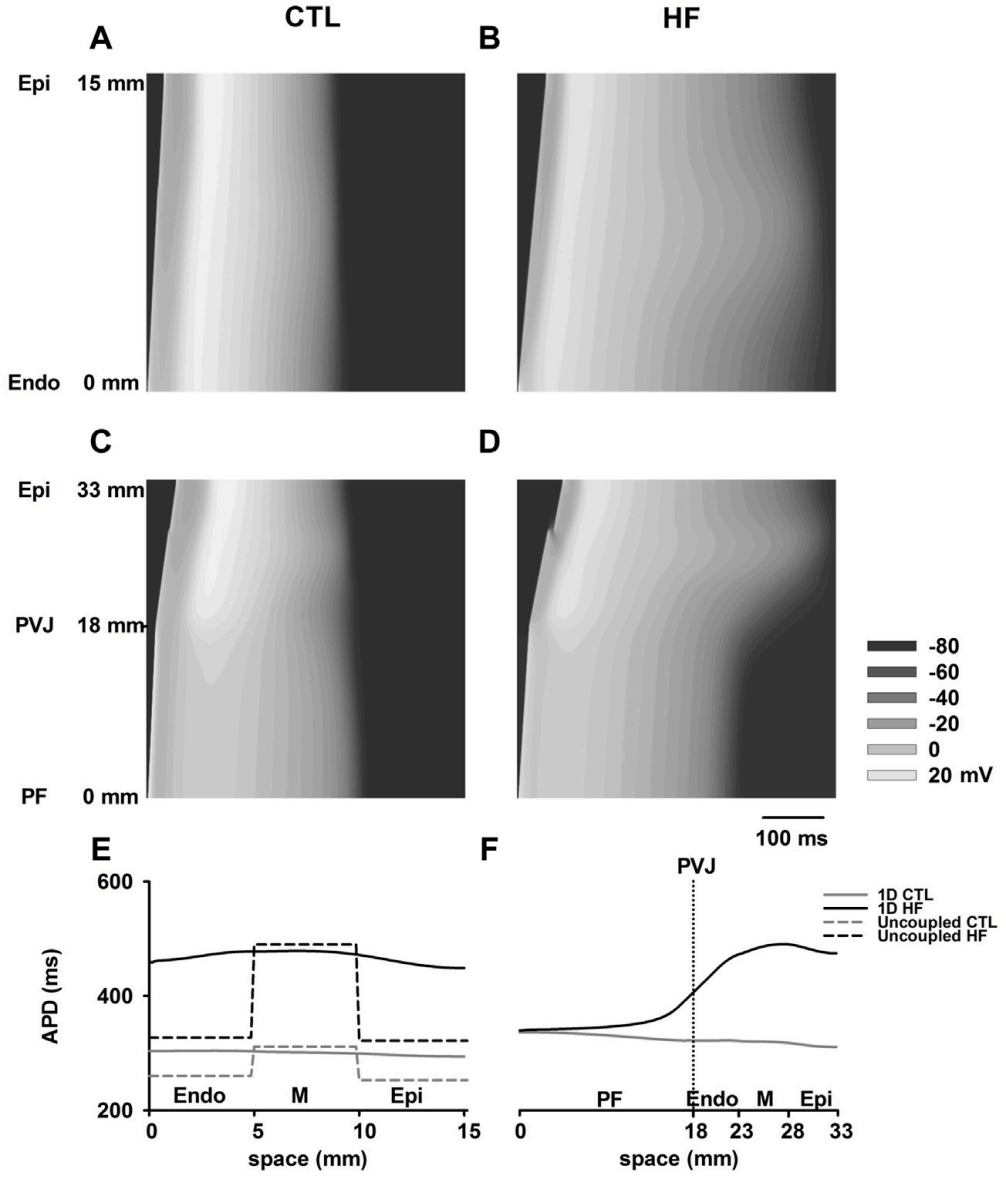
**(A**–**D)** AP propagation along the 1D model of ventricle **(A**,**B)** and PF-ventricle **(C**,**D)** in both CTL and HF conditions. This PF strand had a length of 18 mm and the ventricular strand had a length of 15 mm (Akar and Rosenbaum, 2003; Poelzing and Rosenbaum, 2004) with equal-proportion segments of Endo, M and Epi cells (Yan et al., 1998). In the figures, time ran horizontally, space ran vertically. **(E**,**F)** APD distribution in both CTL and HF conditions for 1D strand models.

Figures 4A–D show simulated AP propagation along two sets of 1D strands in both CTL and HF conditions. Corresponding APD distribution are plotted in Figures 4E,F. Due to electrical coupling, the distribution of ventricular APDs was smoothed compared to the APD differences among isolated cells. The APDs of ENDO and EPI region were increased whereas the midmyocardial APD was abbreviated.

In the 1D model with PF present, the measured APD of PF cells gradually decreased towards ventricles in the CTL condition, whereas it gradually increased towards ventricles in the HF condition. This was due to the marked increase in ventricular APDs in the HF condition. As a result, in contrast to observations with single cell model, the APD of PF in the intact model was actually increased in the HF condition compared to that in CTL condition.

Supplementary Figure S13 examines the AP morphology of the Endo, M, Epi and PF cell models in the 1D model. APs were recorded from the middle of each cell region; the recorded APs were time-shifted such that the APs were superimposed on each other for a direct comparison. In the HF condition, simulated APs from the Endo, M and Epi cell regions of the 1D tissue model showed similar changes to those seen in uncoupled single cells, which included a reduced OS and APA, and a prolonged APD. However, the elevation to the RMP was negligible compared to the CTL condition. On the other hand, the APs of PF cells in the 1D model showed a reduced OS, an increased PP and a slightly elevated RMP, all of which were similar to those of the uncoupled PF cell in the HF condition, whereas the APD was slightly prolonged.

**FIGURE 5:**
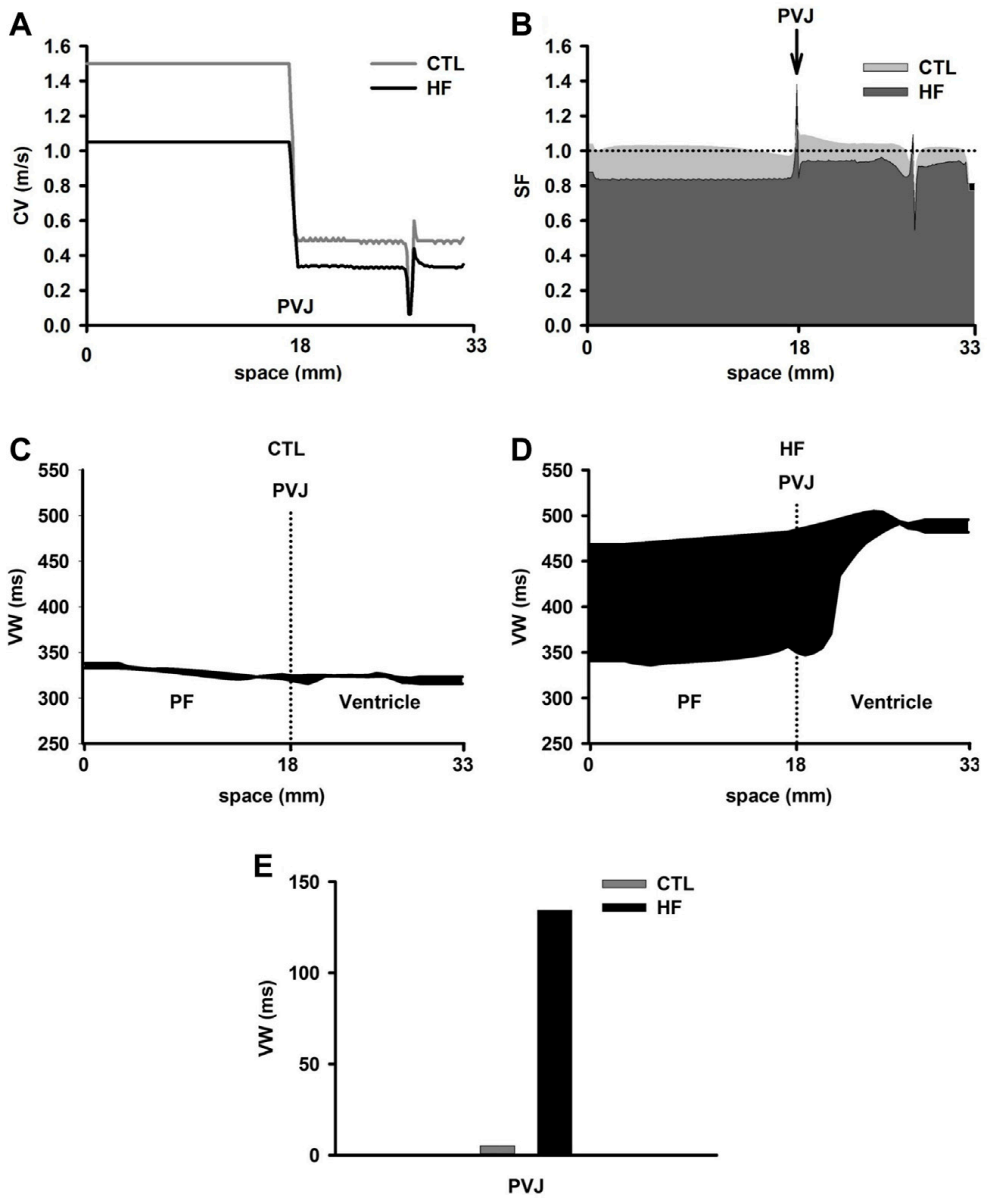
**(A)** Discontinuous CV at the PVJ in both CTL and HF conditions. The electrical excitation wave was conducted from the free-running end of the PF (starting from 0 mm), through the PVJ (18 mm) to the ventricular wall. **(B)** SF computed from the 1D strand model in both CTL and HF conditions. **(C**,**D)** VW computed from the 1D strand in the CTL and HF conditions. **(E)** VW recorded at the PVJ in both CTL and HF conditions.

Figure 5A shows computed CV along the 1D PVJ strand. The resultant CV was decreased by 30% in PF cell regions and 41% in ventricular cell regions in the HF condition. Our simulations suggested a relatively continuous conduction within each of the PF and ventricular cell regions. However, there was a sudden CV drop at the PVJ in both conditions. This CV drop suggested a discontinuous AP conduction at the PVJ (Wiedmann et al., 1996), which may be pro-arrhythmic and likely to cause conduction delay or conduction failure (Gilmour and Watanabe, 1994; Li et al., 1994; Wiedmann et al., 1996; Morley et al., 2005). Note that a CV drop and then recovery was also observed in the ventricle strand at the vicinity of the M-EPI border, which was attributable to a five-fold decrease of the intercellular coupling at the M-EPI border (Gima and Rudy, 2002; Zhang et al., 2008).

To characterise possible pro-arrhythmic effects arising from the conduction discontinuity at the PVJ, we also investigated the conduction SF and VW of the cardiac tissue to unidirectional conduction block.

Figure 5B showed computed SF along the strand with PF in both CTL and HF conditions. Simulations in the CTL condition showed a slight decrease in SF near the boundary between PF and ventricular tissue, followed by a sharp increase of SF at the PVJ.

The reduced CV before the PVJ led to longer time delay, which might enhance the imbalance between *I*_*out*_ and *I*_*in*_ (*see* the Method section), leading to the slight decrease of SF (Aslanidi et al., 2009). At the PVJ, a decrease of the intercellular coupling (i.e., *D*_*PF*_ > *D*_*ventricle*_) reduced electrical loading. As a result, less current leaked into the ventricular tissue at any given moment, and a larger fraction of the charge provided by the ionic current (*I*_*ion*_) was stored by the cell at the junction, which increased SF (Aslanidi et al., 2009).

Similarly, in the HF condition, decreased coupling at PVJ increased SF. However, there was a sharp dip of SF at the PVJ just after the peak site, indicating a likely failure in electrical conduction from the PF cell region to the ventricular cell region. This might be due to the reduced excitability of downstream neighbourhood of cells (Shaw and Rudy, 1997).

Figures 5C–E show computed VW in the 1D strand in both CTL and HF conditions. In the CTL condition, there was a small VW across the 1D strand with no significant differences in the width of the VW between PF and ventricular cells. This suggests an unlikely occurrence of unidirectional block, consequently making it difficult to initiate re-entry. However, in the HF condition, the VW was dramatically increased, especially in the regions of the PF and the PVJ. The difference of the width of the VW between the PF cell region and the ventricular wall was also increased. At the PVJ, the width of the VW was significantly (almost 25 times) greater in the HF condition than that in the CTL condition. These results indicated that there was an increased probability of the occurrence of unidirectional block around the region of the PVJ.

### Investigation of Pro-arrhythmic Effects in 3D Wedge Model

#### Conduction in the 3D Wedge Model

**FIGURE 6:**
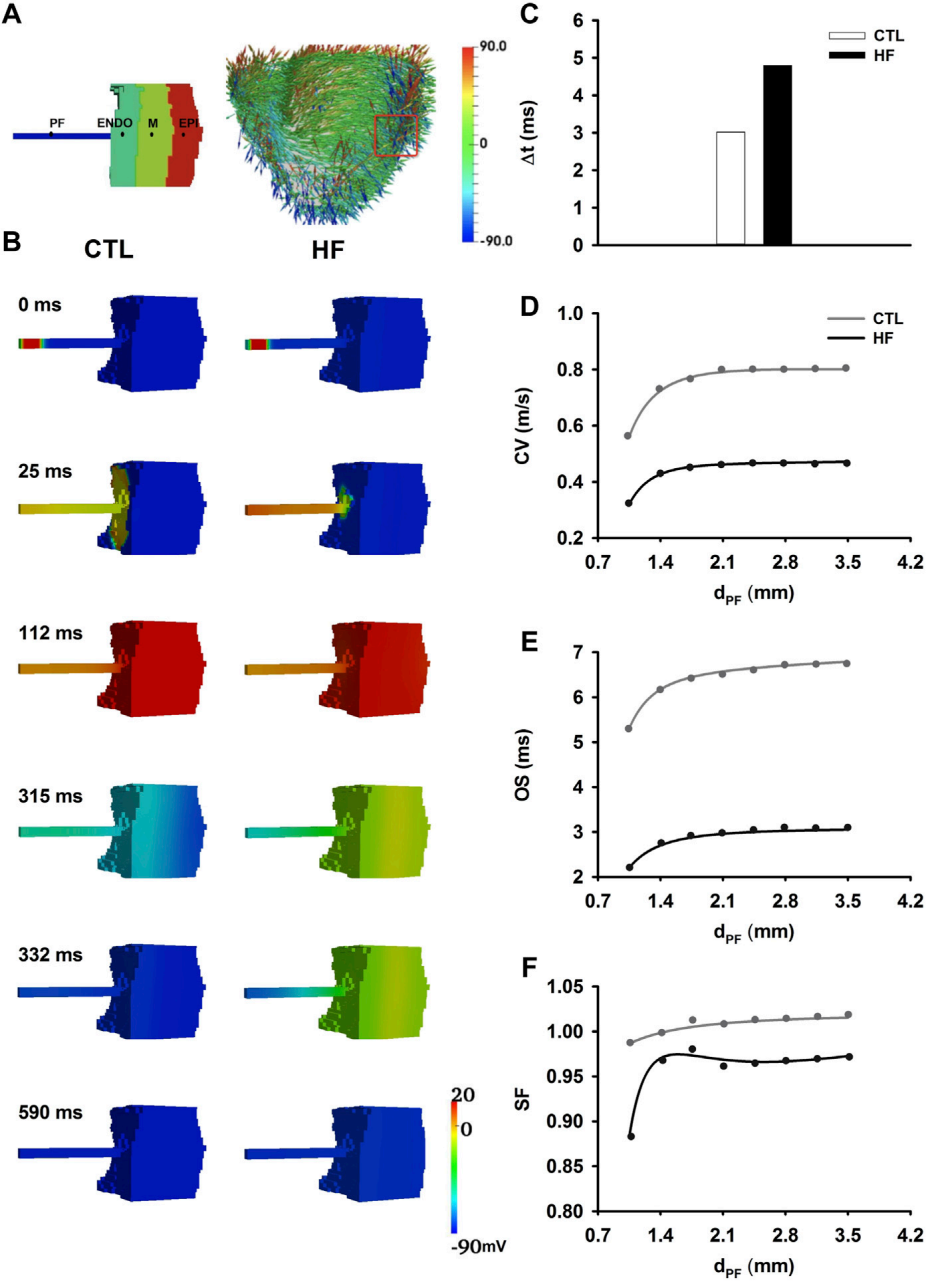
**(A)** Left: Illustration of 3D wedge geometry of PF-ventricle model with *d*_*PF*_ = 2.1 mm. Right: 3D whole canine ventricle model with fiber orientation, from which a 3D slab (marked in red box) was extracted as the 3D wedge model used in this study. **(B)** Electrical wave propagation from the PF, through PVJ to the ventricle in the CTL and HF conditions. **(C)** Time delay (△t) of the conduction through the PVJ in the CTL and HF conditions. △t was recorded from the PF to ventricle approximately 2 mm across the PVJ (Kanter et al., 1993). **(D**–**F)** Relationships between the CV **(D)**, OS **(E)**, SF **(F)** and the width of the PF strand at the PVJ.

Figure 6A showed 3D anatomical model of the ventricle reconstructed from DT-MRI, and the wedge geometry used for the present study. Figure 6B showed electrical wave propagating from PF, through the PVJ to ventricle in the 3D wedge model in both CTL and HF conditions. The excitation wave reached PVJ and successfully propagated in both conditions. Discontinuous conduction at the PVJ resulted in a relatively slow conduction, manifested by a significant time delay in conduction. Simulations showed that the conduction delay was 60% longer in the HF condition than that in the CTL condition (Figure 6C). After the delay at the PVJ, the wave propagated into the ventricle and the ventricle was fully excited.

#### Repolarisation in the 3D Wedge Model

A different sequence of repolarisation between the CTL and HF conditions was observed. In the CTL condition, the sequence of the repolarisation began from the Epi layer in the ventricle to the free-running end of the PF. However, in the HF condition the repolarisation sequence was reversed, and it began from the PF to the ventricle. This difference in the repolarisation sequence was due to the regional APD differences between the PF and ventricular cells, which were altered by HF.

#### Impact of Width of PF on Conduction Safety at PVJ

PF bundles extracted from canine hearts have been found to measure between 0.5 and 2.0 mm in diameter (Reiser and Anderson, 1981; Vassalle and Lee, 1984; Christini et al., 2006). In order to investigate the impact of variation in width of PF bundles on conduction safety, CV (Figure 6D), OS (Figure 6E) and SF (Figure 6F) at the PVJ were computed with varied widths (*d*_*PF*_) of PFs. In simulations, eight values of *d*_*PF*_ were considered, which changed from 1.05 to 3.5 mm at a step of 0.35 mm. The changes of CV, OS and SF along with increased *d*_*PF*_ showed similar gradients in both CTL and HF conditions. However, the values in the HF condition were much smaller.

**FIGURE 7:**
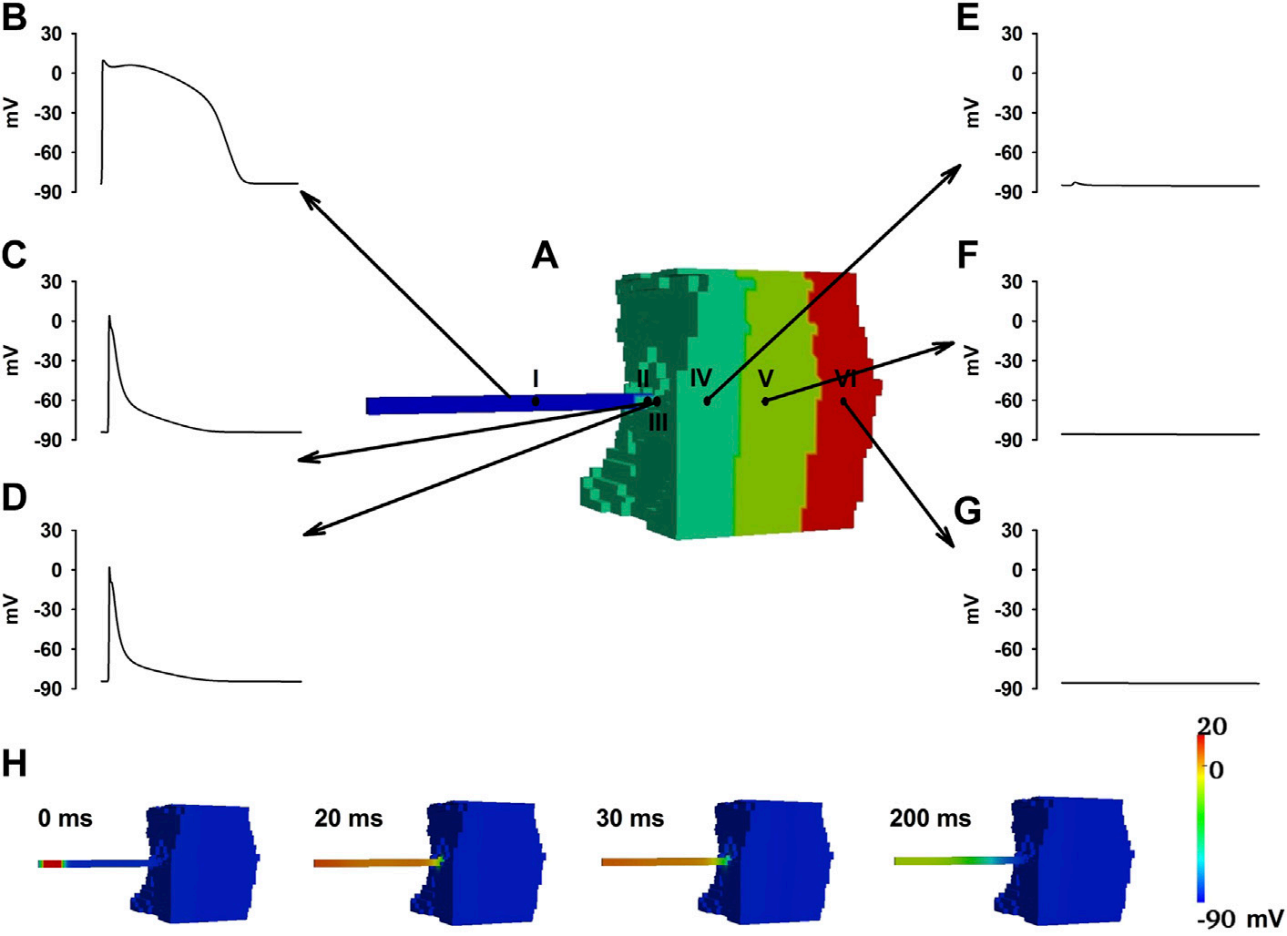
Conduction failure at the PVJ with the PF width of 1.4 mm in the HF condition. **(A)** Model geometry with the selected points indicated as dots. Point I was at the middle of the PF. Point IV was at the middle of the Endo layer. Point V was at the middle of M layer. Point VI was at the middle of Epi layer. Points II and III represented the PF and Endo cell within the PVJ. **(B**–**F)** APs recorded from selected sites. **(H)** Demonstration of wave propagation failure at PVJ with the PF width of 1.4 mm in the HF condition.

In the HF condition, *d*_*PF*_ = 1.4 mm was considered to be a “critical width.” Below the “critical width,” the values of CV, OS and SF at PVJ dropped quickly. In the CTL condition, conduction succeeded with all ranges of *d*_*PF*_ in our simulations. In the HF condition conduction failed at the PVJ when *d*_*PF*_ equalled the “critical width” (Figure 7). Lowered OS represented the reduced driving power to excite the downstream neighbourhood cells, provoking an AP. On the other hand, thinner PF led to heavier source-to-load mismatch from PF to ventricular tissues. As a result, conduction failed at the PVJ. As shown in Figure 7, where APs were recorded from several sites in the tissue model as marked in Figure 7A, the PF cell (point I) along the strand was able to complete a normal AP course (Figure 7B) but cells at the PVJ (point II and III) were only able to initiate an abnormal AP with a dramatically small OS, rapid repolarisation process and short APD (Figures 7C,D), all of which resulted in insufficient diffusion current to their downstream neighbours to fire an AP. Therefore, cells in the Endo (point IV, Figure 7E), M (point V, Figure 7F) and Epi (point VI, Figure 7G) layers were not able to generate an AP, leading to conduction failure as shown in Figure 7H.

In our study, the electrical wave failed to trigger an action potential in the ventricle in when *d*_*PF*_ was less than 2 mm in the HF condition. In this condition, when *d*_*PF*_ reached 2mm, the electrical wave was able to propagate successfully through the PVJ. This successful conduction at the PVJ was due to the relatively large CV and OS compared to those when *d*_*PF*_ = 1.4 mm.

#### Functional Re-entry in the 3D Wedge Model

For this part of the study, we used the wedge model with dual idealised PF strands: one with a relatively thicker width (*d*_*PF1*_ = 2.8 mm) and the other with a relatively thinner width (*d*_*PF2*_ = 1.4 mm, the “critical width” in the HF condition). The value we used for the thicker PF strand width is higher than but close to the upper bound of the experimental data range (i.e., 2 mm). This value was used to ensure a smooth conduction at the PVJ *via* the thicker PF bundle in the HF condition.

**FIGURE 8:**
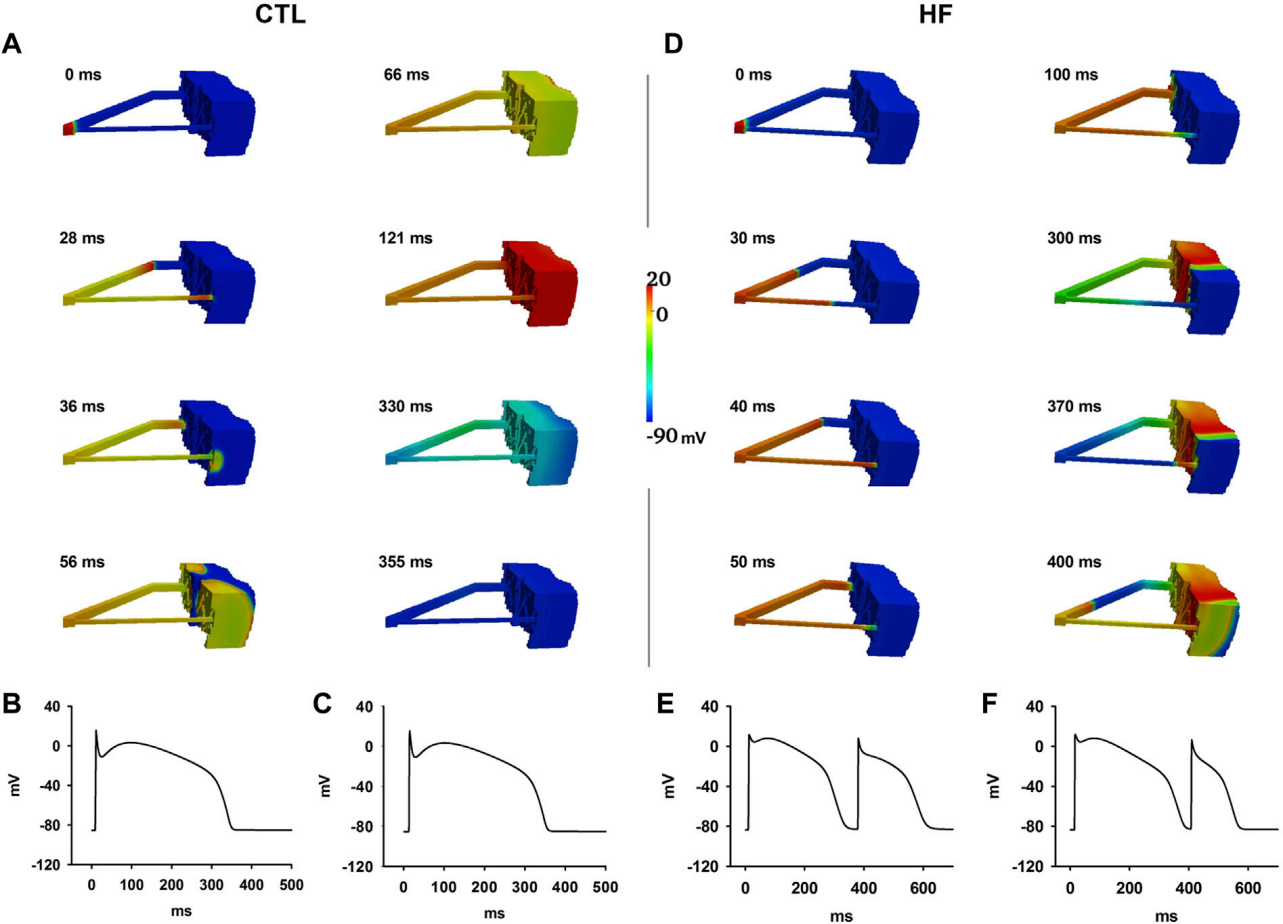
**(A**–**C)** Excitation wave propagation in the CTL condition. APs at the middle of the thin **(B)** and thick **(C)** PF regions were recorded. **(D**–**F)** Excitation wave propagation in the HF condition. APs at the middle of the thin **(E)** and thick **(F)** PF regions were recorded.

Figure 8A shows wave propagation in the CTL condition. Once initiated, the evoked excitation wave conducted rapidly along both PF branches, propagated through both PVJ successfully and caused the ventricle to excite. The repolarisation sequence began in the Epi layer first and gradually moved towards the PF. Recorded APs of PFs from middle positions of the thin (*see* Figure 8B) and thick (*see* Figure 8C) PF regions demonstrated one single wave conduction along dual pathways of the PF network in the CTL condition.

With the same simulation settings, functional re-entry was generated in the HF condition (Figures 8D–F). The wave successfully conducted through the PVJ *via* the thick PF to excite the ventricle but it failed to propagate *via* the PVJ of the thin PF. The wave front from the thick PF propagated slowly in the ventricle towards the PVJ of the thin PF. When it reached the PVJ of the thin PF, the previous AP at the thin PF already repolarised. Then the wave front re-entered the thin PF *via* the PVJ; thus functional re-entry occurred. Recorded APs of PFs from middle positions of the thin (*see* Figure 8E) and thick (*see* Figure 8F) PF regions demonstrated two wave conductions along dual pathways of the PF network in the HF condition.

## Discussion

The major findings of this study are: *1*) for ventricular cells, the HF-induced remodelling in ion channel, transporter and Ca^2+^-handling prolonged cellular APD and ERP. It also reduced the MUV, OS, cellular excitability, amplitude of the intracellular Ca^2+^ transient, which all correspond with experimental data (O'Rourke et al., 1999; Maguy et al., 2009; Kaab et al., 1996; Undrovinas et al., 1999). These simulation data imply that the experimentally identified electrical and Ca^2+^-handling remodeling is sufficient to account for the observed changes in cellular action potentials (*see* Table 2). For the PF cells, however, the HF-induced ionic remodelling had negligible effects in terms of prolonged APD and ERP, though the changes in other characteristics of APs (MUV, OS, cellular excitability, amplitude of the intracellular Ca^2+^ transient) are similar to those observed in ventricular cells. These simulation data are also in agreement with prior experimental data (Supplementary Table S9). As such, HF augmented the regional AP difference between ventricular and PF cells, which is potentially of pro-arrhythmic; *2*) in the 1D model, HF-remodelling reduced the conduction velocity of excitation waves in PF and ventricular tissues, whilst augmenting the APD dispersion in the PF strand, PVJ and the transmural ventricle strand. It also increased the source-sink mismatch at the PVJ as manifested by the increased time delay for the conduction of excitation waves from PF to the ventricle, leading to a decreased conduction SF at the PVJ, but an increased tissue vulnerability for the genesis of uni-directional conduction block in response to a premature S2 stimulus; *3*) in the 3D wedge model, HF-remodelling altered the repolarization sequence of intact PF-ventricle. In the CTL condition, PF repolarised later than ventricles, preventing the excitation wave from re-entering PF from ventricular wall. However, in the HF condition, the PF cells repolarised earlier than ventricles, providing a “substrate” for the excitation wave to re-enter the PF from the ventricle wall that led to the genesis of re-entrant excitation waves at PVJ; and *4*) most importantly, HF-remodelling reduced the ability of the PF for driving the ventricle, leading to an increased “critical width” of the PF bundles to ensure successful electrical conduction at the PVJ. Consequently, conduction failed at PVJs where the PF strands were thin, forming a substrate that allowed excitation waves to re-enter the PF network from the ventricle and thus the formation of re-entrant excitation waves. Collectively, these simulation results provide novel insights into understanding the proarrhythmic effects of HF.

### Functional Role of Individual ion Current and Ca^2+^-Handling Remodelling in Modulation to the APD and ERP in Ventricular and PF Cells

It is well established that HF can prolong the APD of ventricular cells in different species, including canine (Kaab et al., 1996; Undrovinas et al., 1999), human (Beuckelmann et al., 1992) and rabbit (Rose et al., 2005). Experiments have also shown that HF abbreviated the APD of PF cells (Li et al., 1993; Han et al., 2001; Maguy et al., 2009). Our simulation data at the single cell level also showed prolongation and abbreviation of ventricular and PF APDs respectively. This is in agreement with prior experimental findings, implying that the experimentally observed ion current and Ca^2+^-handling remodelling were sufficient to account for the APD changes in ventricle and PF cells as observed experimentally.

The simulated ventricular APD prolongation can be accounted for by the combined action of all ion current and Ca^2+^-handling remodelling. However, the relative roles of each of the remodelled ionic currents in contributing to APD prolongation varied. Our simulation results suggested that for HF-remodelled *I*_*NaL*_, *I*_*K1*_, *I*_*Ks*_, *I*_*CaL*_ and *I*_*NaK*_, each of them contributed a marked increase in the APD prolongation. For *I*_*NaL*_, HF increased its current density but slowed down its inactivation time course, which led to an increased current amplitude during the time course of AP, thereby contributing to plateau prolongation. HF decreased the current density of *I*_*K1,*_ *I*_*Ks*_ and *I*_*NaK*_, which led to a slowing of repolarization, thereby also contributing to AP prolongation. For *I*_*CaL*_, HF shifted its steady state activation curve leftward that resulted in an increased window current (Beetz et al., 2009), leading to an elevated membrane potential in phase 3 and a prolonged APD. However, the effect of the HF-induced decrease in *I*_*to1*_ contributed to a reduced APD, which initially seemed counter-intuitive, given that a reduced outward K^+^ channel current would normally increase the APD. We further analysed our simulation data and found that this was due to an interplay between ionic currents. A reduction in *I*_*to*_ increased the plateau membrane potential, leading to an increased activation of the delayed rectifier potassium channel currents that promoted repolarization, which is consistent with a previous observation (Grant, 2009). However, the integrated effects of HF on the different ionic currents outweigh such an effect, with the net consequence of APD prolongation.

It has been noted that HF had different effects in modulating APs in ventricle and PF cells, which might be attributable to the differences in the HF-induced remodelling on ionic properties between the two cell types (Li et al., 1993; Han et al., 2001; Maguy et al., 2009). Our simulation results supported this notion. In the HF condition, *I*_*Na*_ *, I*_*K1*_ *, I*_*to1*_ *, I*_*Ks*_ *, I*_*CaL*_ and [Ca^2+^]_i_ were reduced in both PF and ventricular cells, whilst *I*_*NaL*_ and the NCX were increased and *I*_*NaK*_ was reduced in ventricular cells; none of them was changed in the PF cell. On the other hand, *I*_*Kr*_ was reduced in the PF cell but not in ventricular cells (Li et al., 2002); additionally, in PF there was a reduction of *I*_*CaT*_, which was not present in canine ventricular cell models. Those differences in the HF-induced remodelling on K^+^ currents (outward current) and Ca^2+^ currents (inward current) along with an increased *I*_*NaL*_ (inward current) in ventricular cells can explain the different modulation of HF on cellular APs between the PF (shortened AP) and ventricular (prolonged AP) cells.

### Mechanistic Insights Into the Proarrhythmic Effects of HF

Experimentally it has been shown that HF is associated with abnormal electrical wave conduction in the intact PF-ventricular tissue, which provides a pro-arrhythmic substrate that favours increased vulnerability to and sustainability of arrhythmias in the HF condition (Peters et al., 1997; van Rijen et al., 2004; Cabo et al., 2006). Abnormal conduction manifested as a reduced electrical wave conduction velocity in the PF and ventricular tissues, but an increased conduction time delay at the PVJ. In ventricular tissue, about ∼40% reduction in the conduction velocity has been observed (Akar et al., 2007; Maguy et al., 2009), which may be attributable to reduced Cx43 proteins (Peters et al., 1997; van Rijen et al., 2004; Cabo et al., 2006). Our simulation data provide evidence to support this proposition. It has been shown that both HF-induced remodelling in ionic properties and the intercellular coupling contributed to the CV reduction, which was by about 40% in the ventricular and 30% in the PF tissue models. Such CV reduction was attributable to the integral action of reduced maximal upstroke velocity of AP arising from the ionic remodelling and the intercellular coupling arising from the reduction of Cx43. The simulated reductions in CV by HF in the tissue models are numerically in agreement with experimental observation (Akar et al., 2007; Maguy et al., 2009). Due to the reduced CV, the excitation wavelength was reduced, which facilitated the formation and maintenance of re-entrant excitation waves in a limited, pro-arrhythmic cardiac tissue substrate.

A conduction time delay at the PVJ arising from the source-sink mismatch provides a potential cause for the genesis of unsafe conduction and/or even conduction failure that leads to formation of re-entrant arrhythmia (Gilmour and Watanabe, 1994; Li et al., 1994; Wiedmann et al., 1996; Morley et al., 2005). In this study, we have shown that HF increased markedly such a conduction delay and consequently vulnerability to the genesis of conduction block at the PVJ was augmented. Due to the integral action of a reduced maximal upstroke velocity and intercellular electrical coupling in the HF condition, the driving force for the PF to excite the ventricular tissue was reduced, leading to a sharp decrease of conduction SF at PVJ, although the electronic load of the ventricular tissue to the PF was reduced due to the reduced intercellular coupling. Therefore, in the HF condition, there was less current flowing into downstream neighbour cells than the current received from upstream PF cells, leading to an increased likelihood of occurrence of conduction failure, which would require an increased thickness of PF strands to overcome the source-sink mismatch in ensuring the PF to successfully drive the ventricle as observed by Aslanidi et al. (2009). At the PVJs where the PF strands are thin, the reduced SF may predispose to conduction failure that leads to formation of re-entry.

### Pro-arrhythmic Role of Altered Repolarisation Dispersion and Conduction Discontinuity at PVJ

Reduced transmural dispersion of repolarisation in ventricles in the HF condition has been observed experimentally and has been believed to underlie the electrophysiological mechanism for the genesis of unidirectional block, formation of re-entry and arrhythmogenicity (Li et al., 2002; Bai et al., 2006; Wang and Hill, 2010). In this study, such reduced ventricular transmural dispersion was also seen in our simulations, which is consistent with experimental observations (Zhu et al., 2009; Osadchii, 2017). However, it is difficult to causally link the reduced ventricular repolarisation dispersion to the increased pro-arrhythmogenesis in the HF condition as the reduced dispersion might be expected to be anti-arrhythmic. However, the paradoxical effects of the reduced dispersion of repolarisation can be explained by the altered repolarisation dispersion sequence between PF and the ventricle junctions, which, to our best knowledge, has not been investigated previously. In the control condition, the repolarisation of PF strand was later than that of the adjacent ventricle tissue, preventing the re-entry of excitation waves from ventricle tissue to the PF at the junction, even at points where the PF failed to drive the ventricular tissue. However, in the HF condition, the repolarisation sequence between PF and ventricles was reversed, causing the repolarisation of PF to be earlier than that of the adjacent ventricular tissue due to the different modulatory role of HF on the APDs of the ventricle and PF cells (Figure 2). At the PVJs where PF are thin, PFs failed to excite the adjacent ventricular tissue due to the augmented source-sink mismatch and the reduced excitability of ventricular cells in the HF condition. However, at the PVJs where PF are thick enough, the conduction wave propagated into ventricle with a slow conduction velocity and long APD. When the excitation wave reached the PVJ sites where the conduction from PF to ventricle failed at the first instance, the ventricular APs with a longer ventricular APD or later completion of the repolarisation process provided a stimulus trigger to evoke a second excitation in the PF, opening a “gate” allowing the conduction wave to re-enter from the ventricle back to the thinner PF. Consequently the ventricular action potentials re-entered the thinner PF bundle from the ventricle and triggered the genesis of re-entrant wave at the PVJ as seen in Figure 8.

Altered repolarisation dispersion at the PVJ by pathological conditions has been observed in sheep and ovine models (Walton et al., 2014; Martinez et al., 2018), as well as in experimental and computational murine models of CPVT (Blackwell et al., 2022). It is clear from the studies of repolarization heterogeneity that the Purkinje fiber ventricular junction influences dispersion of repolarization. Moreover, in the study of Blackwell et al. (2022), which was a combined experimental and simulation investigation, the Purkinje–myocardial junction was seen to be involved as the anatomic origin of ventricular arrhythmia in the murine CPVT model. The ablation of subendocardial cells adjacent to PF was sufficient to protect against catecholamine induced arrhythmias and, in simulations, DADs in ventricular myocardium could trigger action potentials in PFs, but not *vice versa*.

All together this suggests that the impact of HF on the conduction discontinuity and dispersion of repolarisation at the PVJ plays an important role for the initiation of re-entry, adding new understanding of the pro-arrhythmic mechanisms of HF.

### Relevance to Previous Studies

Several other studies have developed models to simulate the effects of HF-remodelling on modulating ventricular APDs (e.g. Priebe and Beuckelmann, 1998; Flaim et al., 2006; Trenor et al., 2012), most of which concentrated on a few HF-remodelled ion currents. The ventricular and PF models employed in the present study took into consideration comprehensive HF-induced ion channel, Ca^2+^ handling and intercellular electrical coupling remodelling, based on available information, allowing the reproduction of major changes in electrophysiology observed experimentally.

In this study, we have shown that HF augmented discontinuous conduction at the PVJ due to the source-sink mismatch. This may cause conduction failure at some PVJ sites where the PFs were not able to drive the ventricle. As such, a “gate” is produced allowing a previous excitation wave to retrogradely re-enter the PF network, forming a re-entrant pathway. Our observations support the “gate hypothesis” proposed by Myerberg et al. (Myerburg et al., 1970; Myerburg et al., 1971), which suggested that the area with the maximum APD in PF determines the minimal AP coupling intervals for successful conduction at the PVJ, thus preventing premature action potentials to propagate into the ventricle (Myerburg et al., 1971). Based on this hypothesis, Lazzara et al. (Lazzara et al., 1976) subsequently proposed a theory to explain the formation of PF-ventricle re-entry, that in some conditions in which conduction to ventricle succeeded in one PF branch but failed in another, ventricular conduction might retrogradely propagate through the gate previously in refractory period and form a re-entrant wave (Lazzara et al., 1976). Experimental observations conducted by Logantha et al. (Logantha et al., 2021) provided data to support this theory.

This study provides insights into the mechanisms underlying the “gate hypothesis.”

We have shown that the formation of PF-ventricle re-entry in the HF condition is associated with two major factors: one is the impaired driving power of the PF to the ventricle and the other is the reduced APD dispersion at the PVJ, rather than a large APD dispersion as foreseen by the “gate hypothesis.” Due to the weakened driving power, conduction might fail at PVJs with the width of PF below a critical value. Due to the reduced APD dispersion at the PVJs by HF, PF repolarised earlier than the ventricle, allowing ventricular excitation retrogradely propagating into the PF. In addition, slow conduction in the HF condition also reduced the excitation wavelength, facilitating the initiation and maintenance of re-entrant waves.

We incorporated investigation of the effects of HF-induced electrical and gap junctional coupling remodelling on the discontinuous conduction at the PVJ. Our results have shown that in the HF condition, the critical width of PF to drive the ventricle was significantly increased, attributable to the reduced intercellular coupling and the amplitude of cellular action potentials. Our results are consistent with a previous computational study of Behradfar et al. (Behradfar et al., 2014), who investigated the impact of electrical resistance (equivalent to the diffusion coefficient in this study) and size of PVJs on arrythmogenesis at the PVJ. Their data showed that when the resistance was high (i.e., low D in our study), there wasn’t enough charge passed from PF to downstream neighbouring ventricle to fire an action potential. When the resistance was too low, it suppressed prejunctional action potentials, also leading to conduction failure. Our results of the conduction failure at the PVJ in the HF condition when the diffusion coefficient was reduced match the findings of Behradfar et al. (Behradfar et al., 2014).

In this study, from the PF strand to the PVJ and Epi end of the 1D PF-ventricle model, a gradual decrease (i.e. downward gradient) in APD was shown in CTL condition, but it was changed to an upward gradient in HF due to a dramatic APD increase in the ventricle (*see* Figure 4). These simulation data differ from those shown in (Li et al., 2015), for which rabbit PF and ventricle cell models were used: in that study a downward gradient in APD the PF to the ventricular muscle in the CTL condition was enhanced in HF condition. This difference may be due to the species difference between rabbit and canine, where a significant APD increase in the PF cell was observed in the HF condition; however, there is little APD change in the HF condition as compared to CTL seen in simulations and experimentations (Li et al., 2015).

### Potential Clinical Implications

HF is associated with high risk of cardiac arrythmias causing morbidity and mortality (Dean and Lab, 1989; Aistrup et al., 2011; Masarone et al., 2017). However, incomplete understanding of the mechanisms underlying the pro-arrhythmic effects of HF impedes effective prevention and treatment of cardiac arrhythmias in this condition. In this study, we adopted a computational modelling approach to analyse the integrated and individual roles of HF-induced changes in the electrical properties and kinetics of the ion channels and transporters in modulating cellular action potentials of PF and ventricle cells, the regional repolarization dispersion and discontinues electrical conduction at the PVJ. The identified major role of HF-remodelled ion channels (including the augmented I_NaL_ and decreased I_K1_) in this may provide possible novel target(s) in antiarrhythmic therapy. In addition, we have found that the discontinuous conduction at the PVJ forms a pro-arrhythmic substrate in the HF condition, due to the altered dispersion of repolarisation and increased source-sink mismatch. Further experimental and clinical studies are warranted to analyse possible effects of targeting these features on generation of re-entry at the PVJ, in order to evaluate whether they constitute valuable approaches for anti-arrhythmia strategy in HF conditions.

### Limitations

#### Single Cell Modelling

Limitations of the Benson *et al.* (2008) and Aslanidi *et al.* (2009) models were comprehensively described by Benson *et al.* (2008) and Aslanidi *et al.* (2009). In the development of canine ventricular HF models, all modifications were based on canine experimental data except *I*_*CaL*_ and NCX, which were based on human and rabbit data respectively. The modification to *I*_*NaK*_ was based on HF-induced changes at the protein level as there were no experimental data available for its current density. Changes to the intracellular Ca^2+^ regulation were empirical; parameters related to the SERCA uptake, SR leak and release were adjusted to ensure that the simulated [Ca^2+^]_i_ matched experimental observations. These limitations should be addressed when more experimental data become available. In addition, the experimental data on ion channel remodelling implemented in this study do not take into account the possibility that HF related remodelling may change with time, and thus the extent to which ionic currents/channels remodel (for example, I_Kr_ remodelling) or not may depend on the duration of the HF condition (Long et al., 2015). The effects of chronic HF remodelling on ventricular excitation warrant further study in the future.

In the development of the PF HF model, *I*_*Na*_ was modified based on AP upstroke characteristics as well as the consideration of the down-regulated Na_v_1.5. The intracellular Ca^2+^ regulation was modified with the assumption that the HF-induced remodelling of that in ventricular cells was applied to the PF cell.

#### Limitations of Tissue Level Modelling

The 3D wedge model was developed in the case of axially symmetric anisotropy, in which CVs in both transverse directions (orthogonally to fibers) were assumed to be the same. HF-induced remodelling on the CV along fibers and orthogonally to fibers were assumed to be homogenous, both of which were reduced by ∼41% in the ventricle. Due to the limited 3D wedge geometry data, the 3D geometry with dual idealised PFs was constructed by using two identical 3D geometries with the same sheet structure and fiber orientation. Further model development is warranted to consider 3D anatomical models in both CTL and HF conditions.

In the 1D model, the intercellular coupling strength (reflected by the diffusion parameter D in the model equation) was chosen to obtain the conduction velocity in the PF and ventricular strands, each of which matched the respective experimental data. However, we have not found in the literature, data about the coupling at the PVJ. To avoid a discontinuous “jump” in the diffusion coefficient in simulations, we implemented linear interpolation to smooth the conduction at the PVJ. As the coupling at the PVJ is important for conducting the excitation wave from the PF to the ventricle, this warrants careful investigation in future when more experimental data become available regarding the electrical coupling at the PVJ.

The two PF strands in the 3D wedge model are idealized, not from the imaging datasets as these do not include the free running PF network. In the model, the two PF strands were coupled to the myocardium at two insertion points located within the small endo-surface of the wedge. The distance between the two insertion points may not reflect the real distance between two realistic adjacent PF strands. However, it may represent the case in which among a set of PF insertion points, there are a pair of them at a sufficiently large distance as has been modelled in this study.

Another potential limitation is that the present study implemented two cellular models, rather than population models that can account for biological variability (Britton et al., 2013). In addition, the two models were based on the canine data, which may be different to the data of humans due to species differences (Bers, 1991). When equivalent detailed experimental data become available from human, models of human PF-ventricles implementing human ventricular and PF cell models (Trovato et al., 2020; Stewart et al., 2009; O'Hara et al., 2011; Tomek et al., 2019) warrant to be used to investigate the pro-arrhythmic role of HF at PVJ.

Despite these potential limitations, the data we present do provide insights into pro-arrhythmia mechanisms of HF due to the discontinuous conduction at the PVJ.

## Conclusion

In this simulation study HF-induced remodelling of ion channels, Ca^2+^ handling and the intercellular electrical coupling reduced the excitability of cells and impaired PF and ventricular conduction. HF augmented the source-to-sink mismatch, resulting in a reduced SF but an augmented vulnerability to the genesis of unidirectional conduction block at the PVJ. In the HF condition, greatly increased APD in ventricle but not in the PF altered the transmural dispersion of repolarisation sequence within the ventricle and at the PVJs, allowing excitation waves to re-enter the PF network from the ventricles. In addition, slowed conduction in PF and ventricular tissue helped to sustain the re-entrant arrhythmic waves. Our study illustrates the important role of altered repolarisation dispersion at PVJs in initiating re-entry and, in doing so, highlights the contribution of altered PVJ electrophysiology in arrhythmogenesis of HF.

## Data Availability

The original contributions presented in the study are included in the article/Supplementary Material, further inquiries can be directed to the corresponding author.
