# Supplementary material for: Pro-Arrhythmic Effects of Discontinuous Conduction at the Purkinje Fiber-Ventricle Junction Arising From Heart Failure-Induced Ionic Remodeling – Insights From Computational Modelling
Source: Front Physiol. 2022 Apr 25;13:877428. doi: 10.3389/fphys.2022.877428 (PMC9081695; doi:10.3389/fphys.2022.877428)
Supplement: Supplementary file 1 [file Image12.pdf]

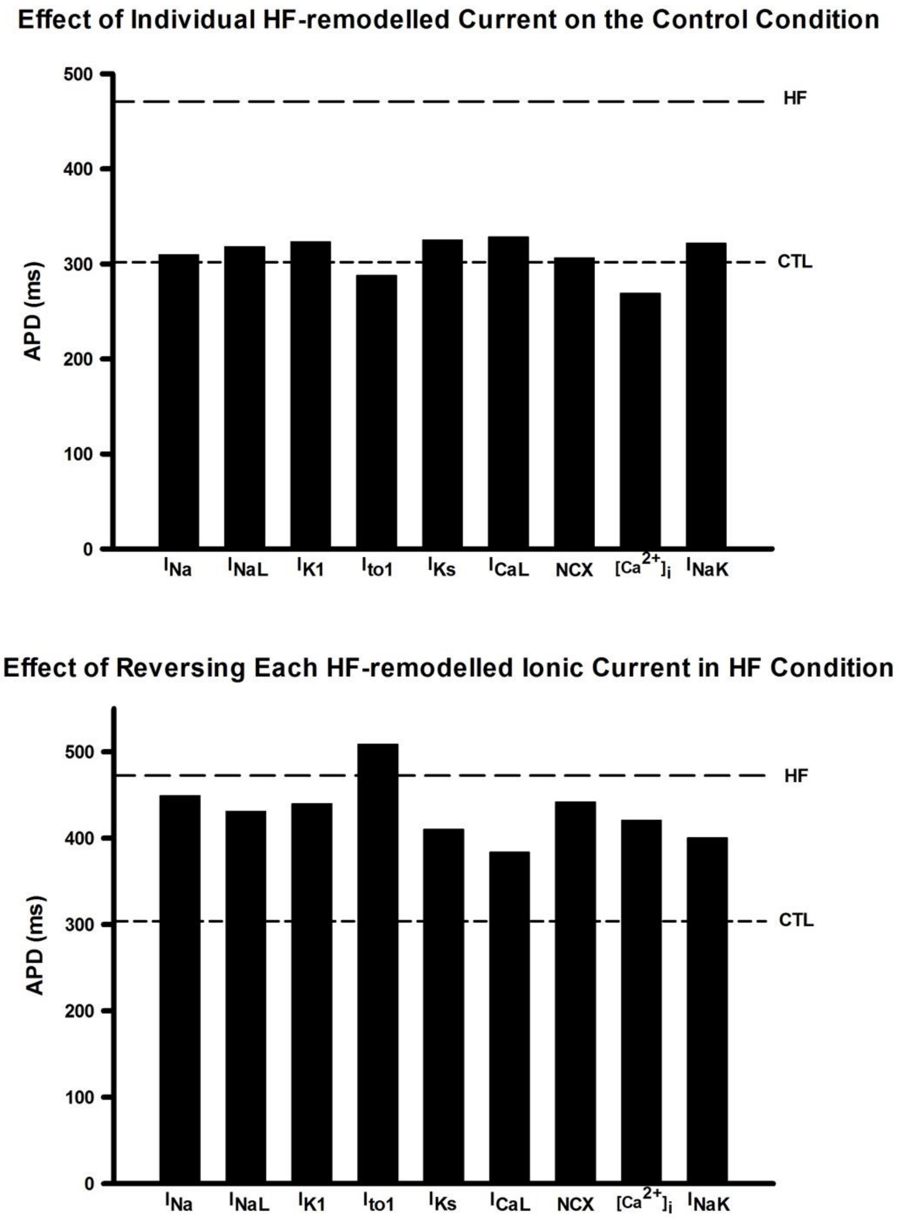

Supplementary Figure S12 Effect of individual current on APD. Top: Computed APD from the M cell model when the changes on each of the HF-remodelled ionic channels were considered alone (BCL = 1000 ms). Bottom: Measured APD when each of the individual HF-remodelled ionic channels was adjusted back to the CTL condition.
