# Supplementary material for: Pro-Arrhythmic Effects of Discontinuous Conduction at the Purkinje Fiber-Ventricle Junction Arising From Heart Failure-Induced Ionic Remodeling – Insights From Computational Modelling
Source: Front Physiol. 2022 Apr 25;13:877428. doi: 10.3389/fphys.2022.877428 (PMC9081695; doi:10.3389/fphys.2022.877428)
Supplement: Supplementary file 2 [file Image5.pdf]

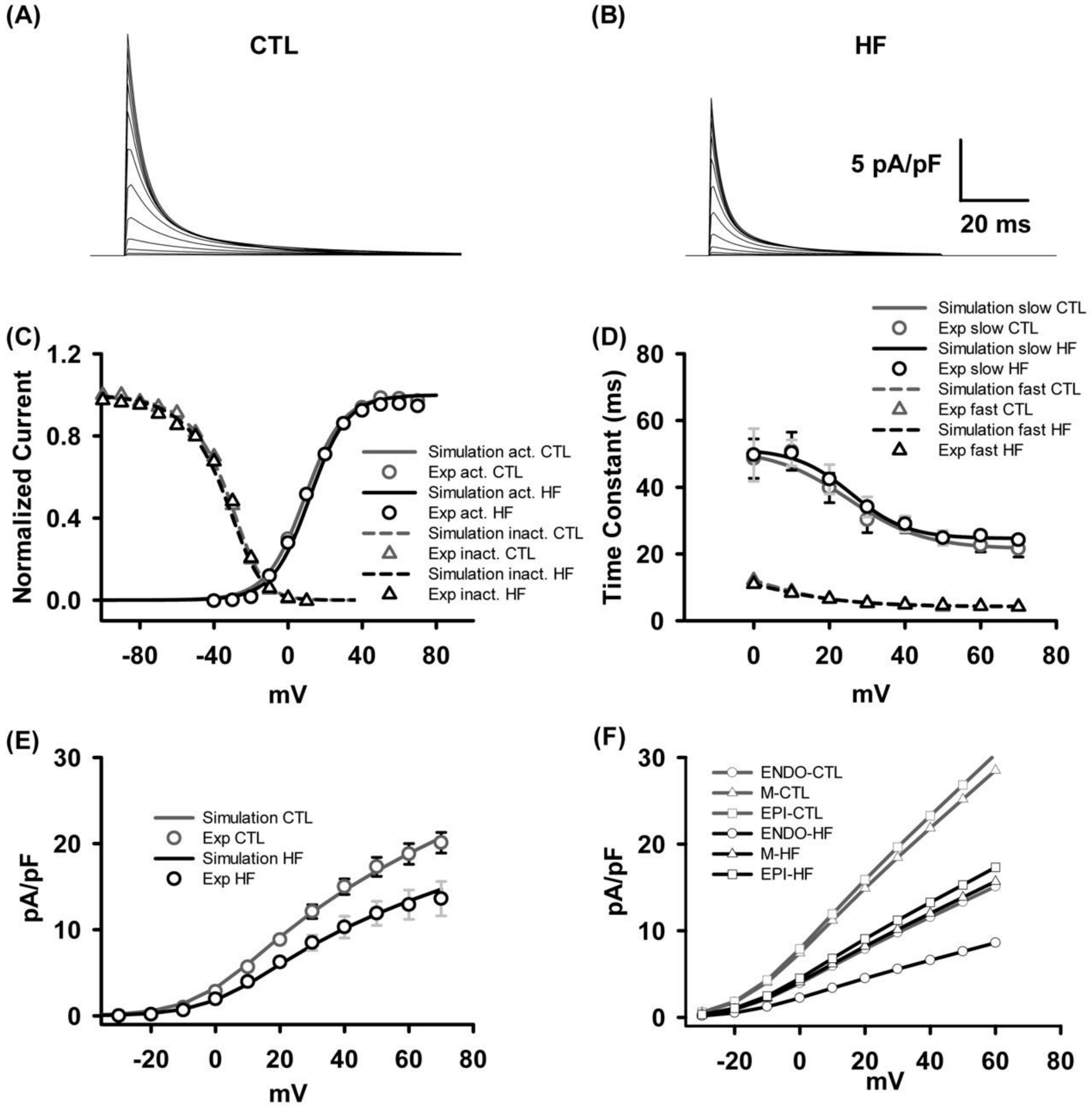

Supplementary Figure S5 Simulated I_{to1} in PF and ventricles. (A-E) Purkinje Fiber: comparison of the simulated I_{to1} properties in the CTL and HF conditions with experimental data (Han et al., 2001). I_{to1} was simulated during 100-ms voltage-clamp pulses from -40 mV to +70 mV from a holding potential of -70 mV. Current traces of I_{to1} in the CTL (A) and HF (B) conditions. (C) Steady state curves of the activation (Act.) and inactivation (Inact.). (D) Simulated fast (τfast) and slow (τslow) time constants. (E) Simulated I-V relationship. (F) Ventricles: Simulations of the I-V relationship of I_{to1} in the CTL and HF conditions. I_{to1} was elucidated by a sequence of 300-ms voltage-clamp pulses varying from -30 mV to +60 mV from a holding potential of -80 mV.
