# Supplementary material for: Pro-Arrhythmic Effects of Discontinuous Conduction at the Purkinje Fiber-Ventricle Junction Arising From Heart Failure-Induced Ionic Remodeling – Insights From Computational Modelling
Source: Front Physiol. 2022 Apr 25;13:877428. doi: 10.3389/fphys.2022.877428 (PMC9081695; doi:10.3389/fphys.2022.877428)
Supplement: Supplementary file 3 [file Table1.pdf]

Supplementary Table S1 I_{Na} channel properties in the HF condition

	**Reference**	**I_{Na}**	**Na_v 1.5**	**Species**
**Experimental Data**	Maltsev et al. (2002)	↓ 32%		Canine
	Zicha et al. (2004a)	↓ 36%	↓30% (protein)	Canine
	Valdivia et al. (2005)	↓ 39%	↔ (mRNA)	Canine
**Simulation Parameter**		↓ 32%		Canine

Supplementary Table S2 I_{NaL} channel properties in the HF condition

	Reference	I_{NaL}	Decay time	Species
**Experimental Data**	Maltsev et al. (2007)	↑ 30.64%	↑ 34%	Canine
	Maltsev et al. (2008)	↑ 29.1%	↑ 21.07%	Canine
**Simulation Parameter**		↑ 30%	↑34%	Canine

Supplementary Table S3 I_{to1} channel properties in the HF condition.

	Reference	I_{to1}	Kinetics	$K_v4.3$	Species
**Experimental Data**	Kaab et al. (1996)	↓ 65.7%	↔		Canine
	Zicha et al. (2004b)	↓ 55% Endo<br>↓ 72% Epi	↔		Canine
	Li et al. (2002)	↓ 43% Endo<br>↓ 45% M<br>↓ 43% Epi	↔		Canine
	Akar et al. (2005)			↓49% (mRNA) Endo<br>↓73% (protein) Endo<br>↓52% (mRNA) M<br>↓34% (protein) M<br>↓44% (mRNA) Epi<br>↓ 39% (protein) Epi	Canine
	Kaab et al. (1998)	↓ ～50%		↓30% (mRNA)	Human
	Beuckelmann et al. (1993)	↓ 36.3%			Human
	Li et al. (2004)	↓ 32.1%	↔		Human
	Zicha et al. (2004b)	↓			Human
	Borlak & Thum (2003)			↓15% (mRNA)	Human
	Petkova-Kirova et al. (2006)	↓ 51.4%			Mice
	Rose et al. (2005)	↓ 60%	↔		Rabbit
	Rozanski et al. (1997)	↓ 65%			Rabbit
**Simulation**		↓ 43% Endo<br>↓ 45% M<br>↓ 43% Epi	↔		Canine

Supplementary Table S4 I_{K1} channel properties in HF condition.

	Reference	I_{K1}	Kir2.1	Species
**Experimental Data**	Kaab et al. (1996)	↓ 32%		Canine
	Li et al. (2002)	↓ 41.1% Endo<br>↓ 40.7% M<br>↓ 40.9% Epi		Canine
	Akar et al. (2005)		↔ (mRNA&protein)	Canine
	Beuckelmann et al. (1993)	↓ 43.4%		Human
	Kaab et al. (1998)		↔ (mRNA)	Human
	Wang et al. (1998)		↔ (mRNA)	Human
	Li et al. (2004)	↓ 56.3%		Human
	Pogwizd et al. (2001)	↓ 49%		Rabbit
	Rose et al. 2005)	↓		Rabbit
	Rozanski et al. (1997)	↔		Rabbit
	Thuringer et al. (1996)	↓		Rat
**Simulation Parameter**		↓ 41.1% Endo<br>↓ 40.7% M<br>↓ 40.9% Epi		Canine

Supplementary Table S5 I_{Kr} and I_{Ks} channel properties in the HF condition.

	**Reference**	**I_{Kr}**	**HERG**	**I_{Ks}**	**KVLQT1**	**Species**
**Experimental Data**	Li et al. (2002)	↔		↓ 30%		Canine
	Akar et al. (2005)		↑(protein)		↔ (protein)	Canine
	Li et al. (2004)			↓ 61.7%		Human
	Kaab et al. (1998)		↔ (mRNA)			Human
	Tsuji et al. (2006)	↔		↓ 55%	↓51% (mRNA) ↓64% (mRNA)	Rabbit
**Simulation**		↔		↓ 30%		Canine

Supplementary Table S6 I_{CaL} channel properties in the HF condition.

	**Reference**	**I_{CaL}**	**$Ca_v1.2$**	**Species**
**Experimental Data**	Kaab et al. (1996)	↔		Canine
	O'Rourke et al. (1999)	↓ 13%		Canine
	Undrovinas et al. (1999)	↓ 33% (steady state)		Canine
	He et al. (2001)	↔		Canine
	Li et al. (2002)	↔		Canine
	Iyer et al. (2012)	↓ 45% Endo<br>↓ 26% M<br>↔ Epi<br>Activated at more negative voltage		Canine
	Beuckelmann et al. (1992)	↔		Human
	Beuckelmann & Erdmann (1992)	↔		Human
	Chen et al. (2002)	Activated at more negative voltage (shift by -7.64 mV)		Human
	Borlak&Thum (2003)		↓30%-50% (mRNA)	Human
	Piacentino et al. (2003)	Activated at more negative voltage		Human
	Yao et al. (1998)	↓ 43%		Rabbit
	Pogwizd et al. (1999)	↔		Rabbit
	Pogwizd et al. (2001)	↓ 12.5%		Rabbit
**Simulation**		↓ 13%<br>Activated shifted by -7.64 mV		Canine

Supplementary Table S7 Intracellular Ca^{2+} regulation properties in the HF condition.

	Reference	$[Ca^{2+}]_i$ systolic	$[Ca^{2+}]_i$ Resting	$[Ca^{2+}]_i$ decay time	SR function	NCX/NCX1	Species
Experimental Data	O'Rourke et al. (1999)	↓ 60%	↑	↑ 35%	↓ 28% (protein)		Canine
	Hoeker et al. (2009)	↓		↑			Canine
	Iyer et al. (2012)		↑ ～33 %	↑ 67.7% (Endo) ↑ 44.4% (M) ↑ 50.2% (Epi)			Canine
	Gwathmey et al. (1990)	↔	↑ 165.4%				Human
	Mercadier (1990)				↓ 47% (mRNA)		Human
	Beuckelmann et al. (1992)	↓ 50.8%	↑ 72.6%	↑			Human
	Beuckelmann & Erdmann (1992)	↓	↑	↑			Human
	Takahashi et al. (1992)				↓ 50% (mRNA)		Human
	Studer et al. (1994)				↓ 50% (mRNA, DCM) ↓ 45% (mRNA, CAD)	↑ 55% (mRNA, DCM) ↑41% (mRNA, CAD)	Human
	Hasenfuss et al. (1994)				↓ 36% (protein)		Human
	Meyer et al. (1995)				↓ 41% (protein, DCM)		Human
	Flesch et al. (1996)					↑ 36% (protein, DCM) ↑ 20% (protein, ICM)	Human
	Reinecke et al. (1996)					↑ (CAD) ↑ (DCM)	Human
	Lindner et al. (1998)	↓ 44.1%					Human

	Piacentino et al. (2003)	↓	↔		↓ 42% (SR content)		Human
	Yao et al. (1998)	↓ 58.7%			↓	↓ 23.7%	Rabbit
	Pogwizd et al. (1999)		↔			↑ 20%	Rabbit
	Pogwizd et al. (2001)				↓ ~40% (SR Content)	↑ 20%	Rabbit
	Baartscheer et al. (2003)	↓	↑	↑		↑	Rabbit
**Simulation**		↓ ~ 60%	↑	↑	↓ ~40% (NSR and JSR content)	↑ 20%	Canine

*DCM=dilated cardiomyopathy. ICM=ischemic cardiomyopathy. CAD=coronary artery disease.

Supplementary Table S8 Na^{+}/K^{+}-ATPase properties in the HF condition.

	Reference	Na^{+}/K^{+}-ATPase	Species
**Experimental Data**	Fan et al. (1993)	↓ 39.7% (activity)	Canine
	Kim et al. (1994)	↓ ～40% (protein)	Canine
	Norgaard et al. (1988)	↓ 40.8% (concentration)	Human
	Allen et al. (1992)	↔ (protein)	Human
	Shamraj et al. (1993)	↓ 42% (concentration)	Human
	Bundgaard et al. (1996)	↓ 40% (concentration)	Human
	Schwinger et al. (1999)	↓ 40% (protein)	Human
**Simulation**		↓ 40%	Canine

Supplementary Table S9 AP characteristics of the PF cell in the CTL and HF conditions.

	Reference	RP, mV		APA, mV		OS, mV		dV/dt_{max}, V/sec		PP, mV	
		CTL	HF	CTL	HF	CTL	HF	CTL	HF	CTL	HF
Experimental Data	Li et al. (1993)	-91.6 ±1.7	-82.1 ±1.7	113.1 ±0.8	105 ±4.9						
	Balati et al. (1998)	-89.6 ±0.9		124.6 ±1.7				505.0 ±32.7			
	Kondo et al. (1999)	-85		115				250			
	Balati et al. (2000)	-89 ±1.0<br>-87 ±0.6		112 ±3.5<br>117 ±1.2				416 ±14.0<br>445 ±14.6			
	Han et al. (2001)	-80.5 ±0.8	-79.0 ±0.9	111.6 ±1.4	112.4 ±1.2	28.5 ±1.0	24.4 ±0.8			2.7 ±1.4	9.0 ±1.4
	Dumaine & Cordeiro (2007)	-83.6 ±1.7		120						-10	
	Maguy et al. (2009)	-76 ±1	-78 ±1	107 ±3	99 ±2	30 ±3	21 ±2	383 ±46	248± 31		
**Simulation**		-85.5	-83.9	117.0	108.9	31.5	25.0	388.3	261.1	2.9	6.8

*RP, resting potential; APA, AP amplitude; OS, overshoot; dV/dt_{max}, maximum upstroke velocity; PP, plateau potential.

Supplementary Table S10 Rate dependence of APD for the canine Endo cell

	Reference	0.2 Hz	0.25 Hz	0.5 Hz	1 Hz
**Experimental Data**	Li et al. (2004)				313±22 ms
	Antzelevitch et al. (1999)			266±21 ms	249±18 ms
	Shimizu et al. (1999)			265±9 ms<br><br>266±9 ms	
	Balati et al. (1998)				238.6±5.0 ms
	Yan et al. (1998)			266±21 ms	249±18 ms
	Shimizu et al. (1997)			246±1 ms	
	Anyukhovsky et al. (1996)			229±5 ms	
	Liu et al. (1993)		287±11 ms		
	Sicouri et al. (1994)	219±17 ms [base]<br><br>239±11 ms [apex]			
	Liu et al. (1993)			252±23 ms	
	Sicouri et al. (1991)			215.5±19.2 ms	
**Simulation**		274.6 ms	274.4 ms	272.2 ms	260.3 ms

Supplementary Table S11 Rate dependence of APD for the canine M cell

	Reference	0.2 Hz	0.25 Hz	0.5 Hz	1 Hz
Experimental Data	Li et al. (2004)				423±26 ms
	Antzelevitch et al. (1999)			281±25 ms	260±21 ms
	Shimizu et al. (1999)			280±6 ms 275±7 ms	
	Undrovinas et al. (1999)	390±70 ms		390±80 ms	
	Balati et al. (1998)				258.7±4.5 ms
	Shimizu et al. (1998)			284±13 ms	
	Yan et al. (1998)			281±25 ms	260±21 ms
	Yan & Antzelevitch (1998)				260±21 ms
	Shimizu et al. (1997)			267±7 ms	
	Anyukhovsky et al. (1996)			344±8 ms	
	Liu et al. (1993)		358±16 ms		
	Sicouri et al. (1995)	361±57.7 ms [with $[K^+]_o$ = 4]			
	Sicouri et al. (1995)	385±66 ms [base] 393±67 ms [apex]			
	Liu et al. (1993)			402±51 ms	
	Sicouri et al. (1995)			311.5±39.6 ms	
**Simulation**		322.2 ms	322.1 ms	318.7 ms	303.0 ms

Supplementary Table S12 Rate dependence of APD for the canine Epi cell

	Reference	0.2 Hz	0.25 Hz	0.5 Hz	1 Hz
**Experimental Data**	Li et al. (2002)				343±22 ms
	Antzelevitch et al. (1999)			217±24 ms	207±20 ms
	Shimizu et al. (1999)			223±6 ms<br>219±6 ms	
	Balati et al. (1998)				222.1±5.3 ms
	Shimizu et al. (1998)			226±17 ms	
	Yan et al. (1998)			217±24 ms	207±20 ms
	Shimizu et al. (1997)			220±4 ms	
	Anyukhovsky et al. (1996)			249±10 ms	
	Liu et al. (1995)		262±12 ms		
	Sicouri et al. (1994)	231±23 ms [base]<br><br>263±23 ms [apex]			
	Liu et al. (1993)			233±21 ms	
	Sicouri et al. (1991)			222.3±26.2 ms	
**Simulation**		262.5 ms	262.3 ms	260.3 ms	252.8 ms

Supplementary Table S13 Rate dependence of APD for the canine PF cell.

	Reference	0.5 Hz (ms)		1 Hz (ms)	
		**CTL**	**HF**	**CTL**	**HF**
**Experimental Data**	Robinson et al. (1987)	333±37			
	Zaza et al. (1989)			313±16 with $[K^+]_o$=2.7mM<br>319±18 with $[K^+]_o$ = 4.0 mM	
	Li et al. (1993)			313.8±4.8	273.2±6.5
	Balati et al. (1998)			324.8±15.4	
	Yan &Antzelevitch (1998)			299±17	
	Balati et al. (2000)			275±16.6<br>321±9.9	
	Han et al. (2001)			373±8	361±8
	Maguy et al. (2009)			320±12	282±17
**Simulation**		345.2	335.0	330.2	323.8

Supplementary Table S14 APD increment in the HF condition-simulation & experimental data.

	Reference	APD	Species
**Experimental Data**	Undrovinas et al. (1999)	↑49%	Canine
	Kaab et al. (1996)	↑30%	Canine
	Maltsev et al. (1998)	↑191.78%	Canine
	Beuckelmann et al. (1992)	↑59.94%	Human
	Rose et al. (2005)	↑36.8%	Rabbit
**Simulation**		↑25.6% Endo<br>↑55.9% M<br>↑ 27.2% Epi	Canine
