# Supplementary material for: Pro-Arrhythmic Effects of Discontinuous Conduction at the Purkinje Fiber-Ventricle Junction Arising From Heart Failure-Induced Ionic Remodeling – Insights From Computational Modelling
Source: Front Physiol. 2022 Apr 25;13:877428. doi: 10.3389/fphys.2022.877428 (PMC9081695; doi:10.3389/fphys.2022.877428)
Supplement: Supplementary file 4 [file Image9.pdf]

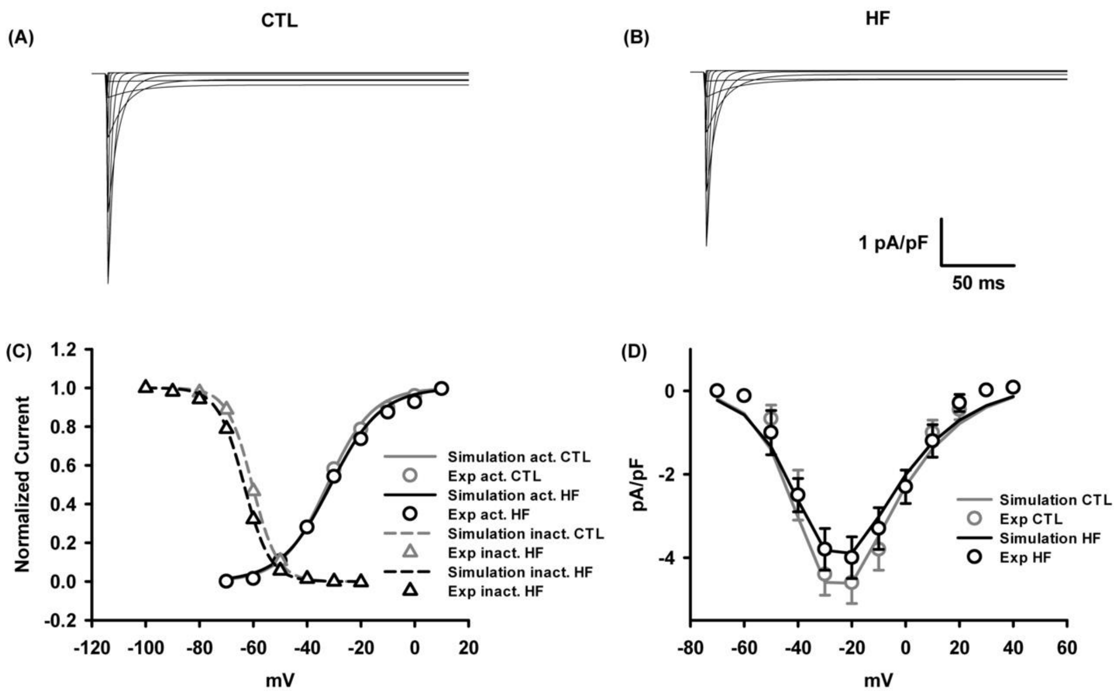

Supplementary Figure S9 Simulated I_{CaT} in PF. Comparison of the simulated I_{CaT} properties in Purkinje Fiber in the CTL and HF conditions. I_{CaT} was simulated during 250-ms voltage-clamp pulses from -70 mV to +40 mV with a holding potential of -90 mV. Simulated current traces of I_{CaT} in the CTL (A) and HF (B) conditions. (C) Steady-state curves of the activation (Act.) and inactivation (Inact.), which were compared to the experimental data (Han et al., 2001). (D) Simulated I-V relationship as compared to the experimental data (Han et al., 2001).
