# Supplementary material for: Pro-Arrhythmic Effects of Discontinuous Conduction at the Purkinje Fiber-Ventricle Junction Arising From Heart Failure-Induced Ionic Remodeling – Insights From Computational Modelling
Source: Front Physiol. 2022 Apr 25;13:877428. doi: 10.3389/fphys.2022.877428 (PMC9081695; doi:10.3389/fphys.2022.877428)
Supplement: Supplementary file 5 [file Image10.pdf]

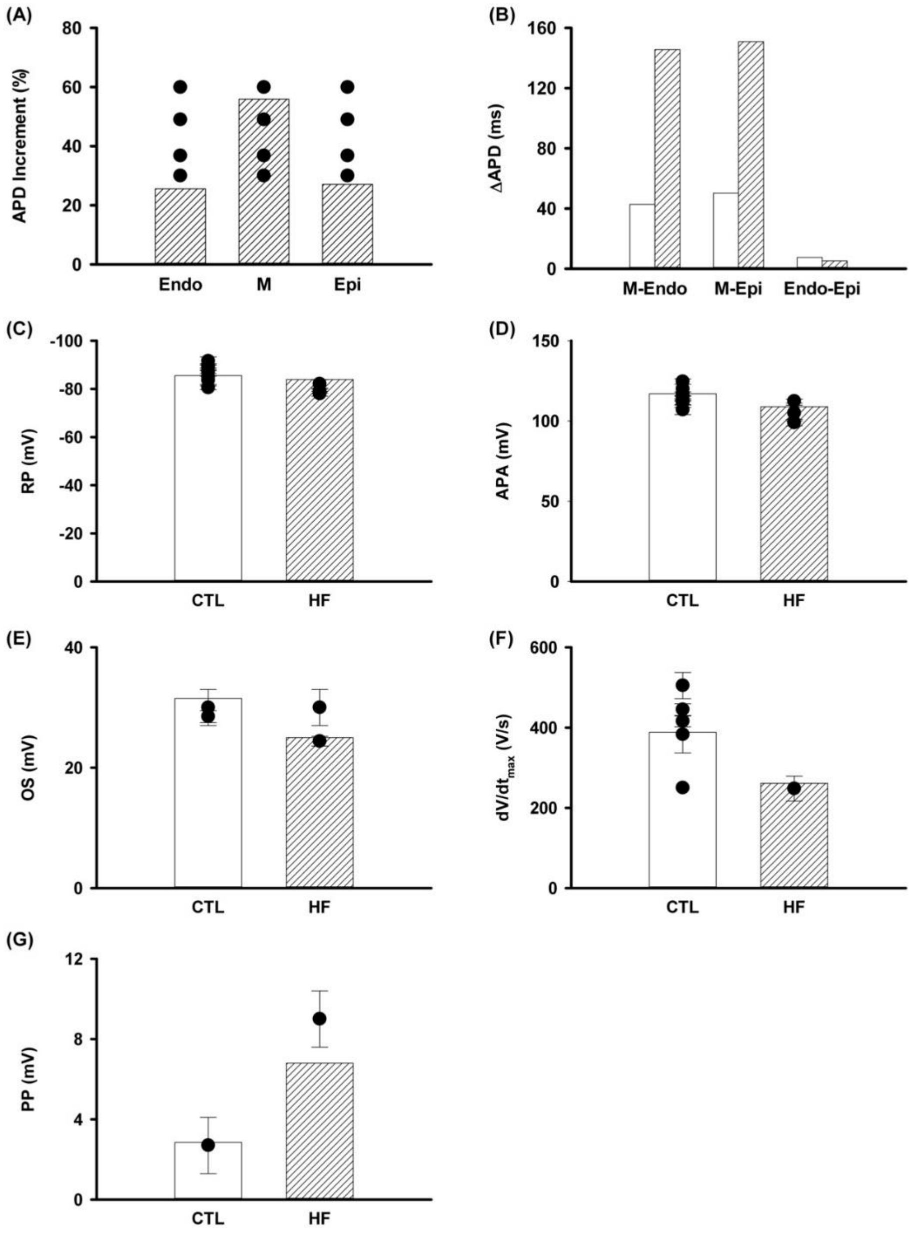


**Supplementary Figure S10** Characteristics of AP. (A) APD increment of ventricles in the HF condition. Computed APD increment in the Endo, M and Epi cells are shown in bar charts, and experimental data are shown in dots. (B) Regional APD differences (△APD) between ventricular cells in both CTL and HF conditions. (C-G) Computed AP characteristics of the PF cell in the CTL and HF condition. AP characteristics computed from the model (bar), which were compared to the respective experimental data (dot) in the CTL and HF conditions. In C, RP (RMP): resting membrane potential. Experimental data are listed in Supplementary Table S9 and Supplementary Table S14.
