# Supplementary material for: Pro-Arrhythmic Effects of Discontinuous Conduction at the Purkinje Fiber-Ventricle Junction Arising From Heart Failure-Induced Ionic Remodeling – Insights From Computational Modelling
Source: Front Physiol. 2022 Apr 25;13:877428. doi: 10.3389/fphys.2022.877428 (PMC9081695; doi:10.3389/fphys.2022.877428)
Supplement: Supplementary file 6 [file Image6.pdf]

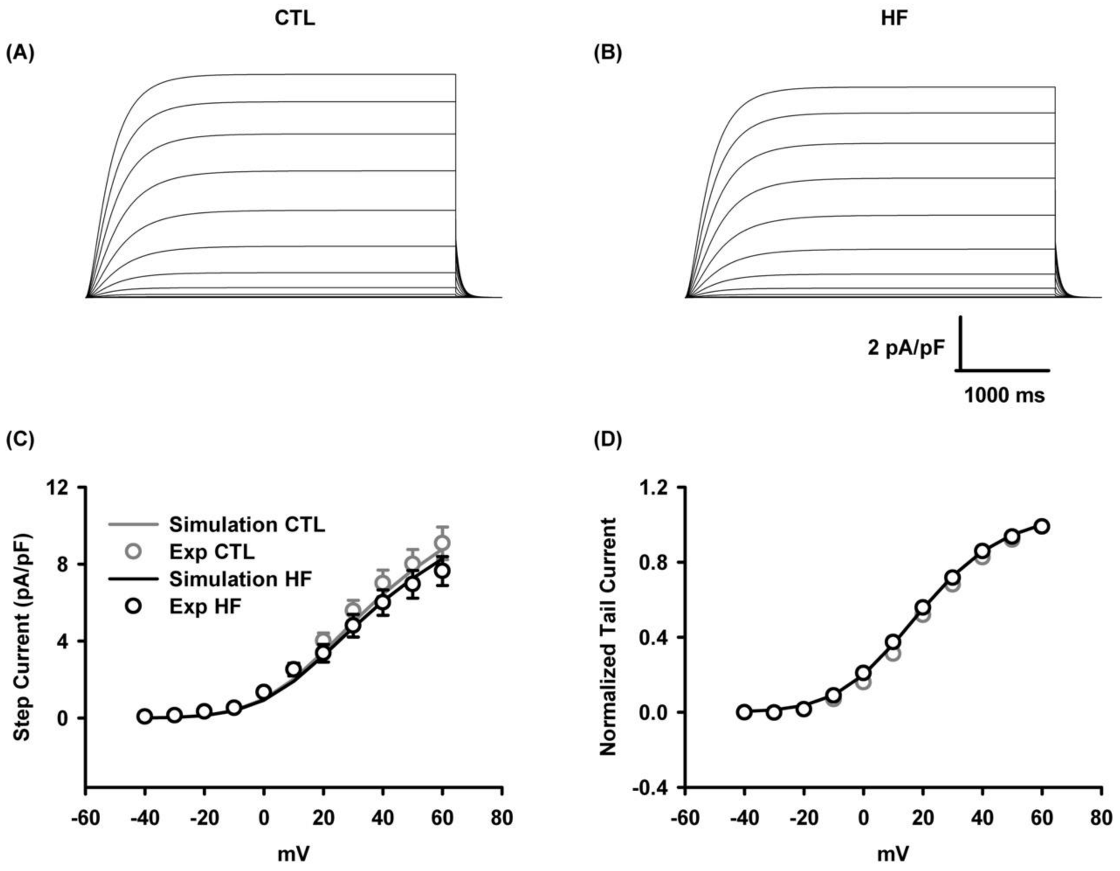

Supplementary Figure S6 Simulated I_{Ks} in PF. Comparison of the simulated I_{Ks} in the CTL and HF conditions in Purkinje Fiber. I_{Ks} was simulated during 4000-ms voltage-clamp pulses from -40 mV to +60 mV followed by a 2000-ms repolarising pulse to -40 mV with a holding potential of -60 mV. Simulated results were compared to the experimental data (Han et al., 2001). Simulated I_{Ks} current traces in the CTL (A) and the HF (B) conditions. (C) Simulated I-V relationship of the step current. (D) Simulated normalized tail current.
