# Supplementary material for: Pro-Arrhythmic Effects of Discontinuous Conduction at the Purkinje Fiber-Ventricle Junction Arising From Heart Failure-Induced Ionic Remodeling – Insights From Computational Modelling
Source: Front Physiol. 2022 Apr 25;13:877428. doi: 10.3389/fphys.2022.877428 (PMC9081695; doi:10.3389/fphys.2022.877428)
Supplement: Supplementary file 7 [file Image13.pdf]

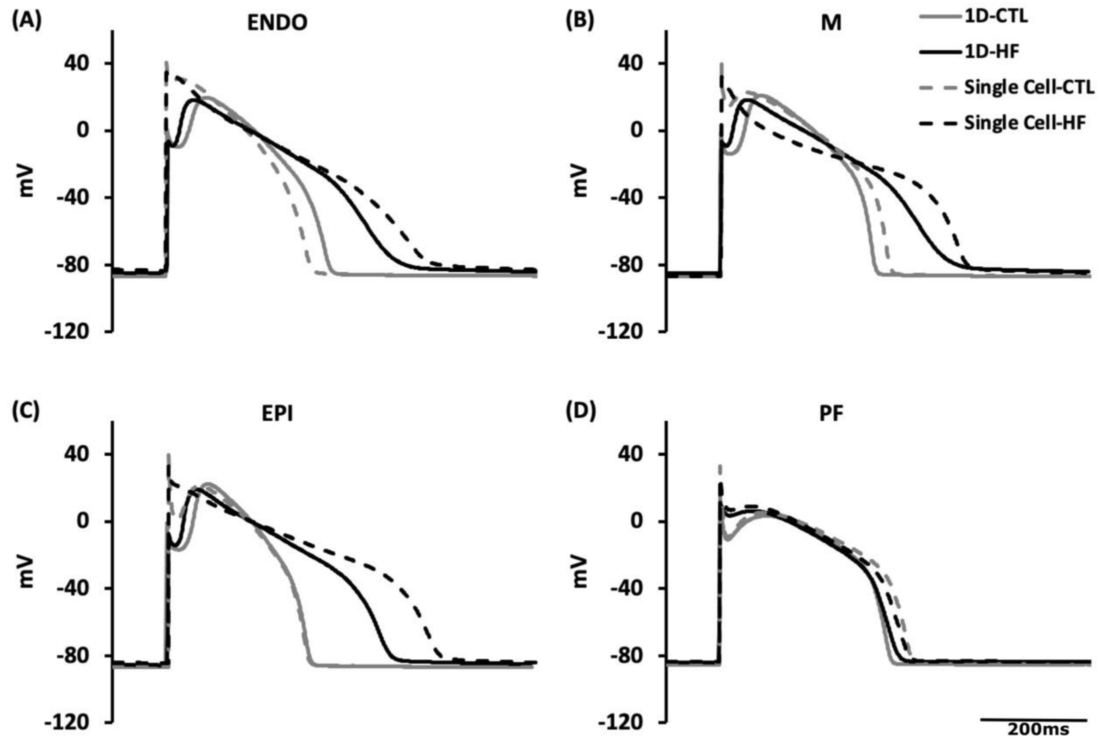


**Supplementary Figure S13** Simulated cell APs in different regions of the 1D CTR and PF-ventricle strand. The APs were superimposed on those from single cell models. A: Endo region. B: M region. C: Epi region. D: PF region.
