# Supplementary material for: Pro-Arrhythmic Effects of Discontinuous Conduction at the Purkinje Fiber-Ventricle Junction Arising From Heart Failure-Induced Ionic Remodeling – Insights From Computational Modelling
Source: Front Physiol. 2022 Apr 25;13:877428. doi: 10.3389/fphys.2022.877428 (PMC9081695; doi:10.3389/fphys.2022.877428)
Supplement: Supplementary file 8 [file Image8.pdf]

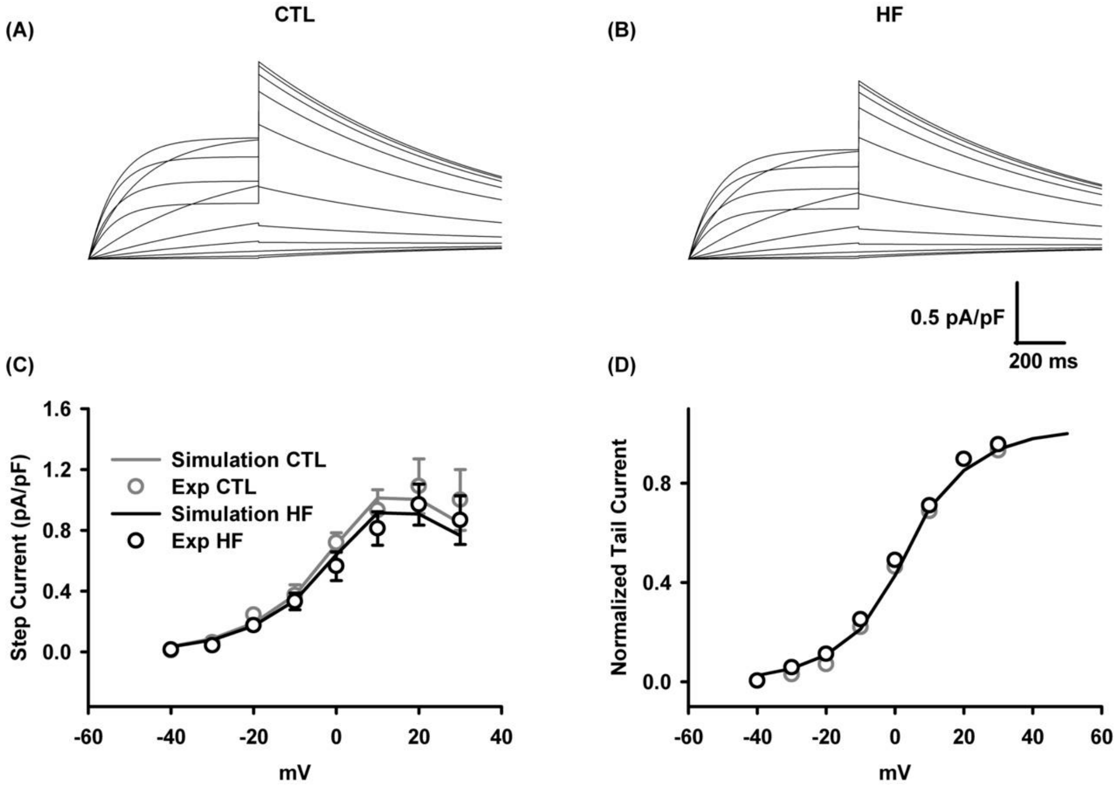

Supplementary Figure S8 Simulated I_{Kr} in PF. Comparison of the simulated I_{Kr} in Purkinje Fiber in the CTL and HF conditions. Currents were obtained during 700-ms voltage-clamp pulses from -40 mV to +50 mV followed by a 1000-ms repolarising pulse to -30 mV with a holding potential of -50 mV. Simulated I_{Kr} current traces in the CTL (A) and HF (B) conditions. (C) I-V relationship of the step current compared to the experimental data (Han et al., 2001). (D) Normalized tail current compared to the experimental data (Han et al., 2001).
