# Supplementary material for: Pro-Arrhythmic Effects of Discontinuous Conduction at the Purkinje Fiber-Ventricle Junction Arising From Heart Failure-Induced Ionic Remodeling – Insights From Computational Modelling
Source: Front Physiol. 2022 Apr 25;13:877428. doi: 10.3389/fphys.2022.877428 (PMC9081695; doi:10.3389/fphys.2022.877428)
Supplement: Supplementary file 9 [file Image4.pdf]

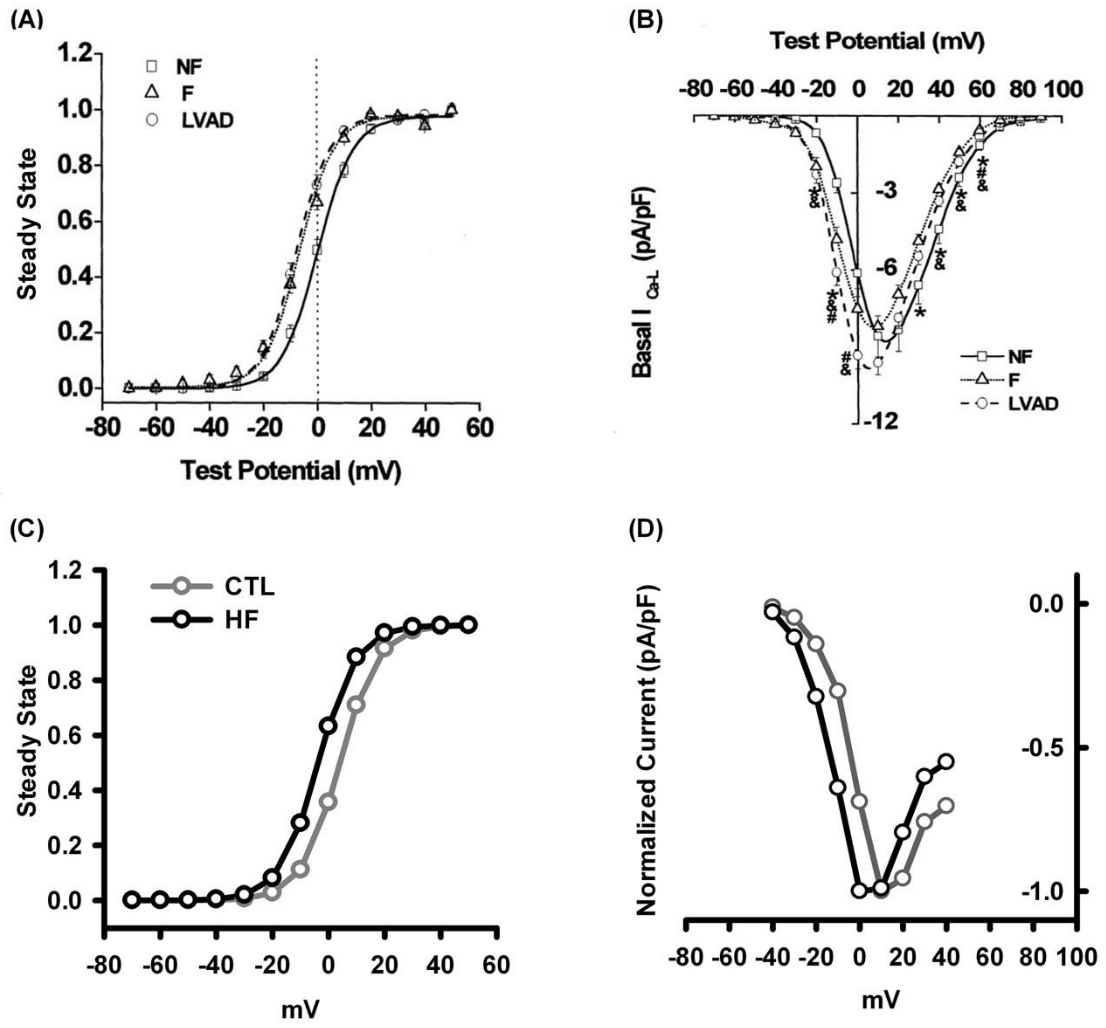

Supplementary Figure S4 Simulated I_{CaL} in ventricles. I_{CaL} ventricular cells properties in the CTL and HF conditions. Experimental data (Chen et al., 2002) (Han et al., 2001) of the activation steady state curve (A) and the I-V relationship (B) in the CTL (NF) and HF (F) conditions. (C) Simulated activation steady state curves. I_{CaL} was activated at more negative voltages (shifted 7.64 mV to the left) (D) Simulated I-V relationship of I_{CaL} (normalized) obtained by using a sequence of 1000-ms voltage-clamp pulses varying from -40 mV to +40 mV from a holding potential of -70 mV. (*LVAD = left ventricular assist device)
