# Supplementary material for: Pro-Arrhythmic Effects of Discontinuous Conduction at the Purkinje Fiber-Ventricle Junction Arising From Heart Failure-Induced Ionic Remodeling – Insights From Computational Modelling
Source: Front Physiol. 2022 Apr 25;13:877428. doi: 10.3389/fphys.2022.877428 (PMC9081695; doi:10.3389/fphys.2022.877428)
Supplement: Supplementary file 10 [file Image2.pdf]

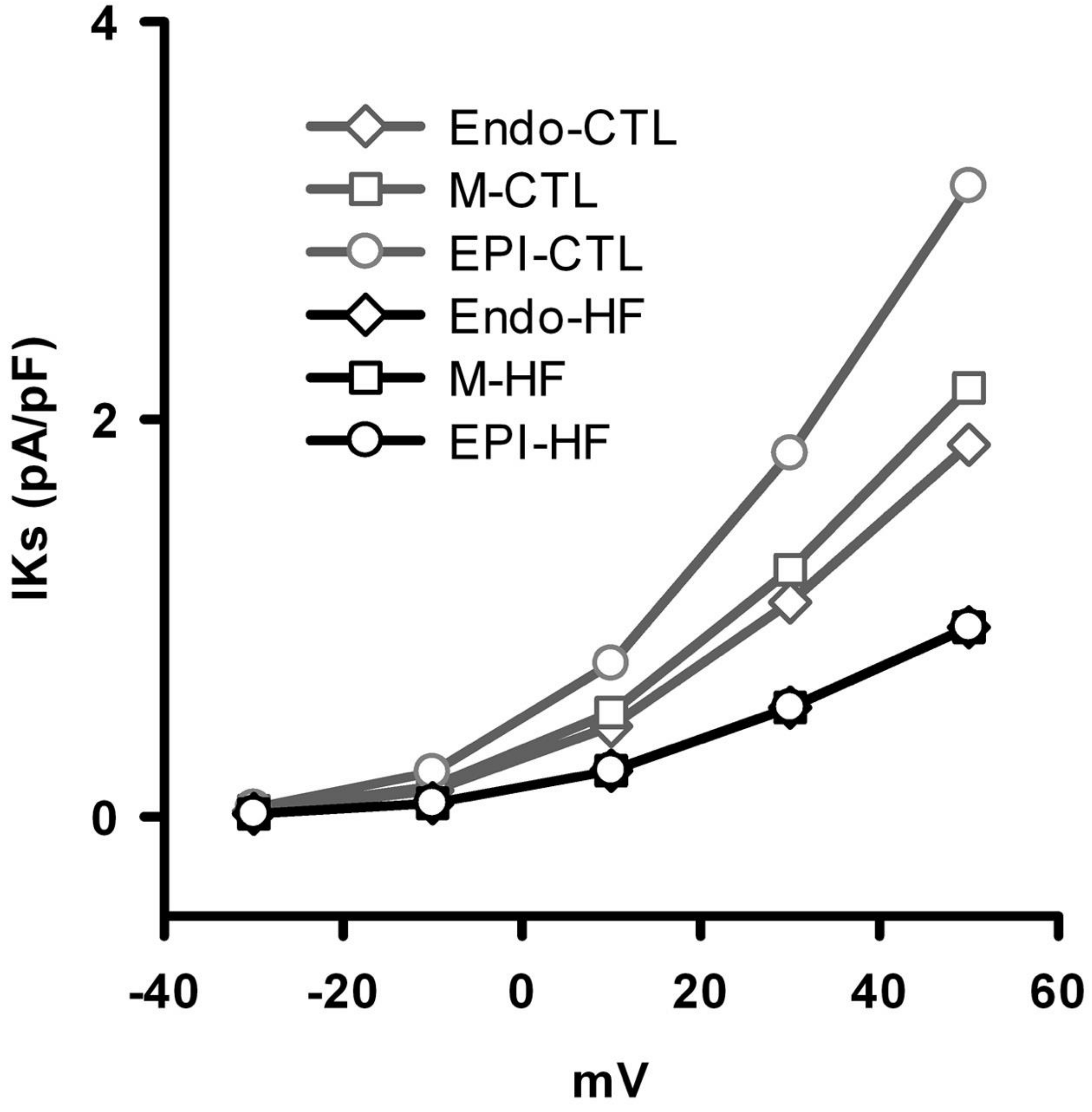

Supplementary Figure S2 Simulated I_{Ks} in ventricles. I_{Ks} was simulated during a sequence of 4000-ms voltage-clamp pulses varying from -30 mV to +50 mV from a holding potential of -60 mV followed by a 2000-ms repolarising pulse to -30 mV. The current density at the end of the step voltage was measured in the CTL (A) and HF (B) conditions.
