# Supplementary material for: Pro-Arrhythmic Effects of Discontinuous Conduction at the Purkinje Fiber-Ventricle Junction Arising From Heart Failure-Induced Ionic Remodeling – Insights From Computational Modelling
Source: Front Physiol. 2022 Apr 25;13:877428. doi: 10.3389/fphys.2022.877428 (PMC9081695; doi:10.3389/fphys.2022.877428)
Supplement: Supplementary file 11 [file Image3.pdf]

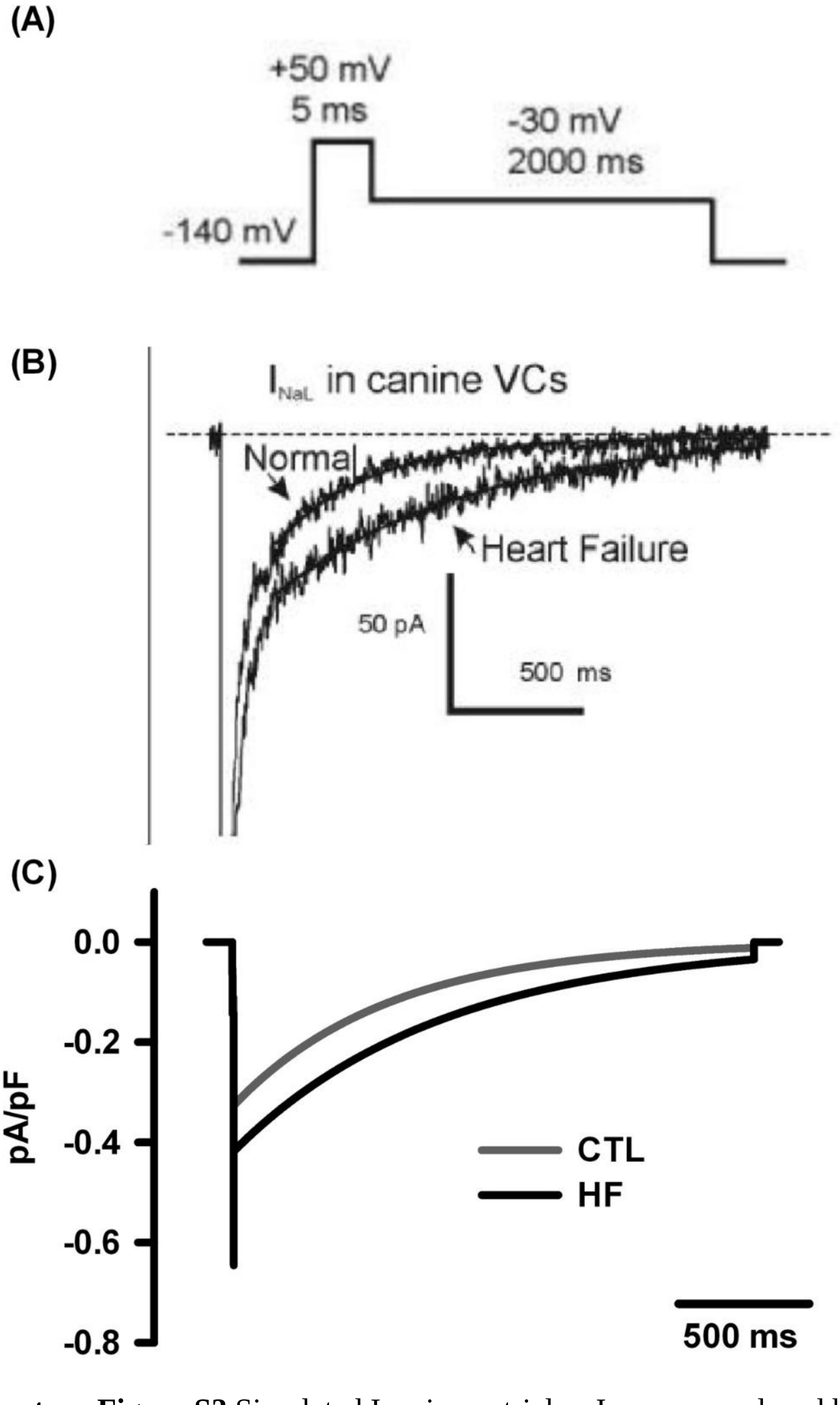

Supplementary Figure S3 Simulated I_{NaL} in ventricles. I_{NaL} was produced by 2000 ms voltage-clamp pulses to -30 mV from a holding potential of -140mV followed by a 5 ms voltage-clamp to +50 mV (shown at the top panel). (A) Experimental data of I_{NaL} current traces but truncated at -150 pA (Maltsev et al., 2007). (B) Simulated I_{NaL} current traces (normalized by the cell capacitance).
