# Supplementary material for: Pro-Arrhythmic Effects of Discontinuous Conduction at the Purkinje Fiber-Ventricle Junction Arising From Heart Failure-Induced Ionic Remodeling – Insights From Computational Modelling
Source: Front Physiol. 2022 Apr 25;13:877428. doi: 10.3389/fphys.2022.877428 (PMC9081695; doi:10.3389/fphys.2022.877428)
Supplement: Supplementary file 12 [file DataSheet1.pdf]

Supplementary Text

Supplementary S1 Development of single cell models

S1.1 CTL ventricular models

S1.1.1 Inward rectifier K+ current: I_{K1}

Based on the research conducted by Liu et al. (Liu et al., 1993), the ratios of the maximum conductance of I_{K1} for the Endo, M and Epi cells were set to 0.7 for Endo/Epi and 1.1 for M/Epi. Simulated I_{K1} current was shown in Supplementary Figure S1 A&B, which were consistent with experimental observations. Our results suggested I_{K1} was greater expressed in the M cell and less expressed in the Endo cell.

S1.1.2 Slow delayed rectifier K^+ current: I_{Ks}

The ratios of the I_{Ks} maximal conductance were modified to 0.59 for Endo/Epi and 0.68 for M/Epi respectively based on reports by Liu & Antzelevitch (1995) and Li et al. (2002). Results were shown in Supplementary Figure S2.

S1.1.3 Intracellular Ca^{2+} transient: $[Ca^{2+}]_i$

The formulation for the $[Ca^{2+}]_i$ was modified to match the experimental observations of O’Rourke et al. (1999) by reducing the maximum uptake from the myoplasmic to the network sarcoplasmic reticulum (NSR) and the myoplasmic volume by 20% and 50% respectively.

S1.2 HF ventricular models

S1.2.1 Fast Na^+ current: I_{Na}

Experimental studies have shown that the current density of I_{Na} was reduced in the HF condition (Maltsev et al., 2002, Zicha et al., 2004, Valdivia et al., 2005). Similar down regulation of Na_v 1.5 has also been observed (see Supplementary Table S1). But no alternation in I_{Na} kinetics has been noted. In simulated HF canine ventricular cells, the maximal conductance of I_{Na} was reduced by 32% in all three types of ventricular cells based on the experimental data of Maltsev et al. (2002).

S1.2.2 Late Na^+ current: I_{NaL}

I_{NaL} was modified by increasing its maximum conductance and inactivation time constant by 30% and 34% respectively based on the experimental data of Maltsev et al. (2007). As shown in Supplementary Figure S3, the simulated I_{NaL} showed an

increased current density and a slowed decay time in the HF condition, which was consistent with experimental findings (see Supplementary Table S2).

S1.2.3 Transient outward K^+ current: I_{to1}

Experimental studies have shown that in canine ventricles, HF did not alter the kinetics of I_{to1}, but reduced its current density as well as K_v 4.3 expressions (see Supplementary Table S3). In the simulation of HF, the maximal conductance of I_{to1} was reduced by 43% in the Endo and Epi cells and 45% in the M cell (Li et al., 2002).

S1.2.4 Inward rectifier K^+ current: I_{K1}

In the simulation of HF, I_{K1} maximal conductance were reduced by 41.1%, 40.7% and 40.9% in the Endo, M and Epi cells respectively based on the experimental data as shown in Supplementary Table S4. Simulations results were shown in Supplementary Figure S1.

S1.2.5 Fast and slow delayed rectifier K^+ current: I_{Kr} and I_{Ks}

In the simulation of HF, I_{Ks} was modified and I_{Kr} remained the same as the CTL condition (see Supplementary Table S5). Based on the experimental finding of Li et al. (2002), the I_{Ks} current was reduced by 30% and the transmural heterogeneity of I_{Ks} among the Endo, M and Epi cells was removed. Supplementary Figure S2 in illustrated the simulated I_{Ks} I-V relationship.

S1.2.6 L-type Ca^{2+} current: I_{CaL}

Some studies showed the evidence (see Supplementary Table S6) of I_{CaL} remodelled by HF in ventricular cells. In the simulation of HF, modifications to I_{CaL} was mainly based on the data from human on changed I_{CaL} kinetics (Chen et al., 2002) and the data from canine on altered I_{CaL} density (O'Rourke et al., 1999). The steady state activation curve of I_{CaL} was shifted 7.64 mV to the left (Supplementary Figure S4).

S1.2.7 Intracellular Ca^{2+} transient: $[Ca^{2+}]_i$

There is abundant evidence from various studies (see Supplementary Table S7) showing that in the HF condition, the systolic $[Ca^{2+}]_i$ level was reduced while the decay process of $[Ca^{2+}]_i$ from the peak to the resting concentration levels was slowed down in ventricular cells. There is also evidence of a reduced activity in the SR uptake (Gupta et al., 1997) and down regulation of Ca^{2+} uptake current (Whitmer et al., 1988). Piacentino et al. (2003) showed a ～40% reduction in the SR content in human ventricular cells and Pogwizd et al. (2001) observed a similar change in rabbit. There is no experimental data available for the NCX density for canine ventricular

cells in the HF condition. But several studies have reported an up regulation of its protein and mRNA levels in human ventricular cells.

In the simulation of HF, the experimental observations mentioned above were taken into account for simulating HF-induced remodelling on the intracellular Ca^{2+} regulation. Specifically, the Ca^{2+} uptake to the NSR was reduced as well as the Ca^{2+} leak in the SR and the Ca^{2+} release in the junctional sarcoplasmic reticulum (JSR). These were achieved by reducing the maximal sarcoplasmic reticulum Ca^{2+}-ATPase (SERCA) uptake (~87.5%), SR leak (~35%) and JSR release (~40%) coefficients. The modifications resulted in a ~40% reduction in both NSR and JSR Ca^{2+} contents. In addition, the NCX density was increased by 20% in the HF condition.

S1.2.8 Na^+/K^+-ATPase: I_{NaK}

In the simulation of HF, the maximum I_{NaK} was reduced by 40% based on experimental data (see Supplementary Table S8).

S1.3 CTL PF models

S1.3.1 Fast Na^+ current: I_{Na}

In order to have the resultant AP upstroke features matched to experimental data (Han et al., 2001) (Maguy et al., 2009), we shifted the steady state curves of the activation by 10 mV to the right and that of the inactivation by 10 mV to the left. The formulations and parameters for the inactivation time constant were adopted from the Benson et al. (2008) Endo model. The maximum conductance of I_{Na} was increased by 3% in the Aslanidi et al. (2009) model.

S1.3.2 Transient outward K^+ current: I_{to1}

Steady states of the activation and inactivation curves of I_{to1} of the PF cell were modified to fit the experimental data (Han et al., 2001). This was achieved by shifting the relevant equations of the Aslanidi et al. (2009) model by 2 mV to the right (see Supplementary Figure S5C). In addition, the model equation for the fast inactivation time constant (τ_{fast}), refitted to the experimental data of Han et al. (2001) (see Supplementary Figure S5D). With these modifications, the simulated I-V relationship matched closely to the experimental data of Han et al. (2001) (see Supplementary Figure S5E).

S1.3.3 Inward rectifier K^+ current: I_{K1}

For the canine PF cell, I_{K1} is not a time-dependent channel current and can be simulated by a simple voltage-dependent formulation as shown in Equation A.1 and A.2 (Kharche et al., 2008)

$$I_{K1} = G_{K1}(V_m - E_K) \quad \text{(Equ S1.1)}$$

$$G_{K1} = g_{K1}\left(\alpha + \frac{\beta}{1+\exp(b(V_m - c))}\right) \quad \text{(Equ S1.2)}$$

where V_m is the membrane potential, E_K the K^+ reversal potential, g_{K1} the maximal conductance, c the half maximal voltage, α the fraction of the voltage-independent conductance, β the fraction of the voltage-dependent conductance and b the steepness of the G_{K1}-voltage relationship. Equation 2 and 3 were then reformulated by fitting them to the experimental data of Han et al. (2001) under the voltage-clamp protocol as shown in Supplementary Figure S1C&D.

S1.3.4 Slow delayed rectifier K^+ current: I_{Ks}

I_{Ks} were modified by refitting its activation steady-state variable and maximum conductance to the experimental data of Han et al. (2001). This resulted in a shift of the activation steady-state variable by 4.5 mV to the left and a reduction of the maximum conductance by 15% compared to the model of Aslanidi et al. (2009). Supplementary Figure S6 showed the simulated I_{Ks} step and normalized tail currents, both of which well-matched to the experimental data (Han et al., 2001).

S1.3.5 L-type Ca^{2+} current: I_{CaL}

The equations for I_{CaL} in Purkinje fiber cell were re-formulated based on the experimental data of Han et al. (2001). This resulted in a 2.26-mV and 6.1-mV left shift in steady-state variables of the voltage-dependent activation and inactivation curves respectively. Equations for the fast (τ_f) and slow (τ_{f2}) time constants of the inactivation also refitted to the experimental data (Han et al., 2001). This resulted in a shift of τ_f by 3.59 mV to the left and τ_{f2} by 0.2 mV to the right respectively. To fit the I-V relationship experimental data, the maximum conductance of I_{CaL} was increased by 12%. Details of all altered I_{CaL} channel kinetics and their comparisons to the experimental data of Han et al (2001) were shown in Supplementary Figure S7.

S1.4 HF PF models

S1.4.1 Fast Na^+ current: I_{Na}

The maximum conductance of I_{Na} was reduced by 40% in order to have the resultant AP upstroke features matched to experimental data in the HF condition (Han et al., 2001), (Maguy et al., 2009).

S1.4.2 Transient outward K^+ current: I_{to1}

In the simulation of HF in the canine PF cell, the kinetics and current density of I_{to1} were modified to fit the experimental data of Han et al. (2) with 1-mV downward shift

in the fast inactivation time constant curve (τ_{fast}) as well as a 2.5ms reduction in its slow inactivation time (τ_{slow}) amplitude. In addition, there was a marked reduction in the maximal channel conductance of I_{to1} by 30%.

Supplementary Figure S5A-E shows simulated steady states of the activation, inactivation and its time constants as well as the I-V relationship of Purkinje Fiber cells in both CTL and HF conditions. For all of these, simulation data matched to the experimental data of Han et al. (2001). Current traces of I_{to1} showed a 29% reduction in its peak density in the HF condition compared to the CTL condition (Supplementary Figure S5A&B). Simulation data also suggested that there were slight differences in steady states of the activation and inactivation curves between the CTL and HF conditions, but the amplitude of the slow inactivation time constant was increased (Supplementary Figure S5D) as well as the significant reduction in I_{to1} density (Supplementary Figure S5E) in the HF condition, which was consistent with a reduction of K_v 4.3 protein (Maguy et al., 2009) in the canine PF cell.

S1.4.3 Inward rectifier K^+ current: I_{K1}

I_{K1} was modified with a 17.5-mV right shift in its voltage-dependent conductance (G_{K1}), and a 5% increase of the maximum conductance g_{K1}. Supplementary Figure S1C&D showed the simulated I_{K1} in the HF condition, which well-matched to the experimental data of Han et al. (2001).

S1.4.4 Fast and slow delayed rectifier K^+ current: I_{Kr} and I_{Ks}

Experimental data (Han et al., 2001) of I_{Kr} showed no significant difference in its kinetics between the CTL and HF conditions, but a dramatic reduction of 10% in its maximum channel conductance. The resultant I_{Kr} was shown in Supplementary Figure S8. Both the simulated I_{Kr} step and normalized tail currents during the voltage-clamp protocol matched to the experimental data (Han et al., 2001) in the HF condition.

Experimental data (Han et al., 2001) of I_{Ks}, on the other hand, showed a shift in its steady state by 6 mV to the right and a 5.7% reduction in its maximum conductance in the HF condition. After modifying the model with these experimental observations (Supplementary Figure S2), the resultant I_{Ks} step and normalized tail currents during the voltage-clamp protocol matched well to the experimental observation (Han et al., 2001).

S1.4.5 L-type Ca^{2+} current: I_{CaL}

Experimental data (Han et al., 2001) suggested that in the HF condition I_{CaL} was activated and inactivated at more negative voltages, which involved a 2.3-mV and 2.4-mV left shift in its steady states of the activation and inactivation curves respectively. The fast and slow time constants of the inactivation process were

increased, which can be reproduced by a 22.9-mV and 13.6-mV upward shift in their expressions respectively. Supplementary Figure S7 showed simulated steady states of the activation, inactivation and its time constants as well as I-V relationship. For all of these, simulation data well matched to the experimental data of Han et al. (2001).

S1.4.6 T-type Ca^{2+} current: I_{CaT}

In the simulation of HF, I_{CaT} was modified in its activation and inactivation steady state curves as well as the current density based on the available experimental data (Han et al., 2001). The maximum conductance of I_{CaT} was reduced by 10%; the activation and inactivation steady-state curves were shifted by 1.2 mV and 3.0 mV to the left respectively. As shown in Supplementary Figure S9, simulation results matched well to the experimental data (Han et al., 2001).

Supplementary S2 Canine Ventricular Cell Models

S2.1 Model Equations

2.1.1. Fast Na^+ Current: I_{Na}

$$I_{Na} = g_{Na} m^3 hj(V - E_{Na}) \quad \text{(EQ S2.1)}$$

$$\frac{dm}{dt} = \frac{m_\infty - m}{\tau_m} \quad \text{(EQ S2.2)}$$

$$m_\infty = \frac{\alpha_m}{\alpha_m + \beta_m} \quad \text{(EQ S2.3)}$$

$$\tau_m = \frac{1.0}{\alpha_m + \beta_m} \quad \text{(EQ S2.4)}$$

$$\alpha_m = \frac{0.32(V+47.13)}{1 - \exp(-0.1(V+47.13)} \quad \text{(EQ S2.5)}$$

$$\beta_m = 0.08 * \exp(-\frac{V}{11.0}) \quad \text{(EQ S2.6)}$$

$$\frac{dh}{dt} = \frac{h_\infty - h}{\tau_h} \quad \text{(EQ S2.7)}$$

$$h_\infty = \frac{\alpha_h}{\alpha_h + \beta_h} \quad \text{(EQ S2.8)}$$

$$\tau_h = \frac{1.0}{\alpha_h + \beta_h} \quad \text{(EQ S2.9)}$$

$$\alpha_h = \begin{cases} 0.135 \exp\left(-\frac{V+80.0}{6.8}\right), & \text{if } V < -40 \text{ mV} \\ 0.0 & \text{, otherwise} \end{cases} \quad \text{(EQ S2.10)}$$

$$\beta_h = \begin{cases} 3.56\exp(0.079V) + 3.1\times 10^5 \exp(0.35V), & \text{if } V < -40 \text{ mV} \\ \frac{1.0}{0.13\left(1+\exp\left(-\frac{V+10.66}{11.1}\right)\right)} & \text{, otherwise} \end{cases}$$ (EQ S2.11)

$$\frac{dj}{dt} = \frac{j_\infty - j}{\tau_j}$$ (EQ S2.12)

$$j_\infty = \frac{\alpha_j}{\alpha_j + \beta_j}$$ (EQ S2.13)

$$\tau_j = \frac{1.0}{\alpha_j + \beta_j}$$ (EQ S2.14)

$$\alpha_j = \begin{cases} \frac{-(1.27\times 10^5 \exp(0.2444V) + 3.474\times 10^{-5}\exp(-0.04391V))(V+37.78)}{1+\exp(0.311(V+79.23))} & \text{, if } V < -40 \text{ mV} \\ 0.0 & \text{, otherwise} \end{cases}$$

(EQ S2.15)

$$\beta_j = \begin{cases} \frac{0.1212\exp(-0.01052V)}{1+\exp(-0.1378(V+40.14))} & \text{, if } V < -40 \text{ mV} \\ \frac{0.3\exp(2.35\times 10^{-7}V)}{1+\exp(-0.1(V+32))} & \text{, otherwise} \end{cases}$$ (EQ S2.16)

2.1.2 Late Na^+ Current: I_{NaL}

$$I_{NaL} = X_{NaL} g_{NaL} m_L^3 h_L (V - E_{Na})$$ (EQ S2.17)

$$\frac{dm_L}{dt} = \frac{m_{L,\infty} - m_L}{\tau_{m_L}}$$ (EQ S2.18)

$$m_{L,\infty} = \frac{\alpha_{m_L}}{\alpha_{m_L} + \beta_{m_L}}$$ (EQ S2.19)

$$\tau_{m_L} = \frac{1.0}{\alpha_{m_L} + \beta_{m_L}}$$ (EQ S2.20)

$$\alpha_{m_L} = \frac{0.32(V+47.13)}{1-\exp(-0.1(V+47.13))}$$ (EQ S2.21)

$$\beta_{m_L} = 0.08\exp\left(-\frac{V}{11}\right)$$ (EQ

S2.22)

$$\frac{dh_L}{dt} = \frac{h_{L,\infty} - h_L}{\tau_{h_L}}$$ (EQ

S2.23)

$$h_{L,\infty} = 1/(1 + \exp\left(\frac{V+91.0}{6.1}\right))$$ (EQ

S2.24)

$$\tau_{h_L} = \begin{cases} 600.0, \text{if in the CTL condition} \\ 804.0, \text{if in the HF condition} \end{cases}$$ (EQ

S2.25)

2.1.3 Inward Rectifier K⁺ Current: I_{K1}

$$I_{K1} = X_{K1} g_{K1} x_{K1} (V - E_K)$$ (EQ

S2.26)

$$g_{K1} = \begin{cases} 0.5\sqrt{\frac{[K^+]_o}{5.4}}, \text{if in the CTL condition} \\ \begin{cases} 0.2945\sqrt{\frac{[K^+]_o}{5.4}}, \text{if Endo cell in the HF condition} \\ 0.2965\sqrt{\frac{[K^+]_o}{5.4}}, \text{if M cell in the HF condition} \\ 0.2955\sqrt{\frac{[K^+]_o}{5.4}}, \text{if Epi cell in the HF condition} \end{cases} \end{cases}$$ (EQ

S2.27)

$$x_{K1} = \frac{\alpha_{K1}}{\alpha_{K1} + \beta_{K1}}$$ (EQ

S2.28)

$$\alpha_{K1} = \frac{1.02}{1 + \exp(0.2385(V - 59.215 - E_K))}$$ (EQ

S2.29)

$$\beta_{K1} = \frac{0.49124 \exp(0.08032(V + 5.476 - E_K)) + \exp(0.06175(V - 594.31 - E_K))}{1 + \exp(-0.5143(V + 4.753 - E_K))}$$ (EQ

S2.30)

2.1.4 Transient Outward K⁺ Current: I_{to1}

$$I_{to1} = X_{to1} g_{to1} a^3 i i_2 r_{to1} (V - E_k)$$ (EQ

S2.31)

$$\frac{da}{dt} = \frac{a_\infty - a}{\tau_a}$$ (EQ

S2.32)

$$a_\infty = \frac{\alpha_a}{\alpha_a + \beta_a}$$ (EQ

S2.33)

$$\tau_a = \frac{1.0}{\alpha_a + \beta_a}$$ (EQ S2.34)

$$\alpha_a = 25.0 \frac{\exp\left(\frac{V-40}{25}\right)}{1+\exp\left(\frac{V-40}{25}\right)}$$ (EQ S2.35)

$$\beta_a = 25.0 \frac{\exp\left(-\frac{(V+90)}{25}\right)}{1+\exp\left(-\frac{V+90}{25}\right)}$$ (EQ S2.36)

$$\frac{di}{dt} = \frac{i_\infty - i}{\tau_i}$$ (EQ S2.37)

$$i_\infty = \frac{\alpha_i}{\alpha_i + \beta_i}$$ (EQ S2.38)

$$\tau_i = \frac{1.0}{\alpha_i + \beta_i}$$ (EQ S2.39)

$$\alpha_i = \frac{0.03}{1+\exp\left(\frac{V+60}{5}\right)}$$ (EQ S2.40)

$$\beta_i = 0.2 \frac{\exp\left(\frac{V+25}{5}\right)}{1+\exp\left(\frac{V+25}{5}\right)}$$ (EQ S2.41)

$$\frac{di_2}{dt} = \frac{i_{2,\infty} - i_2}{\tau_{i_2}}$$ (EQ S2.42)

$$i_{2,\infty} = \frac{\alpha_{i_2}}{\alpha_{i_2} + \beta_{i_2}}$$ (EQ S2.43)

$$\tau_{i_2} = \frac{1.0}{\alpha_{i_2} + \beta_{i_2}}$$ (EQ S2.44)

$$\alpha_{i_2} = \begin{cases} \frac{0.0026}{1+\exp\left(\frac{V+61}{5}\right)}, \text{Endo cell} \\ \frac{0.00225}{1+\exp\left(\frac{V+60}{5}\right)}, \text{M cell} \\ \frac{0.0039}{1+\exp\left(\frac{V+63}{5}\right)}, \text{Epi cell} \end{cases}$$ (EQ S2.45)

$$\beta_{i_2} = 0.1\frac{\exp\left(\frac{V+25}{5}\right)}{1+\exp\left(\frac{V+25}{5}\right)}$$ (EQ S2.46)

$$r_{to1} = \exp\left(\frac{V}{300}\right)$$ (EQ S2.47)

2.1.5 Fast Delayed Rectifier K$^+$ Current : I_{Kr}

$$I_{Kr} = g_{Kr} x_r r_{r,\infty}(V - E_K)$$ (EQ S2.48)

$$g_{Kr} = 0.0138542\sqrt{\frac{[K^+]_o}{5.4}}$$ (EQ S2.49)

$$r_{r,\infty} = \frac{1.0}{1+\exp\left(\frac{V+10}{15.4}\right)}$$ (EQ S2.50)

$$\frac{dx_r}{dt} = \frac{x_{r,\infty}-x_r}{\tau_{x_r}}$$ (EQ S2.51)

$$x_{r,\infty} = \frac{1.0}{1+\exp\left(-\frac{V+10.085}{4.25}\right)}$$ (EQ S2.52)

$$\tau_{x_r} = \begin{cases} \dfrac{1}{\frac{0.0006(V-1.7384)}{1-\exp(-0.136(V-1.7384))}+\frac{0.0003(V+38.3608)}{\exp(0.1522(V+38.3608))-1}}, \text{if Endo cell} \\ \dfrac{2}{\frac{0.0006(V-1.7384)}{1-\exp(-0.136(V-1.7384))}+\frac{0.0003(V+38.3608)}{\exp(0.1522(V+38.3608))-1}}, \text{if M cell} \\ \dfrac{1}{\frac{0.0006(V-1.7384)}{1-\exp(-0.136(V-1.7384))}+\frac{0.0003(V+38.3608)}{\exp(0.1522(V+38.3608))-1}}, \text{if Epi cell} \end{cases}$$ (EQ S2.53)

2.1.6 Slow Delayed Rectifier K$^+$ Current : I_{Ks}

$$I_{Ks} = X_{Ks} g_{Ks} x_{s1} x_{s2}(V - E_{Ks})$$ (EQ S2.54)

$$g_{Ks} = \begin{cases} 0.0248975\left(1+\dfrac{0.6}{1+\left(\frac{0.000038}{[Ca^{2+}]_i}\right)^{1.4}}\right), \text{if in the CTL condition} \\ 0.00746925\left(1+\dfrac{0.6}{1+\left(\frac{0.000038}{[Ca^{2+}]_i}\right)^{1.4}}\right), \text{if in the HF condition} \end{cases}$$ (EQ S2.55)

$$\frac{dx_{s1}}{dt} = \frac{x_{s,\infty}-x_{s1}}{\tau_{x_{s1}}}$$ (EQ S2.56)

$$\frac{dx_{s2}}{dt} = \frac{x_{s,\infty} - x_{s2}}{\tau_{x_{s2}}}$$ (EQ S2.57)

$$x_{s,\infty} = \frac{1.0}{1 + \exp\left(-\frac{V - 10.5}{24.7}\right)}$$ (EQ S2.58)

$$\tau_{x_{s1}} = \frac{1}{\frac{0.0000761(V+44.6)}{1 - \exp(-9.97(V+44.6))} + \frac{0.00036(V-0.55)}{\exp(0.128(V-0.55)) - 1}}$$ (EQ S2.59)

$$\tau_{x_{s2}} = 2\tau_{x_{s1}}$$ (EQ S2.60)

2.1.7 L-type Ca^{2+} Current : I_{CaL}

$$I_{CaL} = d^{d_2} f f_2 f_{Ca} f_{Ca2} \overline{i_{Ca}}$$ (EQ S2.61)

$$\overline{i_{Ca}} = p_{Ca} z_{Ca}^2 \frac{(V-15)F^2}{RT} \frac{\gamma_{Ca_i}[Ca^{2+}]_r \exp\left(\frac{z_{Ca}F(V-15)}{RT}\right) - \gamma_{Ca_o}[Ca^{2+}]_o}{\exp\left(\frac{z_{Ca}F(V-15)}{RT}\right) - 1}$$ (EQ S2.62)

$$\frac{dd}{dt} = \frac{d_\infty - d}{\tau_d}$$ (EQ S2.63)

$$d_\infty = \begin{cases} \frac{1.0}{1 + \exp\left(-\frac{V - 4.0}{6.74}\right)}, \text{if in the CTL condition} \\ \frac{1.0}{1 + \exp\left(-\frac{V + 3.64}{6.74}\right)}, \text{if in the HF condition} \end{cases}$$ (EQ S2.64)

$$\tau_d = \frac{0.59 + 0.8\exp(0.052(V+13))}{1 + \exp(0.132(V+13))}$$ (EQ S2.65)

$$\frac{df}{dt} = \frac{f_\infty - f}{\tau_f}$$ (EQ S2.66)

$$f_\infty = 0.3 + \frac{0.7}{1 + \exp\left(\frac{V + 17.12}{7.0}\right)}$$ (EQ S2.67)

$$\tau_f = \frac{1.0}{0.2411\exp(-(0.045(V - 9.6914))^2 + 0.0529)}$$ (EQ S2.68)

$$\frac{df_2}{dt} = \frac{f_{2,\infty} - f_2}{\tau_{f_2}}$$ (EQ S2.69)

$$f_{2,\infty} = 0.23 + \frac{0.77}{1+\exp\left(\frac{V+17.12}{7.0}\right)}$$ (EQ S2.70)

$$\tau_{f_2} = \frac{1.0}{0.0423\exp\left(-(0.059(V-18.5726))^2+0.0054\right)}$$ (EQ S2.71)

$$\frac{df_{Ca}}{dt} = \frac{f_{Ca,\infty}-f_{Ca}}{\tau_{f_{Ca}}}$$ (EQ S2.72)

$$f_{Ca,\infty} = \frac{0.3}{1-\frac{I_{CaL}}{0.05}} + \frac{0.55}{1+\frac{[Ca^{2+}]_r}{0.003}} + 0.15$$ (EQ S2.73)

$$\tau_{f_{Ca}} = 0.5 + \frac{10.0Ca_{MK,act}}{Ca_{MK,act}+k_{m,Ca,MK}} + \frac{1.0}{1+\frac{[Ca^{2+}]_r}{0.003}}$$ (EQ S2.74)

$$\frac{df_{Ca2}}{dt} = \frac{f_{Ca2,\infty}-f_{Ca2}}{\tau_{f_{Ca2}}}$$ (EQ S2.75)

$$f_{Ca2,\infty} = \frac{1.0}{1-\frac{I_{CaL}}{0.01}}$$ (EQ S2.76)

$$\tau_{f_{Ca2}} = 125.0 + \frac{300.0}{1+\exp\left(-\frac{I_{CaL}+0.175}{0.04}\right)}$$ (EQ S2.77)

$$\frac{dd_2}{dt} = \frac{d_{2,\infty}-d_2}{\tau_{d_2}}$$ (EQ S2.78)

$$d_{2,\infty} = 9 - \frac{8}{1+\exp\left(-\frac{V+65}{3.4}\right)}$$ (EQ S2.79)

$$\tau_{d_2} = 10.0$$ (EQ S2.80)

2.1.8 Plateau Current : I_{Kp}

$$I_{Kp} = g_{Kp}k_p(V - E_K)$$ (EQ S2.81)

$$k_p = \frac{1.0}{1+\exp\left(\frac{7.488-V}{5.98}\right)}$$ (EQ S2.82)

2.1.9 Ca^{2+}-dependent Transient Outward Cl^- Current : I_{to2}

$$I_{to2} = \overline{i_{to2}}a_{to1}$$ (EQ

S2.83)

$$\overline{i_{to2}} = p_{Cl} z_{Cl}^2 \frac{VF^2}{RT} \frac{[Cl^-]_i - [Cl^-]_o \exp\left(-\frac{z_{Cl}VF}{RT}\right)}{1 - \exp\left(-\frac{z_{Cl}VF}{RT}\right)}$$ (EQ S2.84)

$$\frac{da_{to2}}{dt} = \frac{a_{to2,\infty} - a_{to2}}{\tau_{to2}}$$ (EQ S2.85)

$$a_{to2,\infty} = \frac{1.0}{1 + \frac{k_{m,to2}}{[Ca^{2+}]_r}}$$ (EQ S2.86)

$$\tau_{a_{to2}} = 1.0$$ (EQ S2.87)

2.1.10 Na^{+}-Ca^{2+} Exchange Current : NCX

$$NCX = \frac{X_{NCX} i_{NCX,max} [Na^+]_i^3 [Ca^{2+}]_o \exp\left(\frac{0.35VF}{RT}\right) - 1.5[Na^+]_o^3 [Ca^{2+}]_i \exp\left(-\frac{0.65VF}{RT}\right))}{(1 + (\frac{k_{m,Ca,act}}{1.5[Ca^{2+}]_i})^2)(1 + k_{sat} \exp\left(-\frac{0.65VF}{RT}\right))(d_{NCX1} + d_{NCX2})}$$ (EQ S2.88)

$$d_{NCX1} = k_{m,Ca_o}[Na^+]_i^3 + 1.5k_{m,Na_o}^3 [Ca^{2+}]_i + k_{m,Na_i,1}^3 [Ca^{2+}]_o (1 + \frac{1.5[Ca^{2+}]_i}{k_{m,Ca_i}})$$

(EQ S2.89)

$$d_{NCX2} = k_{m,Ca_i}[Na^+]_o^3 (1 + (\frac{[Na^+]_i}{k_{m,Na_i,1}})^3) + [Na^+]_i^3 [Ca^{2+}]_o + 1.5[Na^+]_o^3 [Ca^{2+}]_i$$

(EQ S2.90)

2.1.11 Background Cl^{-} Current : I_{Clb}

$$I_{Clb} = g_{Clb}(V - E_{Cl})$$ (EQ S2.91)

2.1.12 Background Ca^{2+} Current : I_{Cab}

$$I_{Cab} = p_{Ca} z_{Ca}^2 \frac{VF^2}{RT} \frac{\gamma_{Ca_i}[Ca^{2+}]_i \exp\left(z_{Ca}\frac{VF}{RT}\right) - \gamma_{Ca_o}[Ca^{2+}]_o}{\exp\left(z_{Ca}\frac{VF}{RT}\right) - 1}$$ (EQ S2.92)

2.1.13 Na^{+} / K^{+} Pump Current : I_{NaK}

$$I_{NaK} = \overline{g_{NaK}} f_{NaK} \frac{1.0}{1 + (\frac{k_{m,Na_i,2}}{[Na^+]_i})^2} \frac{[K^+]_o}{[K^+]_o + k_{m,K_o}}$$ (EQ S2.93)

$$f_{NaK} = \frac{1.0}{1.0 + 0.1245 \exp\left(-\frac{0.1VF}{RT}\right) + 0.0365\sigma \exp\left(-\frac{VF}{RT}\right)}$$ (EQ

S2.94)

$$\sigma = \frac{1}{7}(\exp\left(\frac{[Na^+]_o}{67.3}\right) - 1)$$ (EQ S2.95)

2.1.14 Sarcolemmal Ca^{2+} Pump Current : I_{Cap}

$$I_{Cap} = \overline{i_{Cap}}\frac{[Ca^{2+}]_i}{[Ca^{2+}]_i + k_{m,Cap}}$$ (EQ S2.96)

2.1.15 K^+ / Cl^- Co-transporter

$$CT_{K-Cl} = \overline{CT_{K-Cl}}\frac{E_K - E_{Cl}}{E_K - E_{Cl} + 87.8251}$$ (EQ S2.97)

2.1.16 Na^+ / Cl^- Co-transporter

$$CT_{Na-Cl} = \overline{CT_{Na-Cl}}\frac{(E_{Na} - E_{Cl})^4}{(E_{Na} - E_{Cl})^4 + 87.8251^4}$$ (EQ S2.98)

2.1.17 Intracellular Ion Concentrations

$$\frac{d[Na^+]_i}{dt} = -\frac{(I_{Na} + I_{NaL} + 3I_{NaK} + 3NCX)a_{cap}}{Vol_{myo}F} + CT_{Na-Cl}$$ (EQ S2.99)

$$\frac{d[K^+]_i}{dt} = -\frac{(I_{to1} + I_{K1} + I_{Kr} + I_{Ks} + I_{Kp} - 2I_{NaK})a_{cap}}{Vol_{myo}F} + CT_{K-Cl}$$ (EQ S2.100)

$$\frac{d[Cl^-]_i}{dt} = -\frac{(I_{to2} + I_{Clb})a_{cap}}{Vol_{myo}F} + CT_{Na-Cl} + CT_{K-Cl}$$ (EQ S2.101)

2.1.18 Intracellular Ca^{2+} Concentration

$$\frac{d[Ca^{2+}]_i}{dt} = -\frac{(I_{Cap} - 2NCX)a_{cap}}{z_{Ca}Vol_{myo}F} + (q_{up} - q_{leak})\frac{Vol_{nsr}}{Vol_{myo}} - q_{diff}\frac{Vol_{ss}}{Vol_{myo}}$$ (EQ S2.102)

$$d_{myo} = -k_{m,TRPN}k_{m,CMDN}[Ca^{2+}]_{tot}$$ (EQ S2.103)

$$c_{myo} = k_{m,CMDN}k_{m,TRPN} - [Ca^{2+}]_{tot}(k_{m,TRPN} + k_{m,CMDN}) + \overline{TRPN} \times k_{m,CMDN} + \overline{CMDN} \times k_{m,TRPN}$$ (EQ S2.104)

$$b_{myo} = \overline{CMDN} + \overline{TRPN} - [Ca^{2+}]_{tot} + k_{m,TRPN} + k_{m,CMDN}$$ (EQ S2.105)

$$[Ca^{2+}]_{tot} = TRPN + CMDN + d[Ca^{2+}]_i + [Ca^{2+}]_i$$ (EQ S2.106)

$$CMDN = \overline{CMDN}\left(\frac{[Ca^{2+}]_i}{[Ca^{2+}]_i + k_{m,CMDN}}\right)$$ (EQ S2.107)

$$TRPN = \overline{TRPN}\left(\frac{[Ca^{2+}]_i}{[Ca^{2+}]_i + k_{m,TRPN}}\right)$$ (EQ S2.108)

$$[Ca^{2+}]_i = \frac{2}{3}\sqrt{b_{myo}^2 - 3c_{myo}}\cos\left(\frac{1}{3}\arccos\left(\frac{9b_{myo}c_{myo} - 2b_{myo}^3 - 27d_{myo}}{2(b_{myo}^2 - 3c_{myo})^{1.5}}\right)\right) - \frac{b_{myo}}{3}$$

(EQ S2.109)

2.1.19 Ca^{2+} / Calmodulin-dependent Protein Kinase

$$Ca_{MK,act} = Ca_{MK,bound} + Ca_{MK,trap}$$ (EQ S2.110)

$$\frac{dCa_{MK,trap}}{dt} = \alpha_{Ca,MK}Ca_{MK,bound}\left(Ca_{MK,bound} + Ca_{MK,trap}\right) - \beta_{Ca,MK}Ca_{MK,trap}$$ (EQ S2.111)

$$Ca_{MK,bound} = \frac{Ca_{MK,0}(1 - Ca_{MK,trap})}{1 + \frac{k_{m,Ca,M}}{[Ca^{2+}]_r}}$$ (EQ S2.112)

2.1.20 NSR Ca^{2+} Concentration

$$\frac{d[Ca^{2+}]_{NSR}}{dt} = q_{up} - q_{leak} - q_{tr}\frac{Vol_{JSR}}{Vol_{NSR}}$$ (EQ S2.113)

2.1.21 JSR Ca^{2+} Concentration

$$\frac{d[Ca^{2+}]_{JSR}}{dt} = \frac{q_{tr} - q_{rel}}{1 + \overline{CSQN}\frac{k_{m,CSQN}}{(k_{m,CSQN} + [Ca^{2+}]_{JSR})^2}}$$ (EQ S2.114)

2.1.22 Restricted Space Ca^{2+} Concentration

$$[Ca^{2+}]_r = \frac{2}{3}\sqrt{b_1^2 - 3c_1}\cos\left(\frac{1}{3}\arccos\left(\frac{9b_1c_1 - 2b_1^3 - 27d_1}{2(b_1^2 - 3c_1)^{1.5}}\right)\right) - \frac{b_1}{3}$$ (EQ

S2.115)

$$d_1 = -k_{m,b,SR}k_{m,b,SL}[Ca^{2+}]_{r,tot}$$ (EQ

S2.116)

$$c_1 = k_{m,b,SR}k_{m,b,SL} - [Ca^{2+}]_{r,tot}\left(k_{m,b,SR} + k_{m,b,SL}\right) + \overline{b_{SR}}k_{m,b,SL} + \overline{b_{SL}}k_{m,b,SR}$$

(EQ

S2.117)

$$b_1 = \overline{b_{SR}} + \overline{b_{SL}} - [Ca^{2+}]_{r,tot} + k_{m,b,SR} + k_{m,b,SL}$$ (EQ

S2.118)

$$[Ca^{2+}]_{r,tot} = [Ca^{2+}]_r + b_{SR} + b_{SL} + d[Ca^{2+}]_r$$ (EQ

S2.119)

$$b_{SL} = \overline{b_{SL}}\left(\frac{[Ca^{2+}]_r}{[Ca^{2+}]_r + k_{m,b,SL}}\right)$$ (EQ

S2.120)

$$b_{SR} = \overline{b_{SR}}\left(\frac{[Ca^{2+}]_r}{[Ca^{2+}]_r + k_{m,b,SR}}\right)$$ (EQ

S2.121)

$$\frac{d[Ca^{2+}]_r}{dt} = -\frac{I_{CaL}a_{Cap}}{Vol_{ss}z_{Ca}F} + q_{rel}\frac{Vol_{JSR}}{Vol_{ss}} - \frac{[Ca^{2+}]_r - [Ca^{2+}]_i}{\tau_{ss}}$$ (EQ

S2.122)

2.1.23 SR Release Flux

$$q_{rel} = \overline{g_{rel}}r_o r_i([Ca^{2+}]_{JSR} - [Ca^{2+}]_r)$$ (EQ

S2.123)

$$\overline{g_{rel}} = \begin{cases} 3000v_g, \text{if in the CTL condition} \\ 1800v_g, \text{if in the HF condition} \end{cases}$$ (EQ

S2.124)

$$v_g = \frac{1.0}{1 + \exp\left(\frac{g_{CaL}\overline{i_{Ca}} + 13}{5}\right)}$$ (EQ

S2.125)

$$\frac{dr_i}{dt} = \frac{r_{i,\infty} - r_i}{\tau_{r_i}}$$ (EQ

S2.126)

$$r_{i,\infty} = \frac{1.0}{1 + \exp\left(\frac{[Ca^{2+}]_r - 0.0004 + 0.002Ca_{fac}}{0.000025}\right)}$$ (EQ

S2.127)

$$\tau_{r_i} = \frac{350 - \tau_{Ca,MK}}{1 + \exp\left(\frac{[Ca^{2+}]_r - 0.003 + 0.003Ca_{fac}}{0.0002}\right)} + 3.0 + \tau_{Ca,MK}$$ (EQ

S2.128)

$$Ca_{fac} = \frac{1.0}{1 + \exp\left(\frac{I_{CaL} + 0.05}{0.015}\right)}$$ (EQ

S2.129)

$$\tau_{Ca,MK} = \overline{\tau_{Ca,MK}} \frac{Ca_{MK,act}}{k_{m,Ca,MK}+Ca_{MK,act}}$$ (EQ

S2.130)

$$\frac{dr_o}{dt} = \frac{r_{o,\infty}-r_o}{\tau_{r_o}}$$ (EQ

S2.131)

$$r_{o,\infty} = r_{o,\infty,JSR} \frac{I_{CaL}^2}{I_{CaL}^2+1}$$ (EQ

S2.132)

$$r_{o,\infty,JSR} = \frac{[Ca^{2+}]_{JSR}^{1.9}}{[Ca^{2+}]_{JSR}^{1.9}+(\frac{49.28[Ca^{2+}]_r}{[Ca^{2+}]_r+0.0028})^{1.9}}$$ (EQ

S2.133)

$$\tau_{r_o} = 3.0$$ (EQ

S2.134)

2.1.24 SR Leak Flux

$$q_{leak} = \overline{q_{leak}} \frac{[Ca^{2+}]_{NSR}}{\overline{NSR}}$$ (EQ

S2.135)

2.1.25 SR Uptake Flux

$$q_{up} = X_{qup}(dq_{up,Ca,MK} + 1)\overline{q_{up}} \frac{[Ca^{2+}]_i}{[Ca^{2+}]_i+k_{m,up}-dk_{m,plb}}$$ (EQ

S2.136)

$$dq_{up,Ca,MK} = d\overline{q_{up,Ca,MK}} \frac{Ca_{MK,act}}{k_{m,Ca,MK}+Ca_{MK,act}}$$ (EQ

S2.137)

$$dk_{m,pld} = \overline{dk_{m,pld}} \frac{Ca_{MK,act}}{k_{m,Ca,MK}+Ca_{MK,act}}$$ (EQ

S2.138)

2.1.26 SR Transfer Flux

$$q_{tr} = \frac{[Ca^{2+}]_{NSR}-[Ca^{2+}]_{JSR}}{\tau_{tr}}$$ (EQ

S2.139)

2.1.27 Equilibrium Potentials

$$E_{Na} = \frac{RT}{F} \ln \frac{[Na^+]_o}{[Na^+]_i}$$ (EQ

S2.140)

$$E_K = \frac{RT}{F}\ln\frac{[K^+]_o}{[K^+]_i}$$ (EQ S2.141)

$$E_{Ca} = \frac{RT}{2F}\ln\frac{[Ca^{2+}]_o}{[Ca^{2+}]_i}$$ (EQ S2.142)

$$E_{Cl} = \frac{RT}{F}\ln\frac{[Cl^-]_o}{[Cl^-]_i}$$ (EQ S2.143)

$$E_{K,s} = \frac{RT}{F}\ln\frac{[K^+]_o + r_{NaK}[Na^+]_o}{[K^+]_i + r_{NaK}[Na^+]_i}$$ (EQ S2.144)

S2.2 Model Parameters

Supplementary Table S15 Model parameters of canine ventricular model

Parameters	Values	
	CTL	HF
Universal gas constant, R	8314 J/(K*kmol)	8314 J/(K*kmol)
Faraday's constant, F	96485 C/mol	96485 C/mol
Temperature, T	310 K	310 K
External Na^+ concentration, $[Na^+]_o$	140.0 mM	140.0 mM
External Ca^{2+} concentration, $[Ca^{2+}]_o$	1.8 mM	1.8 mM
External K^+ concentration, $[K^+]_o$	5.4 mM	5.4 mM
External Cl^- concentration, $[Cl^-]_o$	100.0 mM	100.0 mM
Na^+ ion valence, z_{Na}	1	1
Ca^{2+} ion valence, z_{Ca}	2	2
K^+ ion valence, z_K	1	1
Cl^- ion valence, z_{Cl}	-1	-1
Internal Na^+ activity coefficient, γ_{Na_i}	0.75	0.75
External Na^+ activity coefficient, γ_{Na_o}	0.75	0.75
Internal Ca^{2+} activity coefficient, γ_{Ca_i}	1.0	1.0
External Ca^{2+} activity coefficient, γ_{Ca_o}	0.341	0.341
Internal K^+ activity coefficient, γ_{K_i}	0.75	0.75

External K^+ activity coefficient, γ_{K_o}	0.75	0.75
Maximum conductance of I_{Na}, g_{Na}	8.25 pA/pF	5.61 pA/pF
Maximum conductance of I_{NaL}, g_{NaL}	0.0065 pA/pF	0.00845 pA/pF
Additional scaling factor for I_{NaL}, X_{NaL}	1.15 (Endo cell)<br>1.7 (M cell)<br>1.0 (Epi cell)	1.15 (Endo cell)<br>1.7 (M cell)<br>1.0 (Epi cell)
Additional scaling factor for I_{K1}, X_{K1}	0.7 (Endo cell)<br>1.1 (M cell)<br>1.0 (Epi cell)	0.7 (Endo cell)<br>1.1 (M cell)<br>1.0 (Epi cell)
Maximum conductance of I_{to1}, g_{to1}	0.19 pA/pF	0.1083 pA/pF (Endo cell)<br>0.1045 pA/pF (M cell)<br>0.1083 pA/pF (Epi cell)
Additional scaling factor for I_{to1}, X_{to1}	0.5 (Endo cell)<br>0.95 (M cell)<br>1.0 (Epi cell)	0.5 (Endo cell)<br>0.95 (M cell)<br>1.0 (Epi cell)
Additional scaling factor for I_{Ks}, X_{Ks}	0.59 (Endo cell)<br>0.68 (M cell)<br>1.0 (Epi cell)	0.59 (Endo cell)<br>0.68 (M cell)<br>1.0 (Epi cell)
Half-saturation coefficient of CaM, $k_{m,Ca,M}$	0.0015 mM	0.0015 mM
Membrane permeability to Ca^{2+}, p_{Ca}	0.000243 cm/s	0.000243 cm/s

Maximum conductance of I_{Kp}, g_{Kp}	0.00276 pA/pF	0.00276 pA/pF
Constant for low affinity binding of subspace Ca^{2+}, $k_{m,to2}$	0.1502 mM	0.1502 mM
Membrane permeability to Cl^-, p_{cl}	0.0000004 cm/s	0.0000004 cm/s
Additional scaling factor for the NCX, X_{NCX}	0.9 (Endo cell)<br>1.3 (M cell)<br>1.0 (Epi cell)	0.9 (Endo cell)<br>1.3 (M cell)<br>1.0 (Epi cell)
Maximal NCX, $i_{NCX,max}$	4.5 pA/pF	5.4 pA/pF
Half-saturation concentration for $[Ca^{2+}]_i$ activation, $k_{m,Ca,act}$	0.000125 mM	0.000125 mM
Half-saturation concentration for$[Na^+]_i$, $k_{m,Na_i,1}$	12.3 mM	12.3 mM
Half-saturation concentration for $[Na^+]_o$, k_{m,Na_o}	87.5 mM	87.5 mM
Half-saturation concentration for $[Ca^{2+}]_i$, k_{m,Ca_i}	0.0036 mM	0.0036 mM
Half-saturation concentration for $[Ca^{2+}]_o$, k_{m,Ca_o}	1.3 mM	1.3 mM
saturation factor for the NCX at negative potentials, k_{sat}	0.27	0.27
Maximum conductance of I_{Clb}, g_{Clb}	0.000225 pA/pF	0.000225 pA/pF
Maximum I_{NaK}, $\overline{g_{NaK}}$	0.61875 pA/pF	0.358875 pA/pF
Half-saturation concentration for $[Na^+]_i$, $k_{m,Na_i,2}$	10.0 mM	10.0 mM

Half-saturation concentration for $[K^+]_o$, k_{m,K_o}	1.5 mM	1.5 mM
Maximum I_{Cap}, $\overline{i_{Cap}}$	0.0575 pA/pF	0.0575 pA/pF
half saturation concentration for I_{Cap}, $k_{m,Cap}$	0.0005 mM	0.0005 mM
Maximum K^+ / Cl^- transport, $\overline{CT_{K-Cl}}$	7.0756 × 10^{-6} mM/ms	7.0756 × 10^{-6} mM/ms
Maximum Na^+ / Cl^- transport, $\overline{CT_{Na-Cl}}$	9.8443 × 10^{-6} mM/ms	9.8443 × 10^{-6} mM/ms
Factor for modulation of time constant for inactivation of q_{rel} by CaMKII, maximum, $\overline{\tau_{Ca,MK}}$	10.0	10.0
Maximum leak from the NSR to myoplasm, $\overline{q_{leak}}$	0.004375 mM/ms	0.002861 mM/ms
Maximum calcium in the NSR, $\overline{NSR}$	15.0 mM	15.0 mM
Maximum modulation of q_{up} (maximum flux) by CAMK, $d\overline{q_{up,Ca,MK}}$	0.75	0.75
Maximum modulation of phospholamban (half-saturation) by CAMK, $\overline{dk_{m,pld}}$	0.00017	0.00017
Half-saturation concentration of q_{up}, $k_{m,up}$	0.00092 mmol	0.00092 mmol
Maximum uptake from myoplasm to the NSR, $\overline{q_{up}}$	0.0035 mM/ms	0.0035 mM/ms
Time constant of transfer from the NSR to the JSR, τ_{tr}	120.0 ms	120.0 ms

Maximum Ca^{2+} binding by anionic binding sites in the subspace, $\overline{b_{SR}}$	0.047 mM	0.047 mM
Half-saturation coefficient of anionic binding sites in the subspace, $k_{m,b,SR}$	0.00087 mM	0.00087 mM
Maximum Ca^{2+} binding by sarcolemmal binding sites in the subspace, $\overline{b_{SL}}$	1.124 mM	1.124 mM
Half-saturation coefficient of sarcolemmal binding sites in the subspace, $k_{m,b,SL}$	0.0087 mM	0.0087 mM
Time constant for diffusion between restricted space and myoplasm, τ_{ss}	0.2 ms	0.2 ms
Maximum Ca^{2+} buffered by calsequestrin, $\overline{CSQN}$	10.0 mM	10.0 mM
Equilibrium constant for calsequestrin buffering, $k_{m,CSQN}$	0.8 mM	0.8 mM
Maximum Ca^{2+} buffered by calmodulin, $\overline{CMDN}$	0.05 mM	0.05 mM
Equilibrium constant for calmodulin buffering, $k_{m,CMDN}$	0.00238 mM	0.00238 mM
Maximum Ca^{2+} buffered by troponin, $\overline{TRPN}$	0.07 mM	0.07 mM
Equilibrium constant for troponin buffering, $k_{m,TRPN}$	0.0005 mM	0.0005 mM
Fraction of active CaMKII binding sites at equilibrium, $Ca_{MK,0}$	0.05	0.05
Phosphorylation rate of CaMKII, $\alpha_{Ca,MK}$	0.05 1/ms	0.05 1/ms

Dephosphorylation rate of CaMKII, $\beta_{Ca,MK}$	0.00068 1/ms	0.00068 1/ms
Half-saturation coefficient of CaMKII, $k_{m,Ca,MK}$	0.15	0.15

S2.3 Initial Conditions

Supplementary Table S16 Initial conditions of canine ventricular model

Parameters	Values					
	CTL			HF		
	Endo	M	Epi	Endo	M	Epi
V (mV)	-86.58569312	-86.8077503	-86.80487879	-84.81164179	-85.6642716	-85.49414056
m	0.00118599	0.00114271	0.00114326	0.00159470	0.00138343	0.00142326
h	0.98940209	0.98991589	0.98990942	0.98422786	0.98696317	0.98645855
j	0.99315865	0.99346188	0.99345807	0.99001891	0.99167774	0.99137606
m_L	0.00118599	0.00114271	0.00114326	0.00159470	0.00138343	0.00142326
h_L	0.26949321	0.27925680	0.28228530	0.15907263	0.17210081	0.17436263
d	0.00000146	0.00000141	0.00000141	0.00000589	0.00000519	0.00000532
f	0.99996569	0.99996676	0.99996675	0.99995552	0.99996066	0.99995970

f_2	0.98801493	0.98686675	0.98755968	0.98415007	0.97601442	0.97971359
f_{Ca}	0.98137282	0.98318021	0.98205553	0.97406793	0.97787296	0.97457746
f_{Ca2}	0.85424944	0.85176758	0.86006204	0.79648333	0.74959761	0.76430876
a	0.01330832	0.01313142	0.01313369	0.01481878	0.01407079	0.01421669
i	0.99997000	0.99997131	0.99997130	0.99995638	0.99996313	0.99996195
i_2	0.83139091	0.78689502	0.93249775	0.76972517	0.67977975	0.86345914
x_r	0.00002964	0.00300878	0.00002468	0.00024582	0.01258439	0.00039256
x_{s1}	0.01925693	0.01908716	0.01908931	0.02072572	0.02002636	0.02016081
x_{s2}	0.01926522	0.01909444	0.01909584	0.02084367	0.02017819	0.02028892
a_{to2}	0.00069956	0.00062959	0.00067308	0.00098572	0.00083497	0.00096519
I_{CaL} (pA/pF)	0.0	0.0	0.0	0.0	0.0	0.0
d_2	8.98602844	8.98691132	8.98690031	8.97633167	8.98159020	8.98064873
r_i	0.86318689	0.86113596	0.86434147	0.76352235	0.73510793	0.73408536
r_o	0.0	0.0	0.0	0.0	0.0	0.0
$[Ca^{2+}]_r$ (mM)	0.00010514	0.00009461	0.00010115	0.00014808	0.00012540	0.00014498

$[Ca^{2+}]_{JSR}$ (mM)	1.33664834	1.25427883	1.29016444	0.39894281	0.39390683	0.41232408
$[Ca^{2+}]_{NSR}$ (mM)	1.37501392	1.29307339	1.32970299	0.43576094	0.42941037	0.45074869
$[Na^{+}]_{i}$ (mM)	9.91463567	9.99497497	9.95420397	10.63287853	10.67340751	10.61679596
$[K^{+}]_{i}$ (mM)	141.93271011	141.90941982	141.90082197	141.62699269	141.63520395	141.63039441
$[Cl^{-}]_{i}$ (mM)	18.90479946	18.90484810	18.90376127	18.90882301	18.91174430	18.91089655
$[Ca^{2+}]_{i}$ (mM)	0.00010471	0.00009423	0.00010073	0.00014409	0.00012138	0.00014061
$Ca_{MK,act}$	0.01330499	0.01234450	0.01235062	0.02761650	0.02587044	0.02900971
$Ca_{MK,trap}$	0.01006297	0.00940574	0.00922102	0.02322835	0.02209827	0.02471193

Supplementary S3 Canine Purkinje Fiber Cell Model

S3.1 Model Equations

3.1.1 Fast Na^{+} Current : I_{Na}

$$I_{Na} = g_{Na} m^3 hj(V - E_{Na}) \quad \text{(EQ S3.1)}$$

$$\frac{dm}{dt} = \frac{m_\infty - m}{\tau_m} \quad \text{(EQ S3.2)}$$

$$m_\infty = \frac{\alpha_m}{\alpha_m + \beta_m} \quad \text{(EQ S3.3)}$$

$$\tau_m = \frac{1.0}{\alpha_m + \beta_m} \quad \text{(EQ S3.4)}$$

$$\alpha_m = \frac{0.32(V + 57.13)}{1 - \exp(-0.1(V + 57.13))} \quad \text{(EQ S3.5)}$$

$$\beta_m = 0.08 * \exp\left(-\frac{V + 10}{11.0}\right) \quad \text{(EQ S3.6)}$$

$$\frac{dh}{dt} = \frac{h_\infty - h}{\tau_h} \quad \text{(EQ S3.7)}$$

$$h_\infty = \frac{\alpha_h}{\alpha_h+\beta_h}$$ (EQ S3.8)

$$\tau_h = \frac{1.0}{\alpha_h+\beta_h}$$ (EQ S3.9)

$$\alpha_h = \begin{cases} 0.135\exp\left(-\frac{V+80.0}{6.8}\right), & \text{if } V < -40\text{ mV} \\ 0.0, & \text{otherwise} \end{cases}$$ (EQ S3.10)

$$\beta_h = \begin{cases} 3.56\exp(0.079V) + 3.1\times10^5\exp(0.35V), & \text{if } V < -40\text{ mV} \\ \frac{1.0}{0.13(1+\exp\left(-\frac{V+0.66}{5.0}\right))}, & \text{otherwise} \end{cases}$$ (EQ S3.11)

$$\frac{dj}{dt} = \frac{j_\infty - j}{\tau_j}$$ (EQ S3.12)

$$j_\infty = \frac{\alpha_j}{\alpha_j+\beta_j}$$ (EQ S3.13)

$$\tau_j = \frac{1.0}{\alpha_j+\beta_j}$$ (EQ S3.14)

$$\alpha_j = \begin{cases} \frac{-(1.27\times10^5\exp(0.2444V) + 3.474\times10^{-5}\exp(-0.04391V))(V+37.78)}{1+\exp(0.311(V+79.23))}, & \text{if } V < -40\text{ mV} \\ 0.0, & \text{otherwise} \end{cases}$$ (EQ S3.15)

$$\beta_j = \begin{cases} \frac{0.1212\exp(-0.01052V)}{1+\exp(-0.1378(V+40.14))}, & \text{if } V < -40\text{ mV} \\ \frac{0.3\exp(2.35\times10^{-7}V)}{1+\exp(-0.1(V+32))}, & \text{otherwise} \end{cases}$$ (EQ S3.16)

3.1.2 Late Na$^+$ Current : I_{NaL}

$$I_{NaL} = g_{NaL} m_L^3 h_L (V - E_{Na})$$ (EQ S3.17)

$$\frac{dm_L}{dt} = \frac{m_{L,\infty} - m_L}{\tau_{m_L}}$$ (EQ S3.18)

$$m_{L,\infty} = \frac{\alpha_{m_L}}{\alpha_{m_L}+\beta_{m_L}}$$ (EQ S3.19)

$$\tau_{m_L} = \frac{1.0}{\alpha_{m_L}+\beta_{m_L}}$$ (EQ

S3.20)

$$\alpha_{m_L} = \frac{0.32(V+47.13)}{1-\exp(-0.1(V+47.13))}$$ (EQ S3.21)

$$\beta_{m_L} = 0.08\exp\left(-\frac{V}{11}\right)$$ (EQ S3.22)

$$\frac{dh_L}{dt} = \frac{h_{L,\infty}-h_L}{\tau_{h_L}}$$ (EQ S3.23)

$$h_{L,\infty} = 1/\left(1 + \exp\left(\frac{V+69.0}{6.1}\right)\right)$$ (EQ S3.24)

$$\tau_{h_L} = 175 + \frac{125}{1+\exp\left(-\frac{V+25}{6.0}\right)}$$ (EQ S3.25)

3.1.3 Inward Rectifier K$^+$ Current : I_{K1}

$$I_{K1} = G_{K1}(V - E_K)$$ (EQ S3.26)

$$G_{K1} = \begin{cases} g_{K1}\dfrac{42.028}{1+\exp\left(\frac{V+57.2332-E_K}{14.33}\right)}, \text{if in the CTL condition} \\ g_{K1}\dfrac{7.96}{1+\exp\left(\frac{V+39.7332-E_K}{14.9969}\right)}, \text{if in the HF condition} \end{cases}$$ (EQ S3.27)

$$g_{K1} = 0.5\sqrt{\frac{[K^+]_o}{5.4}}$$ (EQ S3.28)

3.1.4 Transient Outward K$^+$ Current : I_{to1}

$$I_{to1} = X_{to1}g_{to1}a(0.8i + 0.2i_2)r_{to1}(V - E_k)$$ (EQ S3.29)

$$\frac{da}{dt} = \frac{a_\infty - a}{\tau_a}$$ (EQ S3.30)

$$a_\infty = \frac{\alpha_a}{\alpha_a+\beta_a}$$ (EQ S3.31)

$$\tau_a = \frac{1.0}{\alpha_a+\beta_a}$$ (EQ S3.32)

$$\alpha_a = \begin{cases} 25.0\,\dfrac{\exp\left(\frac{V-74}{20}\right)}{1+\exp\left(\frac{V-74}{20}\right)}, \text{if in the CTL condition} \\ 25.0\,\dfrac{\exp\left(\frac{V-75}{19}\right)}{1+\exp\left(\frac{V-75}{19}\right)}, \text{if in the HF condition} \end{cases}$$ (EQ

S3.33)

$$\beta_a = \begin{cases} 25.0\,\dfrac{\exp\left(-\frac{(V+56)}{20}\right)}{1+\exp\left(-\frac{V+56}{20}\right)}, \text{if in the CTL condition} \\ 25.0\,\dfrac{\exp\left(-\frac{(V+55)}{20}\right)}{1+\exp\left(-\frac{V+55}{20}\right)}, \text{if in the HF condition} \end{cases}$$ (EQ

S3.34)

$$\frac{di}{dt} = \frac{i_\infty - i}{\tau_i}$$ (EQ

S3.35)

$$i_\infty = \frac{\alpha_i}{\alpha_i + \beta_i}$$ (EQ

S3.36)

$$\tau_i = \begin{cases} 4.1485 + \dfrac{19.1293}{1+\exp\left(\frac{V+4.2858}{12.5734}\right)}, \text{if in the CTL condition} \\ 4.2125 + \dfrac{16.7356}{1+\exp\left(\frac{V+5.2610}{13.7592}\right)}, \text{if in the HF condition} \end{cases}$$ (EQ

S3.37)

$$\alpha_i = \frac{0.00442}{1+\exp\left(\frac{V+26}{10}\right)}$$ (EQ

S3.38)

$$\beta_i = \begin{cases} 0.05\,\dfrac{\exp\left(\frac{V-10}{15}\right)}{1+\exp\left(\frac{V-10}{15}\right)}, \text{if in the CTL condition} \\ 0.06\,\dfrac{\exp\left(\frac{V-10}{15}\right)}{1+\exp\left(\frac{V-10}{15}\right)}, \text{if in the HF condition} \end{cases}$$ (EQ

S3.39)

$$\frac{di_2}{dt} = \frac{i_{2,\infty} - i_2}{\tau_{i_2}}$$ (EQ

S3.40)

$$i_{2,\infty} = \frac{\alpha_{i_2}}{\alpha_{i_2} + \beta_{i_2}}$$ (EQ

S3.41)

$$\tau_{i_2} = \begin{cases} 21.5 + \dfrac{30.0}{1+\exp\left(\frac{V-25.0}{10.0}\right)}, \text{if in the CTL condition} \\ 24.6115 + \dfrac{26.8745}{1+\exp\left(\frac{V-25.8301}{7.3637}\right)}, \text{if in the HF condition} \end{cases}$$ (EQ

S3.42)

$$\alpha_{i_2} = \alpha_i \quad \text{(EQ S3.43)}$$

$$\beta_{i_2} = \beta_i \quad \text{(EQ S3.44)}$$

$$r_{to1} = 1.0 \quad \text{(EQ S3.45)}$$

3.1.5 Fast Delayed Rectifier K$^+$ Current : I_{Kr}

$$I_{Kr} = g_{Kr} x_r r_{r,\infty}(V - E_K) \quad \text{(EQ S3.46)}$$

$$g_{Kr} = \begin{cases} 0.0340072\sqrt{\frac{[K^+]_o}{5.4}}, \text{if in the CTL condition} \\ 0.0307323\sqrt{\frac{[K^+]_o}{5.4}}, \text{if in the HF condition} \end{cases} \quad \text{(EQ S3.47)}$$

$$r_{r,\infty} = \frac{1.0}{1+\exp\left(\frac{V-5.4}{20.4}\right)} \quad \text{(EQ S3.48)}$$

$$\frac{dx_r}{dt} = \frac{x_{r,\infty} - x_r}{\tau_{x_r}} \quad \text{(EQ S3.49)}$$

$$x_{r,\infty} = \frac{1.0}{1+\exp\left(-\frac{V+0.085}{12.25}\right)} \quad \text{(EQ S3.50)}$$

$$\tau_{x_r} = 100.0 + \frac{900.0}{1+\exp\left(\frac{V}{5.0}\right)} \quad \text{(EQ S3.51)}$$

3.1.6 Slow Delayed Rectifier K$^+$ Current : I_{Ks}

$$I_{Ks} = X_{Ks} g_{Ks} x_{s1} x_{s2}(V - E_{Ks}) \quad \text{(EQ S3.52)}$$

$$g_{Ks} = \begin{cases} 0.02240775\left(1 + \frac{0.6}{1+\left(\frac{0.000038}{[Ca^{2+}]_i}\right)^{1.4}}\right), \text{if in the CTL condition} \\ 0.02113051\left(1 + \frac{0.6}{1+\left(\frac{0.000038}{[Ca^{2+}]_i}\right)^{1.4}}\right), \text{if in the HF condition} \end{cases} \quad \text{(EQ S3.53)}$$

$$\frac{dx_{s1}}{dt} = \frac{x_{s,\infty} - x_{s1}}{\tau_{x_{s1}}} \quad \text{(EQ S3.54)}$$

$$\frac{dx_{s2}}{dt} = \frac{x_{s,\infty} - x_{s2}}{\tau_{x_{s2}}} \quad \text{(EQ}$$

S3.55)

$$x_{s,\infty} = \begin{cases} \frac{1.0}{1+\exp\left(-\frac{V-5.0}{16.5}\right)}, \text{if in the CTL condition} \\ \frac{1.0}{1+\exp\left(-\frac{V-10.5}{24.7}\right)}, \text{if in the HF condition} \end{cases}$$ (EQ

S3.56)

$$\tau_{x_{s1}} = \frac{1}{\frac{0.0000761(V+44.6)}{1-\exp(-9.97(V+44.6))} + \frac{0.00036(V-0.55)}{\exp(0.128(V-0.55))-1}}$$ (EQ

S3.57)

$$\tau_{x_{s2}} = 2\tau_{x_{s1}}$$ (EQ

S3.58)

3.1.7 L-type Ca^{2+} Current : I_{CaL}

$$I_{CaL} = X_{CaL} d f f_2 f_{Ca} f_{Ca2} \overline{i_{Ca}}$$ (EQ

S3.59)

$$\overline{i_{Ca}} = p_{Ca} z_{Ca}^2 \frac{(V-15)F^2}{RT} \frac{\gamma_{Ca_i}[Ca^{2+}]_r \exp\left(\frac{z_{Ca}F(V-15)}{RT}\right) - \gamma_{Ca_o}[Ca^{2+}]_o}{\exp\left(\frac{z_{Ca}F(V-15)}{RT}\right)-1}$$ (EQ

S3.60)

$$\frac{dd}{dt} = \frac{d_\infty - d}{\tau_d}$$ (EQ

S3.61)

$$d_\infty = \begin{cases} \frac{1.0}{1+\exp\left(-\frac{V-1.7457}{7.2483}\right)}, \text{if in the CTL condition} \\ \frac{1.0}{1+\exp\left(-\frac{V+0.5143}{6.4979}\right)}, \text{if in the HF condition} \end{cases}$$ (EQ

S3.62)

$$\tau_d = \frac{0.59+0.8\exp(0.052(V+13))}{1+\exp(0.132(V+13))}$$ (EQ

S3.63)

$$\frac{df}{dt} = \frac{f_\infty - f}{\tau_f}$$ (EQ

S3.64)

$$f_\infty = \begin{cases} \frac{1.0}{1+\exp\left(\frac{V+24.1014}{7.4776}\right)}, \text{if in the CTL condition} \\ \frac{1.0}{1+\exp\left(\frac{V+26.5377}{6.5704}\right)}, \text{if in the HF condition} \end{cases}$$ (EQ

S3.65)

$$\tau_f = \begin{cases} 0.0033(V+1.0909)^2 + 4.7321, \text{if in the CTL condition} \\ 0.0024(V+24.0)^2 + 4.2625, \text{if in the HF condition} \end{cases}$$ (EQ

S3.66)

$$\frac{df_2}{dt} = \frac{f_{2,\infty} - f_2}{\tau_{f_2}}$$ (EQ

S3.67)

$$f_{2,\infty} = f_{\infty}$$ (EQ

S3.68)

$$\tau_{f_2} = \begin{cases} 0.0813(V - 18.8106)^2 + 36.9839, \text{if in the CTL condition} \\ 0.0856(V - 5.1898)^2 + 56.2764, \text{if in the HF condition} \end{cases}$$ (EQ

S3.69)

$$\frac{df_{Ca}}{dt} = \frac{f_{Ca,\infty} - f_{Ca}}{\tau_{f_{Ca}}}$$ (EQ

S3.70)

$$f_{Ca,\infty} = \frac{0.3}{1 - \frac{I_{CaL}}{0.05}} + \frac{0.55}{1 + \frac{[Ca^{2+}]_r}{0.003}} + 0.15$$ (EQ

S3.71)

$$\tau_{f_{Ca}} = 0.5 + \frac{10.0 Ca_{MK,act}}{Ca_{MK,act} + k_{m,Ca,MK}} + \frac{1.0}{1 + \frac{[Ca^{2+}]_r}{0.003}}$$ (EQ

S3.72)

$$\frac{df_{Ca2}}{dt} = \frac{f_{Ca2,\infty} - f_{Ca2}}{\tau_{f_{Ca2}}}$$ (EQ

S3.73)

$$f_{Ca2,\infty} = \frac{1.0}{1 - \frac{I_{CaL}}{0.01}}$$ (EQ

S3.74)

$$\tau_{f_{Ca2}} = 125.0 + \frac{300.0}{1 + \exp\left(-\frac{I_{CaL} + 0.175}{0.04}\right)}$$ (EQ

S3.75)

3.1.8 T-type Ca^{2+} Current : I_{CaT}

$$I_{CaT} = g_{CaT} bg(V - E_{Ca})$$ (EQ

S3.76)

$$\frac{db}{dt} = \frac{b_{\infty} - b}{\tau_b}$$ (EQ

S3.77)

$$b_{\infty} = \begin{cases} \frac{1.0}{1 + \exp\left(-\frac{V + 32.4575}{8.4014}\right)}, \text{if in the CTL condition} \\ \frac{1.0}{1 + \exp\left(-\frac{V + 31.2424}{9.3111}\right)}, \text{if in the HF condition} \end{cases}$$ (EQ

S3.78)

$$\tau_b = \frac{1.0}{\alpha_b + \beta_b}$$ (EQ

S3.79)

$$\alpha_b = 1.068 \exp\left(\frac{V + 16.3}{30.0}\right)$$ (EQ

S3.80)

$$\beta_b = 1.068\exp\left(-\frac{V+16.3}{30.0}\right) \quad \text{(EQ S3.81)}$$

$$\frac{dg}{dt} = \frac{g_\infty - g}{\tau_g} \quad \text{(EQ S3.82)}$$

$$g_\infty = \begin{cases} \frac{1.0}{1+\exp\left(\frac{V+60.4363}{4.6941}\right)}, \text{if in the CTL condition} \\ \frac{1.0}{1+\exp\left(\frac{V+63.4805}{4.8531}\right)}, \text{if in the HF condition} \end{cases} \quad \text{(EQ S3.83)}$$

$$\tau_g = \frac{1.0}{\alpha_g + \beta_g} \quad \text{(EQ S3.84)}$$

$$\alpha_g = 0.015\exp\left(-\frac{V+71.7}{83.3}\right) \quad \text{(EQ S3.85)}$$

$$\beta_g = 0.015\exp\left(\frac{V+71.7}{15.4}\right) \quad \text{(EQ S3.86)}$$

3.1.9 Plateau Current : I_{Kp}

$$I_{Kp} = g_{Kp} k_p (V - E_K) \quad \text{(EQ S3.87)}$$

$$k_p = \frac{1.0}{1+\exp\left(\frac{7.488-V}{5.98}\right)} \quad \text{(EQ S3.88)}$$

3.1.10 Ca^{2+}-dependent Transient Outward Cl^- Current : I_{to2}

$$I_{to2} = 20\overline{i_{to2}} a_{to1} \quad \text{(EQ S3.89)}$$

$$\overline{i_{to2}} = p_{cl} z_{cl}^2 \frac{VF^2}{RT} \frac{[Cl^-]_i - [Cl^-]_o \exp\left(-\frac{z_{Cl}VF}{RT}\right)}{1-\exp\left(-\frac{z_{Cl}VF}{RT}\right)} \quad \text{(EQ S3.90)}$$

$$\frac{da_{to2}}{dt} = \frac{a_{to2,\infty} - a_{to2}}{\tau_{to2}} \quad \text{(EQ S3.91)}$$

$$a_{to2,\infty} = \frac{1.0}{1+\frac{k_{m,to2}}{[Ca^{2+}]_r}} \quad \text{(EQ S3.92)}$$

$$\tau_{a_{to2}} = 1.0 \quad \text{(EQ S3.93)}$$

3.1.11 Na$^+$-Ca^{2+}Exchange Current : NCX

$$NCX = \frac{X_{NCX} i_{NCX,max} [Na^+]_i^3 [Ca^{2+}]_o \exp\left(\frac{0.35VF}{RT}\right) - 1.5[Na^+]_o^3 [Ca^{2+}]_i \exp\left(-\frac{0.65VF}{RT}\right))}{(1+(\frac{k_{m,Ca,act}}{1.5[Ca^{2+}]_i})^2)(1+k_{sat}\exp\left(-\frac{0.65VF}{RT}\right))(d_{NCX1}+d_{NCX2})} \quad \text{(EQ S3.94)}$$

$$d_{NCX1} = k_{m,Ca_o}[Na^+]_i^3 + 1.5k_{m,Na_o}^3[Ca^{2+}]_i + k_{m,Na_i,1}^3[Ca^{2+}]_o(1 + \frac{1.5[Ca^{2+}]_i}{k_{m,Ca_i}}) \quad \text{(EQ S3.95)}$$

$$d_{NCX2} = k_{m,Ca_i}[Na^+]_o^3(1 + (\frac{[Na^+]_i}{k_{m,Na_i,1}})^3) + [Na^+]_i^3[Ca^{2+}]_o + 1.5[Na^+]_o^3[Ca^{2+}]_i \quad \text{(EQ S3.96)}$$

3.1.12 Background Cl$^-$ Current : I_{Clb}

$$I_{Clb} = g_{Clb}(V - E_{Cl}) \quad \text{(EQ S3.97)}$$

3.1.13 Background Ca^{2+} Current : I_{Cab}

$$I_{Cab} = p_{Ca} z_{Ca}^2 \frac{VF^2}{RT} \frac{\gamma_{Ca_i}[Ca^{2+}]_i \exp\left(z_{Ca}\frac{VF}{RT}\right) - \gamma_{Ca_o}[Ca^{2+}]_o}{\exp\left(z_{Ca}\frac{VF}{RT}\right) - 1} \quad \text{(EQ S3.98)}$$

3.1.14 Background Na$^+$ Current : I_{Nab}

$$I_{Nab} = g_{Nab}(V - E_{Na}) \quad \text{(EQ S3.99)}$$

3.1.15 Background K$^+$ Current : I_{Kb}

$$I_{Kb} = g_{Kb}(V - E_K) \quad \text{(EQ S3.100)}$$

3.1.16 Na$^+$ / K$^+$ Pump Current : I_{NaK}

$$I_{NaK} = \overline{g_{NaK}} f_{NaK} \frac{1.0}{1+(\frac{k_{m,Na_i,2}}{[Na^+]_i})^2} \frac{[K^+]_o}{[K^+]_o + k_{m,K_o}} \quad \text{(EQ S3.101)}$$

$$f_{NaK} = \frac{1.0}{1.0 + 0.1245\exp\left(-\frac{0.1VF}{RT}\right) + 0.0365\sigma\exp\left(-\frac{VF}{RT}\right)} \quad \text{(EQ}$$

S3.102)

$$\sigma = \frac{1}{7}(\exp\left(\frac{[Na^+]_o}{67.3}\right) - 1)$$ (EQ S3.103)

3.1.17 Sarcolemmal Ca^{2+} Pump Current : I_{Cap}

$$I_{Cap} = \overline{i_{Cap}}\frac{[Ca^{2+}]_i}{[Ca^{2+}]_i + k_{m,Cap}}$$ (EQ S3.104)

3.1.18 K^+ / Cl^- Co-transporter

$$CT_{K-Cl} = \overline{CT_{K-Cl}}\frac{E_K - E_{Cl}}{E_K - E_{Cl} + 87.8251}$$ (EQ S3.105)

EQ S3.1.19 Na^+ / Cl^- Co-transporter

$$CT_{Na-Cl} = \overline{CT_{Na-Cl}}\frac{(E_{Na} - E_{Cl})^4}{(E_{Na} - E_{Cl})^4 + 87.8251^4}$$ (EQ S3.106)

3.1.20 Intracellular Ion Concentrations

$$\frac{d[Na^+]_i}{dt} = -\frac{(I_{Na} + I_{NaL} + I_{Nab} + 3I_{NaK} + 3NCX)a_{cap}}{Vol_{myo}F} + CT_{Na-Cl}$$ (EQ S3.107)

$$\frac{d[K^+]_i}{dt} = -\frac{(I_{to1} + I_{K1} + I_{Kr} + I_{Ks} + I_{Kp} + I_{Kb} - 2I_{NaK})a_{cap}}{Vol_{myo}F} + CT_{K-Cl}$$ (EQ S3.108)

$$\frac{d[Cl^-]_i}{dt} = -\frac{(I_{to2} + I_{Clb})a_{cap}}{Vol_{myo}F} + CT_{Na-Cl} + CT_{K-Cl}$$ (EQ S3.109)

3.1.21 Intracellular Ca^{2+} Concentration

$$\frac{d[Ca^{2+}]_i}{dt} = -\frac{(I_{Cab} + I_{Cap} - 2NCX)a_{cap}}{z_{Ca}Vol_{myo}F} + \left(q_{up} - q_{leak}\right)\frac{Vol_{nsr}}{Vol_{myo}} - q_{diff}\frac{Vol_{ss}}{Vol_{myo}}$$ (EQ S3.110)

$$d_{myo} = -k_{m,TRPN}k_{m,CMDN}[Ca^{2+}]_{tot}$$ (EQ S3.111)

$$c_{myo} = k_{m,CMDN}k_{m,TRPN} - [Ca^{2+}]_{tot}\left(k_{m,TRPN} + k_{m,CMDN}\right)$$

$$+\overline{TRPN} \times k_{m,CMDN} + \overline{CMDN} \times k_{m,TRPN}$$ (EQ S3.112)

$$b_{myo} = \overline{CMDN} + \overline{TRPN} - [Ca^{2+}]_{tot} + k_{m,TRPN} + k_{m,CMDN}$$ (EQ S3.113)

$$[Ca^{2+}]_{tot} = TRPN + CMDN + d[Ca^{2+}]_i + [Ca^{2+}]_i$$ (EQ S3.114)

$$CMDN = \overline{CMDN}(\frac{[Ca^{2+}]_i}{[Ca^{2+}]_i + k_{m,CMDN}})$$ (EQ S3.115)

$$TRPN = \overline{TRPN}(\frac{[Ca^{2+}]_i}{[Ca^{2+}]_i + k_{m,TRPN}})$$ (EQ S3.116)

$$[Ca^{2+}]_i = \frac{2}{3}\sqrt{b_{myo}^2 - 3c_{myo}}\cos(\frac{1}{3}\arccos(\frac{9b_{myo}c_{myo} - 2b_{myo}^3 - 27d_{myo}}{2(b_{myo}^2 - 3c_{myo})^{1.5}})) - \frac{b_{myo}}{3}$$

(EQ S3.117)

3.1.22 Ca^{2+} / Calmodulin-dependent Protein Kinase

$$Ca_{MK,act} = Ca_{MK,bound} + Ca_{MK,trap}$$ (EQ S3.118)

$$\frac{dCa_{MK,trap}}{dt} = \alpha_{Ca,MK}Ca_{MK,bound}(Ca_{MK,bound} + Ca_{MK,trap}) - \beta_{Ca,MK}Ca_{MK,trap}$$

(EQ S3.119)

$$Ca_{MK,bound} = \frac{Ca_{MK,0}(1 - Ca_{MK,trap})}{1 + \frac{k_{m,Ca,MK}}{[Ca^{2+}]_r}}$$ (EQ S3.120)

3.1.23 NSR Ca^{2+} Concentration

$$\frac{d[Ca^{2+}]_{NSR}}{dt} = q_{up} - q_{leak} - q_{tr}\frac{Vol_{JSR}}{Vol_{NSR}}$$ (EQ S3.121)

3.1.24 JSR Ca^{2+} Concentration

$$\frac{d[Ca^{2+}]_{JSR}}{dt} = \frac{q_{tr} - q_{rel}}{1 + \overline{CSQN}\frac{k_{m,CSQN}}{(k_{m,CSQN} + [Ca^{2+}]_{JSR})^2}}$$ (EQ S3.122)

3.1.25 Restricted Space Ca^{2+} Concentration

$$[Ca^{2+}]_r = \frac{2}{3}\sqrt{b_1^2 - 3c_1}\cos(\frac{1}{3}\arccos(\frac{9b_1c_1 - 2b_1^3 - 27d_1}{2(b_1^2 - 3c_1)^{1.5}})) - \frac{b_1}{3}$$ (EQ

S3.123)

$$d_1 = -k_{m,b,SR}k_{m,b,SL}[Ca^{2+}]_{r,tot}$$ (EQ S3.124)

$$c_1 = k_{m,b,SR}k_{m,b,SL} - [Ca^{2+}]_{r,tot}\left(k_{m,b,SR} + k_{m,b,SL}\right) + \overline{b_{SR}}k_{m,b,SL} + \overline{b_{SL}}k_{m,b,SR}$$

(EQ S3.125)

$$b_1 = \overline{b_{SR}} + \overline{b_{SL}} - [Ca^{2+}]_{r,tot} + k_{m,b,SR} + k_{m,b,SL}$$ (EQ S3.126)

$$[Ca^{2+}]_{r,tot} = [Ca^{2+}]_r + b_{SR} + b_{SL} + d[Ca^{2+}]_r$$ (EQ S3.127)

$$b_{SL} = \overline{b_{SL}}\left(\frac{[Ca^{2+}]_r}{[Ca^{2+}]_r + k_{m,b,SL}}\right)$$ (EQ S3.128)

$$b_{SR} = \overline{b_{SR}}\left(\frac{[Ca^{2+}]_r}{[Ca^{2+}]_r + k_{m,b,SR}}\right)$$ (EQ S3.129)

$$\frac{d[Ca^{2+}]_r}{dt} = -\frac{I_{CaL}a_{Cap}}{Vol_{ss}z_{Ca}F} + q_{rel}\frac{Vol_{JSR}}{Vol_{ss}} - \frac{[Ca^{2+}]_r - [Ca^{2+}]_i}{\tau_{ss}}$$ (EQ S3.130)

3.1.26 SR Release Flux

$$q_{rel} = \overline{g_{rel}}r_o r_i([Ca^{2+}]_{JSR} - [Ca^{2+}]_r)$$ (EQ S3.131)

$$\overline{g_{rel}} = \begin{cases} 3000v_g, \text{if in the CTL condition} \\ 1800v_g, \text{if in the HF condition} \end{cases}$$ (EQ S3.132)

$$v_g = \frac{1.0}{1 + \exp\left(\frac{x_{CaL}\overline{i_{Ca}} + 13}{5}\right)}$$ (EQ S3.133)

$$\frac{dr_i}{dt} = \frac{r_{i,\infty} - r_i}{\tau_{r_i}}$$ (EQ S3.134)

$$r_{i,\infty} = \frac{1.0}{1 + \exp\left(\frac{[Ca^{2+}]_r - 0.0004 + 0.002Ca_{fac}}{0.000025}\right)}$$ (EQ S3.135)

$$\tau_{r_i} = \frac{350 - \tau_{Ca,MK}}{1 + \exp\left(\frac{[Ca^{2+}]_r - 0.003 + 0.003Ca_{fac}}{0.0002}\right)} + 3.0 + \tau_{Ca,MK}$$ (EQ S3.136)

$$Ca_{fac} = \frac{1.0}{1 + \exp\left(\frac{I_{CaL} + 0.05}{0.015}\right)}$$ (EQ

S3.137)

$$\tau_{Ca,MK} = \overline{\tau_{Ca,MK}} \frac{Ca_{MK,act}}{k_{m,Ca,MK} + Ca_{MK,act}}$$ (EQ

S3.138)

$$\frac{dr_o}{dt} = \frac{r_{o,\infty} - r_o}{\tau_{r_o}}$$ (EQ

S3.139)

$$r_{o,\infty} = r_{o,\infty,JSR} \frac{I_{CaL}^2}{I_{CaL}^2 + 1}$$ (EQ

S3.140)

$$r_{o,\infty,JSR} = \frac{[Ca^{2+}]_{JSR}^{1.9}}{[Ca^{2+}]_{JSR}^{1.9} + (\frac{49.28[Ca^{2+}]_r}{[Ca^{2+}]_r + 0.0028})^{1.9}}$$ (EQ

S3.141)

$$\tau_{r_o} = 3.0$$ (EQ

S3.142)

3.1.27 SR Leak Flux

$$q_{leak} = \overline{q_{leak}} \frac{[Ca^{2+}]_{NSR}}{\overline{NSR}}$$ (EQ

S3.143)

3.1.28 SR Uptake Flux

$$q_{up} = X_{qup}(dq_{up,Ca,MK} + 1)\overline{q_{up}} \frac{[Ca^{2+}]_i}{[Ca^{2+}]_i + k_{m,up} - dk_{m,plb}}$$ (EQ

S3.144)

$$dq_{up,Ca,MK} = d\overline{q_{up,Ca,MK}} \frac{Ca_{MK,act}}{k_{m,Ca,MK} + Ca_{MK,act}}$$ (EQ

S3.145)

$$dk_{m,pld} = dk_{m,pld} \frac{Ca_{MK,act}}{k_{m,Ca,MK} + Ca_{MK,act}}$$ (EQ

S3.146)

3.1.29 SR Transfer Flux

$$q_{tr} = \frac{[Ca^{2+}]_{NSR} - [Ca^{2+}]_{JSR}}{\tau_{tr}}$$ (EQ

S3.147)

3.1.30 Equilibrium Potentials

$$E_{Na} = \frac{RT}{F} \ln \frac{[Na^+]_o}{[Na^+]_i}$$ (EQ

S3.148)

$$E_K = \frac{RT}{F} \ln \frac{[K^+]_o}{[K^+]_i} \quad \text{(EQ S3.149)}$$

$$E_{Ca} = \frac{RT}{2F} \ln \frac{[Ca^{2+}]_o}{[Ca^{2+}]_i} \quad \text{(EQ S3.150)}$$

$$E_{Cl} = \frac{RT}{F} \ln \frac{[Cl^-]_o}{[Cl^-]_i} \quad \text{(EQ S3.151)}$$

$$E_{K,s} = \frac{RT}{F} \ln \frac{[K^+]_o + r_{NaK}[Na^+]_o}{[K^+]_i + r_{NaK}[Na^+]_i} \quad \text{(EQ S3.152)}$$

S3.2 Model Parameters

Supplementary Table S17 Model parameters of canine PF model

Parameters	Values	
	CTL	HF
Universal gas constant, R	8314 J/(K*kmol)	8314 J/(K*kmol)
Faraday's constant, F	96485 C/mol	96485 C/mol
Temperature, T	310 K	310 K
External Na^+ concentration, $[Na^+]_o$	140.0 mM	140.0 mM
External Ca^{2+} concentration, $[Ca^{2+}]_o$	1.8 mM	1.8 mM
External K^+ concentration, $[K^+]_o$	5.4 mM	5.4 mM
External Cl^- concentration, $[Cl^-]_o$	100.0 mM	100.0 mM
Na^+ ion valence, z_{Na}	1	1
Ca^{2+} ion valence, z_{Ca}	2	2
K^+ ion valence, z_K	1	1
Cl^- ion valence, z_{Cl}	-1	-1
Internal Na^+ activity coefficient, γ_{Na_i}	0.75	0.75
External Na^+ activity coefficient, γ_{Na_o}	0.75	0.75
Internal Ca^{2+} activity coefficient, γ_{Ca_i}	1.0	1.0
External Ca^{2+} activity coefficient, γ_{Ca_o}	0.341	0.341
Internal K^+ activity coefficient, γ_{K_i}	0.75	0.75
External K^+ activity coefficient, γ_{K_o}	0.75	0.75
Maximum conductance of I_{Na}, g_{Na}	8.25 pA/pF	4.95 pA/pF
Maximum conductance of I_{NaL}, g_{NaL}	0.05 pA/pF	0.05 pA/pF

Additional scaling factor for I_{NaL}, X_{NaL}	1.15 pA/pF	1.15 pA/pF
Maximum conductance of I_{to1}, g_{to1}	0.1995 pA/pF	0.1425 pA/pF
Additional scaling factor for I_{to1}, X_{to1}	0.743997052	0.743997052
Additional scaling factor for I_{Ks}, X_{Ks}	2.11191198	2.11191198
Additional scaling factor for I_{CaL}, X_{CaL}	0.38112805	0.21790932
Maximum conductance of I_{CaT}, g_{CaT}	0.14 pA/pF	0.1254 pA/pF
Half-saturation coefficient of CaM, $k_{m,Ca,M}$	0.0015 mM	0.0015 mM
Membrane permeability to Ca^{2+}, p_{Ca}	0.000243 cm/s	0.000243 cm/s
Maximum conductance of I_{Kp}, g_{Kp}	0.00276 pA/pF	0.00276 pA/pF
Maximum conductance of I_{Nab}, g_{Nab}	0.0025 pA/pF	0.0025 pA/pF
Maximum conductance of I_{Cab}, g_{Cab}	0.005 pA/pF	0.005 pA/pF
Constant for low affinity binding of subspace Ca^{2+}, $k_{m,to2}$	0.1502 mM	0.1502 mM
Membrane permeability to Cl^{-}, p_{cl}	0.0000004 cm/s	0.0000004 cm/s
Additional scaling factor for the NCX, X_{NCX}	0.4	0.4
Maximal NCX, $i_{NCX,max}$	4.5 pA/pF	4.5 pA/pF
Half-saturation concentration for $[Ca^{2+}]_i$ activation, $k_{m,Ca,act}$	0.000125 mM	0.000125 mM
Half-saturation concentration for $[Na^{+}]_i$, $k_{m,Na_i,1}$	12.3 mM	12.3 mM
Half-saturation concentration for $[Na^{+}]_o$, k_{m,Na_o}	87.5 mM	87.5 mM

Half-saturation concentration for $[Ca^{2+}]_i$, k_{m,Ca_i}	0.0036 mM	0.0036 mM
Half-saturation concentration for $[Ca^{2+}]_o$, k_{m,Ca_o}	1.3 mM	1.3 mM
saturation factor for NCX at negative potentials, k_{sat}	0.27	0.27
Maximum conductance of I_{Clb}, g_{Clb}	0.000225 pA/pF	0.000225 pA/pF
Maximum I_{NaK}, $\overline{g_{NaK}}$	0.61875 pA/pF	0.61875 pA/pF
Half-saturation concentration for $[Na^+]_i$, $k_{m,Na_i,2}$	10.0 mM	10.0 mM
Half-saturation concentration for $[K^+]_o$, k_{m,K_o}	1.5 mM	1.5 mM
Maximum I_{Cap}, $\overline{i_{Cap}}$	0.0575 pA/pF	0.0575 pA/pF
half saturation concentration for I_{Cap}, $k_{m,Cap}$	0.0005 mM	0.0005 mM
Maximum K^+ / Cl^- transport, $\overline{CT_{K-Cl}}$	7.0756×10^{-6} mM/ms	7.0756×10^{-6} mM/ms
Maximum Na^+ / Cl^- transport, $\overline{CT_{Na-Cl}}$	9.8443×10^{-6} mM/ms	9.8443×10^{-6} mM/ms
Factor for modulation of time constant for inactivation of q_{rel} by CaMKII, maximum, $\overline{\tau_{Ca,MK}}$	10.0	10.0
Maximum leak from NSR to myoplasm, $\overline{q_{leak}}$	0.004375 mM/ms	0.002861 mM/ms
Maximum modulation of phospholamban (half-saturation) by CAMK, $\overline{dk_{m,pld}}$	0.00017	0.00017
Half-saturation concentration of q_{up}, $k_{m,up}$	0.00092 mmol	0.00092 mmol

Maximum uptake from myoplasm to the NSR, $\overline{q_{up}}$	0.0035 mM/ms	0.0035 mM/ms
Time constant of transfer from the NSR to the JSR, τ_{tr}	120.0 ms	120.0 ms
Maximum Ca^{2+} binding by anionic binding sites in the subspace, $\overline{b_{SR}}$	0.047 mM	0.047 mM
Half-saturation coefficient of anionic binding sites in the subspace, $k_{m,b,SR}$	0.00087 mM	0.00087 mM
Maximum Ca^{2+} binding by sarcolemmal binding sites in the subspace, $\overline{b_{SL}}$	1.124 mM	1.124 mM
Half-saturation coefficient of sarcolemmal binding sites in the subspace, $k_{m,b,SL}$	0.0087 mM	0.0087 mM
Time constant for diffusion between restricted space and myoplasm, τ_{ss}	0.2 ms	0.2 ms
Maximum Ca^{2+} buffered by calsequestrin, $\overline{CSQN}$	10.0 mM	10.0 mM
Equilibrium constant for calsequestrin buffering, $k_{m,CSQN}$	0.8 mM	0.8 mM
Maximum Ca^{2+} buffered by calmodulin, $\overline{CMDN}$	0.05 mM	0.05 mM
Equilibrium constant for calmodulin buffering, $k_{m,CMDN}$	0.00238 mM	0.00238 mM
Maximum Ca^{2+} buffered by troponin, $\overline{TRPN}$	0.07 mM	0.07 mM
Equilibrium constant for troponin buffering, $k_{m,TRPN}$	0.0005 mM	0.0005 mM
Fraction of active CaMKII binding sites at equilibrium, $Ca_{MK,0}$	0.05	0.05

Phosphorylation rate of CaMKII, $\alpha_{Ca,MK}$	0.05 1/ms	0.05 1/ms
Dephosphorylation rate of CaMKII, $\beta_{Ca,MK}$	0.00068 1/ms	0.00068 1/ms
Half-saturation coefficient of CaMKII, $k_{m,Ca,MK}$	0.15	0.15

S3.3 Initial Conditions

Supplementary Table S18 Initial conditions of canine PF model

Parameters	Values	
	CTL	HF
V (mV)	-85.48049561	-83.73141161
m	0.00735156	0.00972549
h	0.98643410	0.97998154
j	0.99139961	0.98746650
m_L	0.00142650	0.00190829
h_L	0.92239032	0.90144893
d	0.00000594	0.00000274
f	0.99972752	0.99983410
f_2	0.64277048	0.71462375
f_{Ca}	0.96642277	0.94082284
f_{Ca2}	0.89392961	0.93070368
b	0.00181255	0.00355002

g	0.99519543	0.98476894
a	0.00042281	0.00029119
i	0.98089094	0.97438920
i_2	0.98088129	0.97436448
x_r	0.114882605	0.14543401
x_{s1}	0.00426527	0.00474219
x_{s2}	0.00427244	0.00475369
a_{to2}	0.00122949	0.00237885
I_{CaL} (pA/pF)	0.0	0.0
d_2	8.98067053	8.96769822
r_i	0.78753547	0.10155588
r_o	0.0	0.0
$[Ca^{2+}]_r$ (mM)	0.00018483	0.00035805
$[Ca^{2+}]_{JSR}$ (mM)	0.83891651	0.52837062
$[Ca^{2+}]_{NSR}$ (mM)	0.95684124	0.52692759
$[Na^+]_i$ (mM)	10.83208983	11.00887330
$[K^+]_i$ (mM)	141.26205543	141.13116016
$[Cl^-]_i$ (mM)	18.97852032	18.88911436
$[Ca^{2+}]_i$ (mM)	0.00017096	0.00033685
$Ca_{MK,act}$	0.03710953	0.04896265
$Ca_{MK,trap}$	0.03179889	0.03971009

Supplementary S4 Appendices References

AKAR, F. G., WU, R. C., JUANG, G. J., TIAN, Y., BURYSEK, M., DISILVESTRE, D., XIONG, W., ARMOUNDAS, A. A. & TOMASELLI, G. F. 2005. Molecular mechanisms underlying K+ current downregulation in canine tachycardia-induced heart failure. *Am J Physiol Heart Circ Physiol,* 288**,** H2887-96.

ALLEN, P. D., SCHMIDT, T. A., MARSH, J. D. & KJELDSEN, K. 1992. Na,K-ATPase expression in normal and failing human left ventricle. *Basic Res Cardiol,* 87 Suppl 1**,** 87-94.

ANTZELEVITCH, C., SHIMIZU, W., YAN, G. X., SICOURI, S., WEISSENBURGER, J., NESTERENKO, V. V., BURASHNIKOV, A., DI DIEGO, J., SAFFITZ, J. & THOMAS, G. P. 1999. The M cell: its contribution to the ECG and to normal and abnormal electrical function of the heart. *J Cardiovasc Electrophysiol,* 10**,** 1124-52.

ANYUKHOVSKY, E. P., SOSUNOV, E. A. & ROSEN, M. R. 1996. Regional differences in electrophysiological properties of epicardium, midmyocardium, and endocardium. In vitro and in vivo correlations. *Circulation,* 94**,** 1981-8.

ASLANIDI, O. V., STEWART, P., BOYETT, M. R. & ZHANG, H. 2009. Optimal velocity and safety of discontinuous conduction through the heterogeneous Purkinje-ventricular junction. *Biophys J,* 97**,** 20-39.

BAARTSCHEER, A., SCHUMACHER, C. A., BELTERMAN, C. N., CORONEL, R. & FIOLET, J. W. 2003. [Na+]i and the driving force of the Na+/Ca2+-exchanger in heart failure. *Cardiovasc Res,* 57**,** 986-95.

BALATI, B., IOST, N., SIMON, J., VARRO, A. & PAPP, J. G. 2000. Analysis of the electrophysiological effects of ambasilide, a new antiarrhythmic agent, in canine isolated ventricular muscle and purkinje fibers. *Gen Pharmacol,* 34**,** 85-93.

BALATI, B., VARRO, A. & PAPP, J. G. 1998. Comparison of the cellular electrophysiological characteristics of canine left ventricular epicardium, M cells, endocardium and Purkinje fibres. *Acta Physiol Scand,* 164**,** 181-90.

BENSON, A. P., ASLANIDI, O. V., ZHANG, H. & HOLDEN, A. V. 2008. The canine virtual ventricular wall: a platform for dissecting pharmacological effects on propagation and arrhythmogenesis. *Prog Biophys Mol Biol,* 96**,** 187-208.

BEUCKELMANN, D. J. & ERDMANN, E. 1992. Ca(2+)-currents and intracellular [Ca2+]i-transients in single ventricular myocytes isolated from terminally failing human myocardium. *Basic Res Cardiol,* 87 Suppl 1**,** 235-43.

BEUCKELMANN, D. J., NABAUER, M. & ERDMANN, E. 1992. Intracellular calcium handling in isolated ventricular myocytes from patients with terminal heart failure. *Circulation,* 85**,** 1046-55.

BEUCKELMANN, D. J., NABAUER, M. & ERDMANN, E. 1993. Alterations of K+ currents in isolated human ventricular myocytes from patients with terminal heart failure. *Circ Res,* 73**,** 379-85.

BORLAK, J. & THUM, T. 2003. Hallmarks of ion channel gene expression in end-stage heart failure. *FASEB J,* 17**,** 1592-608.

BUNDGAARD, H. & KJELDSEN, K. 1996. Human myocardial Na,K-ATPase concentration in heart failure. *Mol Cell Biochem,* 163-164**,** 277-83.

CHEN, X., PIACENTINO, V., 3RD, FURUKAWA, S., GOLDMAN, B., MARGULIES, K. B. & HOUSER, S. R. 2002. L-type Ca2+ channel density and regulation are altered in failing human ventricular myocytes and recover after support with mechanical assist devices. *Circ Res,* 91**,** 517-24.

DUMAINE, R. & CORDEIRO, J. M. 2007. Comparison of K+ currents in cardiac Purkinje cells isolated from rabbit and dog. *J Mol Cell Cardiol,* 42**,** 378-89.

FAN, T. H., FRANTZ, R. P., ELAM, H., SAKAMOTO, S., IMAI, N. & LIANG, C. S. 1993. Reductions of myocardial Na-K-ATPase activity and ouabain binding sites in heart failure: prevention by nadolol. *Am J Physiol,* 265**,** H2086-93.

FLESCH, M., SCHWINGER, R. H., SCHIFFER, F., FRANK, K., SUDKAMP, M., KUHN-REGNIER, F., ARNOLD, G. & BOHM, M. 1996. Evidence for functional relevance of an enhanced expression of the Na(+)-Ca2+ exchanger in failing human myocardium. *Circulation,* 94**,** 992-1002.

GUPTA, R. C., SHIMOYAMA, H., TANIMURA, M., NAIR, R., LESCH, M. & SABBAH, H. N. 1997. SR Ca(2+)-ATPase activity and expression in ventricular myocardium of dogs with heart failure. *Am J Physiol,* 273**,** H12-8.

GWATHMEY, J. K., SLAWSKY, M. T., HAJJAR, R. J., BRIGGS, G. M. & MORGAN, J. P. 1990. Role of intracellular calcium handling in force-interval relationships of human ventricular myocardium. *J Clin Invest,* 85**,** 1599-613.

HAN, W., CHARTIER, D., LI, D. & NATTEL, S. 2001. Ionic remodeling of cardiac Purkinje cells by congestive heart failure. *Circulation,* 104**,** 2095-100.

HASENFUSS, G., REINECKE, H., STUDER, R., MEYER, M., PIESKE, B., HOLTZ, J., HOLUBARSCH, C., POSIVAL, H., JUST, H. & DREXLER, H. 1994. Relation between myocardial function and expression of sarcoplasmic reticulum Ca(2+)-ATPase in failing and nonfailing human myocardium. *Circ Res,* 75**,** 434-42.

HE, J., CONKLIN, M. W., FOELL, J. D., WOLFF, M. R., HAWORTH, R. A., CORONADO, R. & KAMP, T. J. 2001. Reduction in density of transverse tubules and L-type Ca(2+) channels in canine tachycardia-induced heart failure. *Cardiovasc Res,* 49**,** 298-307.

HOEKER, G. S., KATRA, R. P., WILSON, L. D., PLUMMER, B. N. & LAURITA, K. R. 2009. Spontaneous calcium release in tissue from the failing canine heart. *Am J Physiol Heart Circ Physiol,* 297**,** H1235-42.

IYER, V., HELLER, V. & ARMOUNDAS, A. A. 2012. Altered spatial calcium regulation enhances electrical heterogeneity in the failing canine left ventricle: implications for electrical instability. *J Appl Physiol,* 112**,** 944-55.

KAAB, S., DIXON, J., DUC, J., ASHEN, D., NABAUER, M., BEUCKELMANN, D. J., STEINBECK, G., MCKINNON, D. & TOMASELLI, G. F. 1998. Molecular basis of transient outward potassium current downregulation in human heart failure: a decrease in Kv4.3 mRNA correlates with a reduction in current density. *Circulation,* 98**,** 1383-93.

KAAB, S., NUSS, H. B., CHIAMVIMONVAT, N., O'ROURKE, B., PAK, P. H., KASS, D. A., MARBAN, E. & TOMASELLI, G. F. 1996. Ionic mechanism of action potential prolongation in ventricular myocytes from dogs with pacing-induced heart failure. *Circ Res,* 78**,** 262-73.

KHARCHE, S., GARRATT, C. J., BOYETT, M. R., INADA, S., HOLDEN, A. V., HANCOX, J. C. & ZHANG, H. 2008. Atrial proarrhythmia due to increased inward rectifier current (I(K1)) arising from KCNJ2 mutation--a simulation study. *Prog Biophys Mol Biol,* 98**,** 186-97.

KIM, C. H., FAN, T. H., KELLY, P. F., HIMURA, Y., DELEHANTY, J. M., HANG, C. L. & LIANG, C. S. 1994. Isoform-specific regulation of myocardial Na,K-ATPase alpha-subunit in congestive heart failure. Role of norepinephrine. *Circulation,* 89**,** 313-20.

KONDO, M., TSUTSUMI, T. & MASHIMA, S. 1999. Potassium channel openers antagonize the effects of class III antiarrhythmic

agents in canine Purkinje fiber action potentials. Implications for prevention of proarrhythmia induced by class III agents. *Jpn Heart J,* 40, 609-19.

LI, G. R., LAU, C. P., DUCHARME, A., TARDIF, J. C. & NATTEL, S. 2002. Transmural action potential and ionic current remodeling in ventricles of failing canine hearts. *Am J Physiol Heart Circ Physiol,* 283, H1031-41.

LI, G. R., LAU, C. P., LEUNG, T. K. & NATTEL, S. 2004. Ionic current abnormalities associated with prolonged action potentials in cardiomyocytes from diseased human right ventricles. *Heart Rhythm,* 1, 460-8.

LI, H. G., JONES, D. L., YEE, R. & KLEIN, G. J. 1993. Arrhythmogenic effects of catecholamines are decreased in heart failure induced by rapid pacing in dogs. *Am J Physiol,* 265, H1654-62.

LINDNER, M., ERDMANN, E. & BEUCKELMANN, D. J. 1998. Calcium content of the sarcoplasmic reticulum in isolated ventricular myocytes from patients with terminal heart failure. *J Mol Cell Cardiol,* 30, 743-9.

LIU, D. W. & ANTZELEVITCH, C. 1995. Characteristics of the delayed rectifier current (IKr and IKs) in canine ventricular epicardial, midmyocardial, and endocardial myocytes. A weaker IKs contributes to the longer action potential of the M cell. *Circ Res,* 76, 351-65.

LIU, D. W., GINTANT, G. A. & ANTZELEVITCH, C. 1993. Ionic bases for electrophysiological distinctions among epicardial, midmyocardial, and endocardial myocytes from the free wall of the canine left ventricle. *Circ Res,* 72, 671-87.

MAGUY, A., LE BOUTER, S., COMTOIS, P., CHARTIER, D., VILLENEUVE, L., WAKILI, R., NISHIDA, K. & NATTEL, S. 2009. Ion channel subunit expression changes in cardiac Purkinje fibers: a potential role in conduction abnormalities associated with congestive heart failure. *Circ Res,* 104, 1113-22.

MALTSEV, V. A., REZNIKOV, V., UNDROVINAS, N. A., SABBAH, H. N. & UNDROVINAS, A. 2008. Modulation of late sodium current by Ca2+, calmodulin, and CaMKII in normal and failing dog cardiomyocytes: similarities and differences. *Am J Physiol Heart Circ Physiol,* 294, H1597-608.

MALTSEV, V. A., SABBAB, H. N. & UNDROVINAS, A. I. 2002. Down-regulation of sodium current in chronic heart failure: effect of long-term therapy with carvedilol. *Cell Mol Life Sci,* 59, 1561-8.

MALTSEV, V. A., SABBAH, H. N., TANIMURA, M., LESCH, M., GOLDSTEIN, S. & UNDROVINAS, A. I. 1998. Relationship

between action potential, contraction-relaxation pattern, and intracellular Ca2+ transient in cardiomyocytes of dogs with chronic heart failure. *Cell Mol Life Sci,* 54**,** 597-605.

MALTSEV, V. A., SILVERMAN, N., SABBAH, H. N. & UNDROVINAS, A. I. 2007. Chronic heart failure slows late sodium current in human and canine ventricular myocytes: implications for repolarization variability. *Eur J Heart Fail,* 9**,** 219-27.

MERCADIER, J. J., LOMPRE, A. M., DUC, P., BOHELER, K. R., FRAYSSE, J. B., WISNEWSKY, C., ALLEN, P. D., KOMAJDA, M. & SCHWARTZ, K. 1990. Altered sarcoplasmic reticulum Ca2(+)-ATPase gene expression in the human ventricle during end-stage heart failure. *J Clin Invest,* 85**,** 305-9.

MEYER, M., SCHILLINGER, W., PIESKE, B., HOLUBARSCH, C., HEILMANN, C., POSIVAL, H., KUWAJIMA, G., MIKOSHIBA, K., JUST, H., HASENFUSS, G. & ET AL. 1995. Alterations of sarcoplasmic reticulum proteins in failing human dilated cardiomyopathy. *Circulation,* 92**,** 778-84.

NORGAARD, A., BAGGER, J. P., BJERREGAARD, P., BAANDRUP, U., KJELDSEN, K. & THOMSEN, P. E. 1988. Relation of left ventricular function and Na,K-pump concentration in suspected idiopathic dilated cardiomyopathy. *Am J Cardiol,* 61**,** 1312-5.

O'ROURKE, B., KASS, D. A., TOMASELLI, G. F., KAAB, S., TUNIN, R. & MARBAN, E. 1999. Mechanisms of altered excitation-contraction coupling in canine tachycardia-induced heart failure, I: experimental studies. *Circ Res,* 84**,** 562-70.

PETKOVA-KIROVA, P. S., GURSOY, E., MEHDI, H., MCTIERNAN, C. F., LONDON, B. & SALAMA, G. 2006. Electrical remodeling of cardiac myocytes from mice with heart failure due to the overexpression of tumor necrosis factor-alpha. *Am J Physiol Heart Circ Physiol,* 290**,** H2098-107.

PIACENTINO, V., 3RD, WEBER, C. R., CHEN, X., WEISSER-THOMAS, J., MARGULIES, K. B., BERS, D. M. & HOUSER, S. R. 2003. Cellular basis of abnormal calcium transients of failing human ventricular myocytes. *Circ Res,* 92**,** 651-8.

POGWIZD, S. M., QI, M., YUAN, W., SAMAREL, A. M. & BERS, D. M. 1999. Upregulation of Na(+)/Ca(2+) exchanger expression and function in an arrhythmogenic rabbit model of heart failure. *Circ Res,* 85**,** 1009-19.

POGWIZD, S. M., SCHLOTTHAUER, K., LI, L., YUAN, W. & BERS, D. M. 2001. Arrhythmogenesis and contractile dysfunction in heart failure: Roles of sodium-calcium exchange, inward rectifier

potassium current, and residual beta-adrenergic responsiveness. *Circ Res,* 88**,** 1159-67.

REINECKE, H., STUDER, R., VETTER, R., JUST, H., HOLTZ, J. & DREXLER, H. 1996. Role of the cardiac sarcolemmal Na(+)-Ca2+ exchanger in end-stage human heart failure. *Ann N Y Acad Sci,* 779**,** 543-5.

ROBINSON, R. B., BOYDEN, P. A., HOFFMAN, B. F. & HEWETT, K. W. 1987. Electrical restitution process in dispersed canine cardiac Purkinje and ventricular cells. *Am J Physiol,* 253**,** H1018-25.

ROSE, J., ARMOUNDAS, A. A., TIAN, Y., DISILVESTRE, D., BURYSEK, M., HALPERIN, V., O'ROURKE, B., KASS, D. A., MARBAN, E. & TOMASELLI, G. F. 2005. Molecular correlates of altered expression of potassium currents in failing rabbit myocardium. *Am J Physiol Heart Circ Physiol,* 288**,** H2077-87.

ROZANSKI, G. J., XU, Z., WHITNEY, R. T., MURAKAMI, H. & ZUCKER, I. H. 1997. Electrophysiology of rabbit ventricular myocytes following sustained rapid ventricular pacing. *J Mol Cell Cardiol,* 29**,** 721-32.

SCHWINGER, R. H., WANG, J., FRANK, K., MULLER-EHMSEN, J., BRIXIUS, K., MCDONOUGH, A. A. & ERDMANN, E. 1999. Reduced sodium pump alpha1, alpha3, and beta1-isoform protein levels and Na+,K+-ATPase activity but unchanged Na+-Ca2+ exchanger protein levels in human heart failure. *Circulation,* 99**,** 2105-12.

SHAMRAJ, O. I., GRUPP, I. L., GRUPP, G., MELVIN, D., GRADOUX, N., KREMERS, W., LINGREL, J. B. & DE POVER, A. 1993. Characterisation of Na/K-ATPase, its isoforms, and the inotropic response to ouabain in isolated failing human hearts. *Cardiovasc Res,* 27**,** 2229-37.

SHIMIZU, W. & ANTZELEVITCH, C. 1997. Sodium channel block with mexiletine is effective in reducing dispersion of repolarization and preventing torsade des pointes in LQT2 and LQT3 models of the long-QT syndrome. *Circulation,* 96**,** 2038-47.

SHIMIZU, W. & ANTZELEVITCH, C. 1998. Cellular basis for the ECG features of the LQT1 form of the long-QT syndrome: effects of beta-adrenergic agonists and antagonists and sodium channel blockers on transmural dispersion of repolarization and torsade de pointes. *Circulation,* 98**,** 2314-22.

SHIMIZU, W. & ANTZELEVITCH, C. 1999. Cellular and ionic basis for T-wave alternans under long-QT conditions. *Circulation,* 99**,** 1499-507.

SICOURI, S. & ANTZELEVITCH, C. 1991. A subpopulation of cells with unique electrophysiological properties in the deep subepicardium of the canine ventricle. The M cell. *Circ Res,* 68**,** 1729-41.

SICOURI, S. & ANTZELEVITCH, C. 1995. Electrophysiologic characteristics of M cells in the canine left ventricular free wall. *J Cardiovasc Electrophysiol,* 6**,** 591-603.

SICOURI, S., FISH, J. & ANTZELEVITCH, C. 1994. Distribution of M cells in the canine ventricle. *J Cardiovasc Electrophysiol,* 5**,** 824-37.

STUDER, R., REINECKE, H., BILGER, J., ESCHENHAGEN, T., BOHM, M., HASENFUSS, G., JUST, H., HOLTZ, J. & DREXLER, H. 1994. Gene expression of the cardiac Na(+)-Ca2+ exchanger in end-stage human heart failure. *Circ Res,* 75**,** 443-53.

TAKAHASHI, T., ALLEN, P. D., LACRO, R. V., MARKS, A. R., DENNIS, A. R., SCHOEN, F. J., GROSSMAN, W., MARSH, J. D. & IZUMO, S. 1992. Expression of dihydropyridine receptor (Ca2+ channel) and calsequestrin genes in the myocardium of patients with end-stage heart failure. *J Clin Invest,* 90**,** 927-35.

THURINGER, D., DEROUBAIX, E., COULOMBE, A., CORABOEUF, E. & MERCADIER, J. J. 1996. Ionic basis of the action potential prolongation in ventricular myocytes from Syrian hamsters with dilated cardiomyopathy. *Cardiovasc Res,* 31**,** 747-57.

TSUJI, Y., ZICHA, S., QI, X. Y., KODAMA, I. & NATTEL, S. 2006. Potassium channel subunit remodeling in rabbits exposed to long-term bradycardia or tachycardia: discrete arrhythmogenic consequences related to differential delayed-rectifier changes. *Circulation,* 113**,** 345-55.

UNDROVINAS, A. I., MALTSEV, V. A. & SABBAH, H. N. 1999. Repolarization abnormalities in cardiomyocytes of dogs with chronic heart failure: role of sustained inward current. *Cell Mol Life Sci,* 55**,** 494-505.

VALDIVIA, C. R., CHU, W. W., PU, J., FOELL, J. D., HAWORTH, R. A., WOLFF, M. R., KAMP, T. J. & MAKIELSKI, J. C. 2005. Increased late sodium current in myocytes from a canine heart failure model and from failing human heart. *J Mol Cell Cardiol,* 38**,** 475-83.

WANG, Z., YUE, L., WHITE, M., PELLETIER, G. & NATTEL, S. 1998. Differential distribution of inward rectifier potassium channel transcripts in human atrium versus ventricle. *Circulation,* 98**,** 2422-8.

WHITMER, J. T., KUMAR, P. & SOLARO, R. J. 1988. Calcium transport properties of cardiac sarcoplasmic reticulum from cardiomyopathic Syrian hamsters (BIO 53.58 and 14.6): evidence for a quantitative defect in dilated myopathic hearts not evident in hypertrophic hearts. *Circ Res,* 62**,** 81-5.

YAN, G. X. & ANTZELEVITCH, C. 1998. Cellular basis for the normal T wave and the electrocardiographic manifestations of the long-QT syndrome. *Circulation,* 98**,** 1928-36.

YAN, G. X., SHIMIZU, W. & ANTZELEVITCH, C. 1998. Characteristics and distribution of M cells in arterially perfused canine left ventricular wedge preparations. *Circulation,* 98**,** 1921-7.

YAO, A., SU, Z., NONAKA, A., ZUBAIR, I., SPITZER, K. W., BRIDGE, J. H., MUELHEIMS, G., ROSS, J., JR. & BARRY, W. H. 1998. Abnormal myocyte Ca2+ homeostasis in rabbits with pacing-induced heart failure. *Am J Physiol,* 275**,** H1441-8.

ZAZA, A., MALFATTO, G. & ROSEN, M. R. 1989. Electrophysiologic effects of ketanserin on canine Purkinje fibers, ventricular myocardium and the intact heart. *J Pharmacol Exp Ther,* 250**,** 397-405.

ZICHA, S., MALTSEV, V. A., NATTEL, S., SABBAH, H. N. & UNDROVINAS, A. I. 2004a. Post-transcriptional alterations in the expression of cardiac Na+ channel subunits in chronic heart failure. *J Mol Cell Cardiol,* 37**,** 91-100.

ZICHA, S., XIAO, L., STAFFORD, S., CHA, T. J., HAN, W., VARRO, A. & NATTEL, S. 2004b. Transmural expression of transient outward potassium current subunits in normal and failing canine and human hearts. *J Physiol,* 561**,** 735-48.
