# Supplementary material for: Pro-Arrhythmic Effects of Discontinuous Conduction at the Purkinje Fiber-Ventricle Junction Arising From Heart Failure-Induced Ionic Remodeling – Insights From Computational Modelling
Source: Front Physiol. 2022 Apr 25;13:877428. doi: 10.3389/fphys.2022.877428 (PMC9081695; doi:10.3389/fphys.2022.877428)
Supplement: Supplementary file 13 [file Image11.pdf]

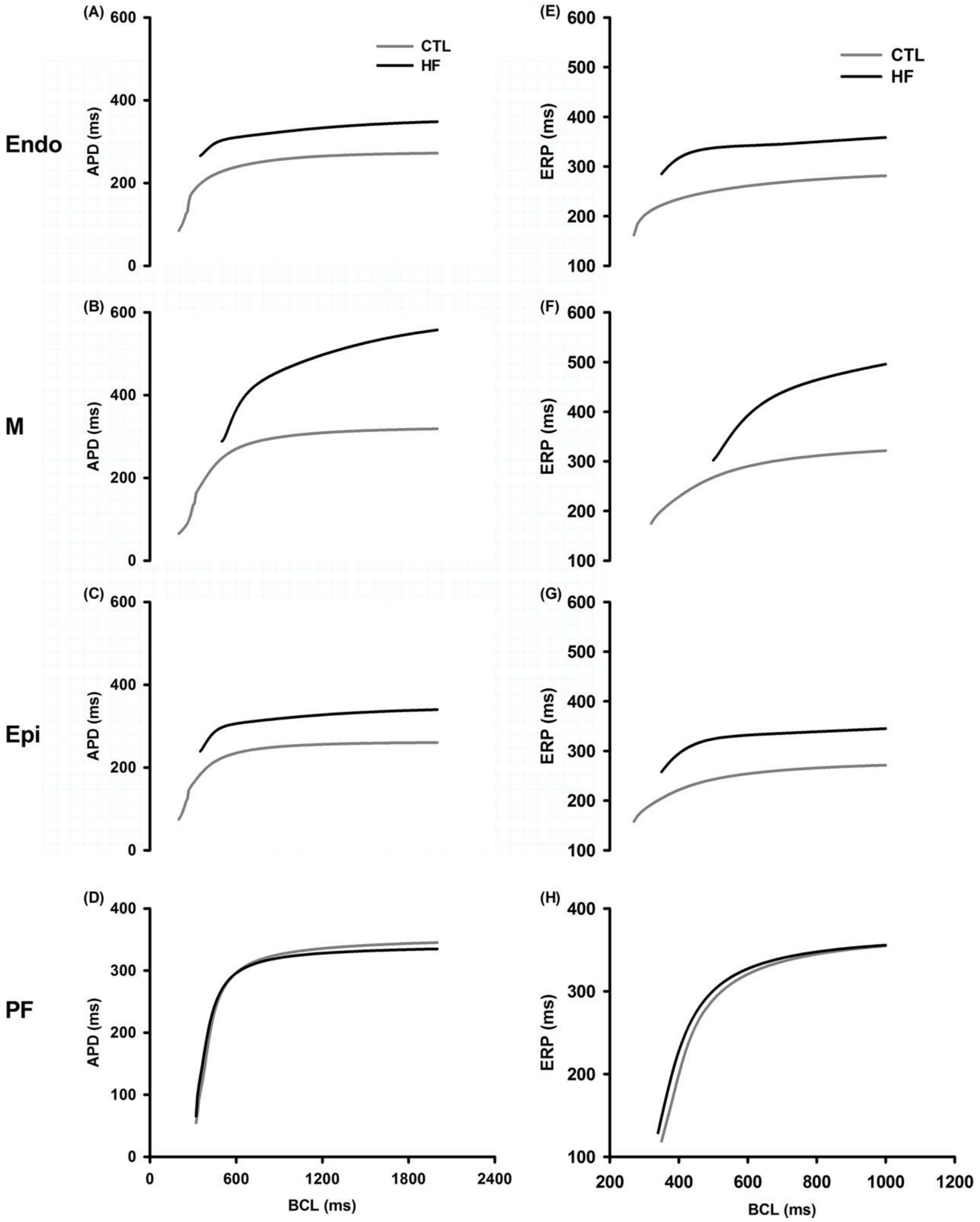

Supplementary Figure S11 APD restitution curves and ERP restitution curves. (A-D) Simulated APD restitution curves in CTL and HF conditions. (E-H) Computed ERP restitution curves in CTL and HF conditions. ERPs were estimated by measuring the shortest stimulation interval between S1-S2 stimulus, of which the OS of the S2-evoked AP reached 80% of that of the S1-evoked AP in the steady state (Workman et al., 2001).
