# Supplementary material for: Pro-Arrhythmic Effects of Discontinuous Conduction at the Purkinje Fiber-Ventricle Junction Arising From Heart Failure-Induced Ionic Remodeling – Insights From Computational Modelling
Source: Front Physiol. 2022 Apr 25;13:877428. doi: 10.3389/fphys.2022.877428 (PMC9081695; doi:10.3389/fphys.2022.877428)
Supplement: Supplementary file 14 [file Image7.pdf]

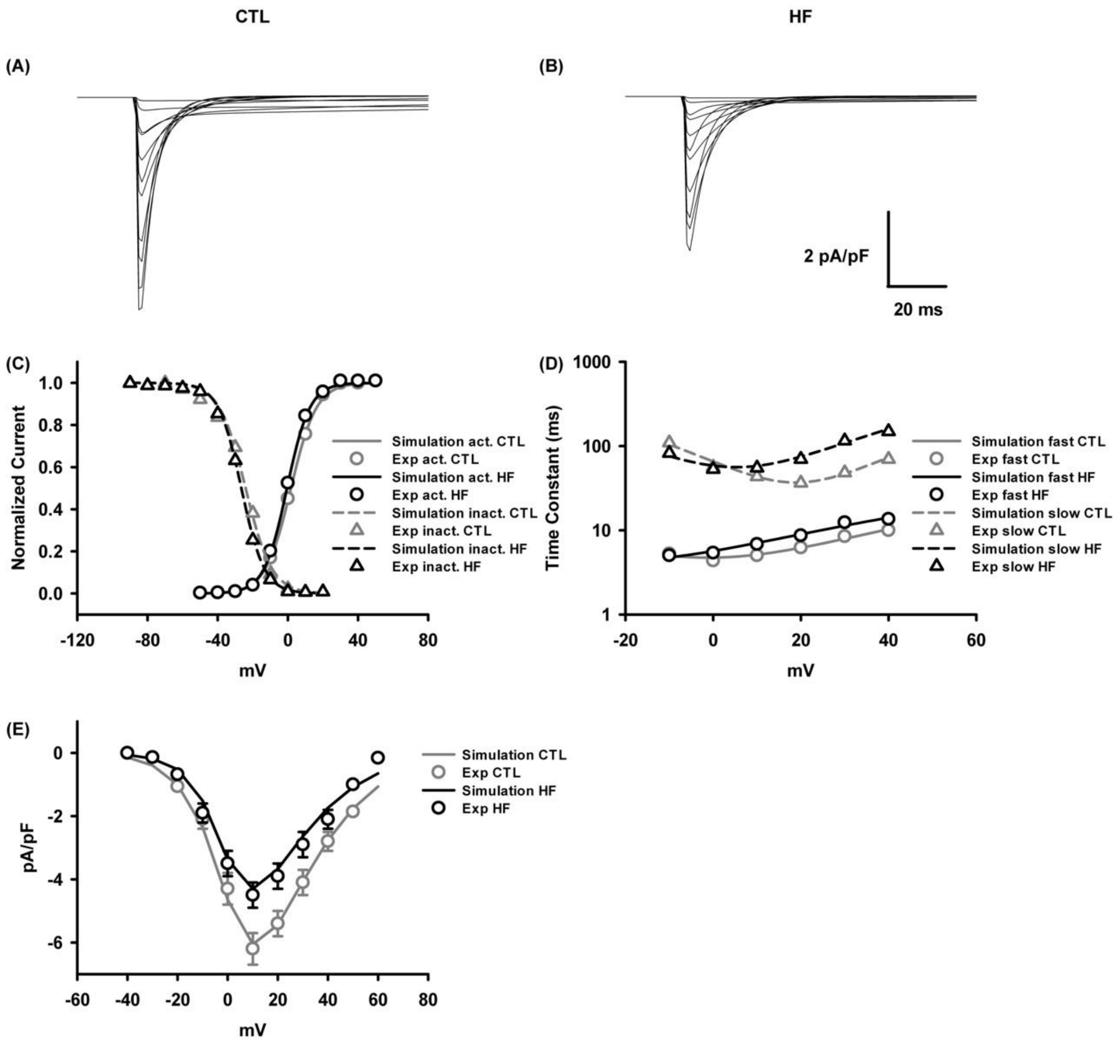

Supplementary Figure S7 Simulated I_{CaL} in PF.
Comparison of the simulated I_{CaL} properties in Purkinje Fiber in CTL and HF conditions. I_{CaL} was simulated during 1000-ms voltage-clamp pulses from -40 mV to +50 mV with a holding potential of -50 mV. The current traces of the CTL (A) and HF (B) conditions. (C) Simulated activation (Act.) and inactivation (Inact.) steady-state curves compared to the experimental data (Chen et al., 2002) (Han et al., 2001). (D) Simulated fast (τf) and slow (τf2) time constants compared to the experimental data. (E) Simulated I-V relationship compared to the experimental data.
