# Supplementary material for: Pro-Arrhythmic Effects of Discontinuous Conduction at the Purkinje Fiber-Ventricle Junction Arising From Heart Failure-Induced Ionic Remodeling – Insights From Computational Modelling
Source: Front Physiol. 2022 Apr 25;13:877428. doi: 10.3389/fphys.2022.877428 (PMC9081695; doi:10.3389/fphys.2022.877428)
Supplement: Supplementary file 15 [file Image1.pdf]

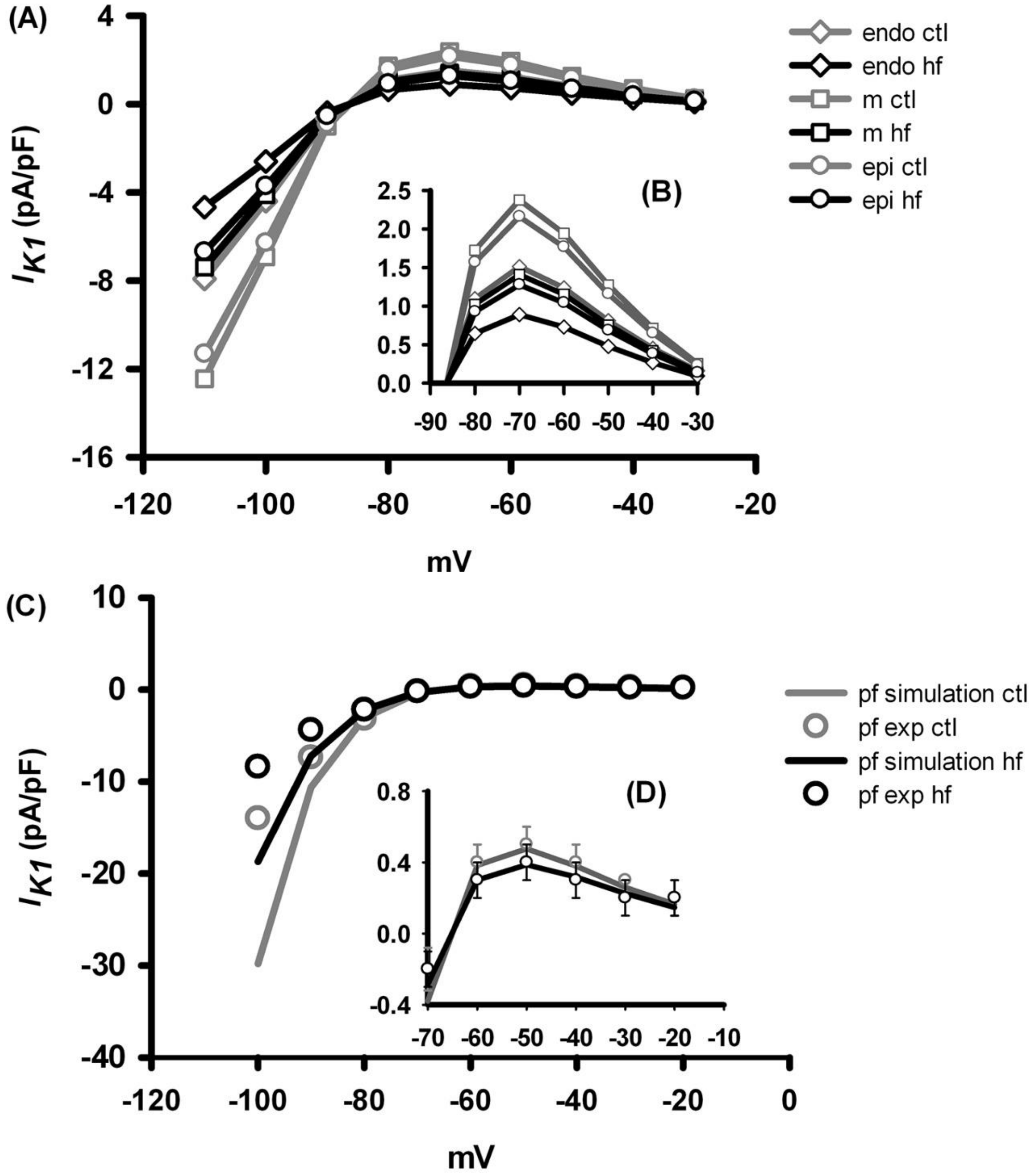

Supplementary Figure S1 Simulated I_{K1} in PF and ventricles. (A) Simulated I_{K1} and (B) simulated outward components of I_{K1}, with integrated heterogeneity in Endo, M and Epi cells. (C) Simulated I_{K1} and (D) simulated outward components of I_{K1}, in PF cells with experimental data (Han et al., 2001). All simulation in both CTL and HF conditions.
